# Pyroptosis-Related lncRNA Prognostic Model for Renal Cancer Contributes to Immunodiagnosis and Immunotherapy

**DOI:** 10.3389/fonc.2022.837155

**Published:** 2022-07-04

**Authors:** Xuan Zhou, Liangyu Yao, Xiang Zhou, Rong Cong, Jiaochen Luan, Xiyi Wei, Xu Zhang, Ninghong Song

**Affiliations:** ^1^ Department of Urology, The First Affiliated Hospital of Nanjing Medical University, Nanjing, China; ^2^ Department of Urology, The Affiliated Kizilsu Kirghiz Autonomous Prefecture People’s Hospital of Nanjing Medical University, Artux, China

**Keywords:** immune, tumor microenvironment, ccRCC, lncRNAs, pyroptosis

## Abstract

**Background:**

Renal clear cell cancer (ccRCC) is one of the most common cancers in humans. Thus, we aimed to construct a risk model to predict the prognosis of ccRCC effectively.

**Methods:**

We downloaded RNA sequencing (RNA-seq) data and clinical information of 539 kidney renal clear cell carcinoma (KIRC) patients and 72 normal humans from The Cancer Genome Atlas (TCGA) database and divided the data into training and testing groups randomly. Pyroptosis-related lncRNAs (PRLs) were obtained through Pearson correlation between pyroptosis genes and all lncRNAs (*p* < 0.05, coeff > 0.3). Univariate and multivariate Cox regression analyses were then performed to select suitable lncRNAs. Next, a novel signature was constructed and evaluated by survival analysis and ROC analysis. The same observation applies to the testing group to validate the value of the signature. By gene set enrichment analysis (GSEA), we predicted the underlying signaling pathway. Furthermore, we calculated immune cell infiltration, immune checkpoint, the T-cell receptor/B-cell receptor (TCR/BCR), SNV, and Tumor Immune Dysfunction and Exclusion (TIDE) scores in TCGA database. We also validated our model with an immunotherapy cohort. Finally, the expression of PRLs was validated by quantitative PCR (qPCR).

**Results:**

We constructed a prognostic signature composed of six key lncRNAs (U62317.1, MIR193BHG, LINC02027, AC121338.2, AC005785.1, AC156455.1), which significantly predict different overall survival (OS) rates. The efficiency was demonstrated using the receiver operating characteristic (ROC) curve. The signature was observed to be an independent prognostic factor in cohorts. In addition, we found the PRLs promote the tumor progression *via* immune-related pathways revealed in GSEA. Furthermore, the TCR, BCR, and SNV data were retrieved to screen immune features, and immune cell scores were calculated to measure the effect of the immune microenvironment on the risk model, indicating that high- and low-risk scores have different immune statuses. The TIDE algorithm was then used to predict the immune checkpoint blockade (ICB) response of our model, and subclass mapping was used to verify our model in another immunotherapy cohort data. Finally, qPCR validates the PRLs in cell lines.

**Conclusion:**

This study provided a new risk model to evaluate ccRCC and may be pyroptosis-related therapeutic targets in the clinic.

## Introduction

Renal cell cancer, one of the most common cancers in humans, accounts for the majority (90%) of kidney cancer cases. In Europe, there were 1.37 and 0.55 million estimated new kidney cancer cases in 2018 (1). Respectively, the numbers among Chinese humans were 0.67 and 0.23 million (2). Renal clear cell cancer (ccRCC) accounts for roughly 70% of all kidney cancers (3). In recent years, the diagnosis and treatment of ccRCC have significantly improved. However, considering the high morbidity and mortality of ccRCC, investigating prognostic factors that aid in risk stratification and further clinical decision making is of great value.

lncRNAs, defined as RNAs with a limited protein-coding potential of more than 200 nucleotides, have been reported to be aberrantly expressed in tumor tissues and can affect biological events such as embryonic development, cell growth, and cancer derivation and progression, including renal cancer (4). For example, a feed-forward loop between LncRNA-URRCC and EGFL7/P-AKT/FOXO3 signaling regulates ccRCC proliferation and metastasis both *in vivo* and *in vitro* (5). Knockdown of lncRNA UCA1 inhibited malignant phenotypes and Notch signaling by modulating the miR-182-5p/DLL4 axis (6). In addition, immune-related lncRNAs can be used as an effective biomarker to predict survival outcomes in ccRCC patients and is also widely used to construct prognostic markers (7–9).

Pyroptosis is mediated through inflammasomes and can trigger programmed cell death (10). Pyroptosis is characterized by cell swelling and large bubble swelling out of the plasma membrane (11). Both caspase-1 and caspase-4, caspase-5, and caspase-11 inflammasomes can induce pyroptosis (12). During cell pyroptosis, gasdermin D (GSDMD) is a key regulator (13). GSDMD can bind endosomal lipids and form membrane pores, causing cell pyroptosis reactions (14). The plasma membrane rupture during pyroptosis is associated with the formed membrane pores (15). Cell pyroptosis was initially used in the study of inflammation and infection. It was first observed in macrophages infected by Shigella in 1992 (16). In recent years, a great number of studies have shown that pyroptosis also plays an important role in cancer (17). Studies have found that the CARD-containing protein CARD8 mediates DPP8/9 inhibitor-induced pro-caspase-1-dependent pyroptosis in human myeloid cells, which could promote the treatment of acute myeloid leukemia (18). A recent study also confirmed the key role of pyroptosis in tumor immunity. Mutated BRAF and MEK inhibitors modulate the antitumor immune response by inducing tumor cell pyroptosis (19). Considering its crucial role in the pathogenesis and immunity of the cancers mentioned above, we aimed to research the function of ccRCC pyroptosis development.

However, although lncRNAs have been confirmed to play a certain regulatory role in pyroptosis (20, 21), there are still few studies on lncRNAs related to pyroptosis in renal cancer, and the specific regulatory mechanism and effect are still unclear. In our research, we identified pyroptosis-related lncRNAs (PRLs) from The Cancer Genome Atlas (TCGA) cohort and constructed a novel prognostic model, providing new insights into the diagnosis, evaluation, and treatment of ccRCC.

## Method

### Data Access

We downloaded gene expression data and the corresponding clinical information for the kidney renal clear cell carcinoma (KIRC) from TCGA data portal (https://tcga-data.nci.nih.gov/tcga/). The genes related to pyroptosis come from reviews and articles (22–25). We have listed the pyroptosis-related genes in **Table 1**. The data were then randomly divided into training (50%) and test (50%) sets.

**Table 1:** Pyroptosis-related genes.

Pyroptosis-related genes	
CASP4	
CASP1	
CASP5	
NLRC4	
NOD2	
PYCARD	
AIM2	
CASP9	
CASP8	
GSDMD	
NLRP2	
NOD1	
NLRP1	
NLRP3	
PLCG1	
CASP3	
GSDMB	
GSDMC	
IL18	
NLRP7	
NLRP6	
GSDMA	
GPX4	
IL1B	

### Identification of Different Expression of Pyroptosis-Related LncRNAs

The “limma” package and Perl script were used to annotate the downloaded data and identify the differentially expressed lncRNAs (DElncRNAs). The | logFC | >1 and FDR <0.05 were set as the threshold values.

### Establishment and Validation of the Pyroptosis-Related LncRNA Prognostic Model

We performed a univariate Cox regression analysis to find PRLs with survival differences. A logistic regression model with least absolute shrinkage and selection operator (LASSO) penalty was then performed to shrink the range. Next, a multivariate Cox regression was conducted to investigate the effects of multiple factors on KIRC.

According to the genomic PRLs which was identified above, a genome pyroptosis-related lncRNA signature (PRLncSig) can be described as follows:


$$\text{PRLncSig }\left(\text{Riskscore}\right)\;=\sum_{i=1}^{n}\text{exp(lncRNAi)}*\text{coef(lncRNAi)}$$

exp(lncRNAi) represents the expression level of selected lncRNAi in the patients, and coef(lncRNAi) represents the coefficient measured by multivariate Cox regression.

### Independent Prognostic Analysis and Multivariate Analysis of Prognostic Model

We used independent prognostic analysis to predict whether the prognostic model could be an independent prognostic factor in patients with KIRC. We then compared our prognostic model with the classical prognostic system, analyzed the prognostic factors using the ROC curve, and calculated the area under the ROC curve (AUC).

### Establishment of LncRNA-mRNA Coexpression Network

The correlation between lncRNAs and their target mRNAs was analyzed. To intuitively show the associations of the lncRNAs and mRNAs, we used alluvial plots implemented in the ggalluvial R package. The network results were visualized by Cytoscape (Cytoscape-3.8.2).

### Gene Set Enrichment Analysis

The GSEA 4.1 software was used to run gene set enrichment analysis. The training and testing gene sets that were used for the GSEA analysis were c2 KEGG gene sets (c2.cp.kegg. v7.4.symbols.gmt) from the Molecular Signatures Database (26).

### Estimation of Tumor-Infiltrating Immune Cells

To investigate the relationship between immune cell infiltration and risk score, we used the currently acknowledged methods, including TIMER, CIBERSORT, XCELL, QUANTISEQ, MCPcounter, EPIC, and CIBERSORT-ABS. Immune cell infiltration was estimated by the CIBERSORT algorithm (27). In addition, ssGSEA was used to quantify immune function between the high- and low-risk groups.

### Analysis of Immune Landscape and Prediction of Drug Sensitivity

To further explore the relationship between the immune landscape and the model in ccRCC, the value of aneuploidy, the richness of TCR and BCR, and the neoantigen load were calculated (28) (https://gdc.cancer.gov/about-data/publications/panimmune). All SNV data were retrieved from the BioMuta database (29). We then adopted the method of Ye et al. (30), analyzing the attained data, including TMB, PBRM1, BRCA2, GEP, neoantigen load, CYT, CTLA-4, PD-L1, and PD-L2 protein expression. The statistical significance of individual gene mutations was analyzed by Fisher’s exact test, and the statistical significance of other molecular characteristics was tested by a two-sided Wilcoxon’s test.

The risk model was then used to analyze the sensitivity of common antitumor drugs to ccRCC including axitinib, sunitinib, pazopanib, and sorafenib, by the “pRRophetic” package of the R software (31). Drug sensitivity was quantified by IC_50_. The lower the IC_50_, the higher the sensitivity.

### Validation of the Model in Immune Checkpoint Inhibitor Therapy

To validate the model’s potential clinical efficacy in predicting immune checkpoint inhibitor (ICI) treatment in different risk groups, we calculated Tumor Immune Dysfunction and Exclusion (TIDE) scores in different risk groups (32). Moreover, we used subclass mapping to validate our model in another published dataset of 47 melanoma patients that responded to immunotherapy (33).

### Cell Culture

Renal carcinoma cell lines 786-O, Caki-1, and normal kidney HK-2 cells were purchased from the Institute of Biochemistry and Cell Biology, Chinese Academy of Sciences (Shanghai, China). All cells were cultured in 1640 (RPMI-1640) (Gibco, Thermo Fisher Scientific, Gaithersburg, MD, USA) containing 10% fetal bovine serum (FBS; Biological Industries, Israel) and 1% penicillin/streptomycin (Gibco, Thermo Fisher Scientific, Gaithersburg, MD, USA). All cell lines were cultured at 37°C in a humidified incubator containing 5% CO_2_.

### RNA Isolation and qRT-PCR

Total RNA was isolated from cells by using Trizol reagent (Invitrogen, USA). cDNA was then synthesized using HiScript^®^ III All-in-one RT SuperMix Perfect for qPCR (Vazyme, China). qRT-PCR for mRNA was performed on the StepOne Plus Real-Time PCR system (Applied Biosystems, USA). Fold changes in mRNA expression were calculated using the 2^−ΔΔCt^ method and normalized against β-actin. PCR primer sequences were synthesized by TSINGKE Biological Technology (Nanjing, China) and are listed in **Supplementary Table S3**.

### Statistical Analysis

All statistical analyses were performed using R-version 4.1.0. We utilized the Pearson coefficient to evaluate the correlation between two continuous variables. Prognosis analysis was performed by Kaplan–Meier survival analysis. For qPCR, Student’s *t*-test was performed. *p* < 0.05 was considered statistically significant.

## Results

### Identification of Different Expressions of Pyroptosis-Related LncRNAs in the Training Set

The sequencing data of 539 KIRC patients and 72 normal people were downloaded from TCGA database. We then divided the samples into the training (50%) and testing (50%) groups. From the training data, we extracted the expression of 33 common pyroptosis-related genes. To figure out pyroptosis-related LncRNAs, we conducted a coexpression analysis. Next, the expression of pyroptosis-related LncRNAs was acquired. The datasets were subsequently screened using the limma package (corFilter = 0.3, pvalueFilter = 0.001). In total, 1,243 pyroptosis-related LncRNAs were selected and univariate Cox regression analysis was then performed. Following the univariate Cox regression analysis, LASSO regression was applied to avoid overfitting, and a multivariate Cox regression analysis was conducted. The results of univariate coregression and multivariate Cox regression analyses are shown in **Supplementary S1**, **S2**. After calculating the risk scores, the set was separated into high and low PRLncSig groups by using the median scores as a threshold. Six pyroptosis-related LncRNAs were screened out for further studies, including U62317.1, MIR193BHG, LINC02027, AC121338.2, AC005785.1, and AC156455.1.

### Construction and Internal Validation of the Prognostic Signature of 6 PRLs

**Figure 1:**
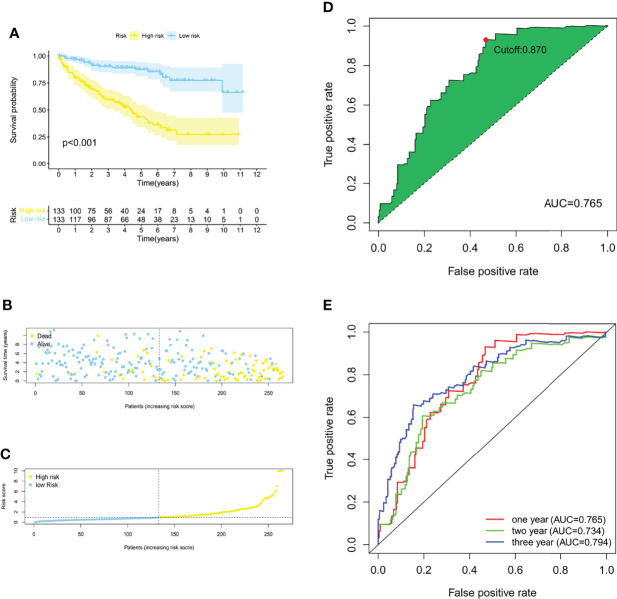
Construction of a risk score model of PRLs in the training group. **(A)** The Kaplan–Meier survival curves for the two risk groups in the training sets (*p* < 0.001). **(B)** The risk score plot of the training set. **(C)** Survival time and status of patients in the training set. **(D, E)** ROC curves at 1, 2, and 3 years, the AUC of the ROC curves, and the cutoff points for the risk model.

**Figure 2:**
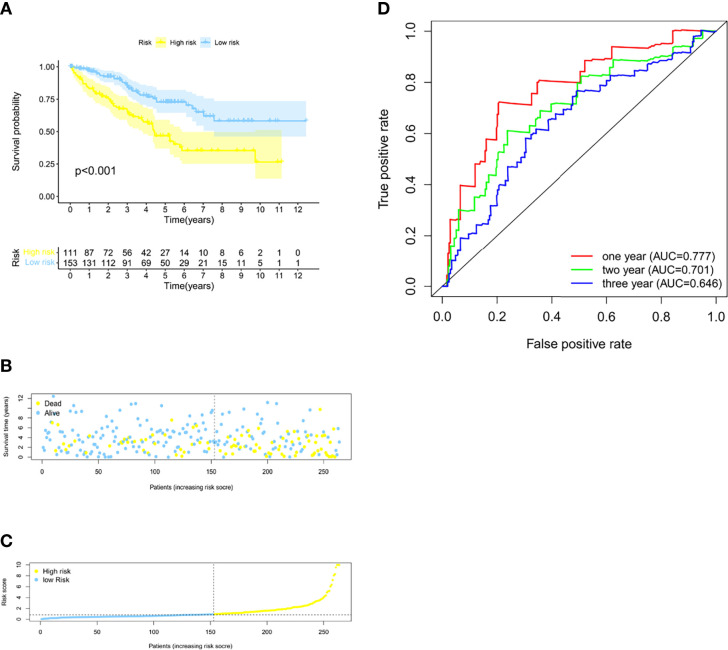
Validation of the model in the testing group. **(A)** The Kaplan–Meier survival curves for the two risk groups in the testing sets (*p* < 0.001). **(B)** The risk score plot of the testing set. **(C)** Survival time and status of patients in the testing set. **(D)** Time-dependent ROC curves of overall survival at 1, 2, and 3 years in the testing set.

With the 6 pyroptosis-related LncRNAs, we constructed a risk score model in the training set. The median risk score of the training cohort was used to divide the samples into a high- (RS > 1) or low-risk group (RS < 1). K-M survival analysis indicated that a high-risk score means poor prognosis both in the training set (**Figure 1A**). Whereas, the distribution of risk scores and the survival status of patients showed that increased risk scores may experience less survival time (**Figures 1B, C** **)**. The AUC was 0.765 (1 year), 0.734 (2 years), and 0.794 (3 years) (**Figure 1E**), and the cutoff value was 0.870 (**Figure 1E**). Meanwhile, the risk score model was validated in the testing set (**Figure 2**). The patients defined as the high-risk score group experience higher mortality and less survival time (**Figures 2A-C**). With the AUC being 0.777 (1 year), 0.701 (2 years), and 0.646 (3 years) (**Figure 2D**), the testing set demonstrated the efficiency of our model. Meanwhile, the calculation of the C-index also shows that our model has a certain predictive ability (**Supplementary Table S7**). We also verified the predictive power of our model with disease-free survival (DFS), progression-free interval (PFI), and disease-specific survival (DSS). The results showed that in the KIRC cohort, the high-risk group had a worse prognosis for disease-specific survival, progression-free interval, and disease-free survival.

### Correlation of the Prognostic Signature of 6 PRLs With Clinicopathological Features

**Figure 3:**
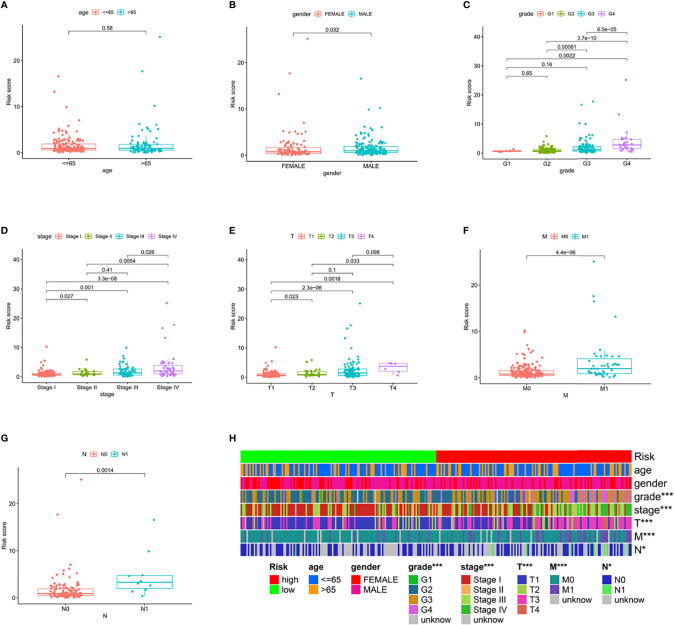
Clinical characteristic evaluation by the risk assessment model. **(A–G)** Different clinical characteristics, including **(A)** age, **(B)** gender, **(C)** tumor stage, **(D)** clinical stage, **(E)** T stage, **(F)** M stage, and **(G)** N stage assessed by the risk model. **(H)** A hot plot of clinical characteristics and risk scores.

We divided the groups into different characteristics, including age <65 and ≥65, women and men, stages I–II and III–IV, T1–2 and T3–4, N0 and N1, and M0 and M1. The clinicopathological characteristics of the risk model were analyzed by the Chi-square test, and the heatmap (**Figure 3H**) shows that there were significant differences between the high- and low-risk groups in tumor grade (*p* < 0.001), tumor stage (*p* < 0.001), T stage (*p* < 0.001), M stage (*p* < 0.001), and N stage (*p* < 0.05). We verified the effectiveness of this model in clinical tumor feature characteristics. The results are shown in **Figures 3A-G**. All these results indicate that the signature can be considered an effective clinical characteristic. Moreover, Cox regression analysis also showed that our prognostic model was an independent prognostic factor for overall survival at the training, testing, and overall data levels (**Supplementary Figures S2E–F**).

### Survival Analysis of Selected lncRNAs and Establishment of LncRNA-mRNA Coexpression Network

**Figure 4:**
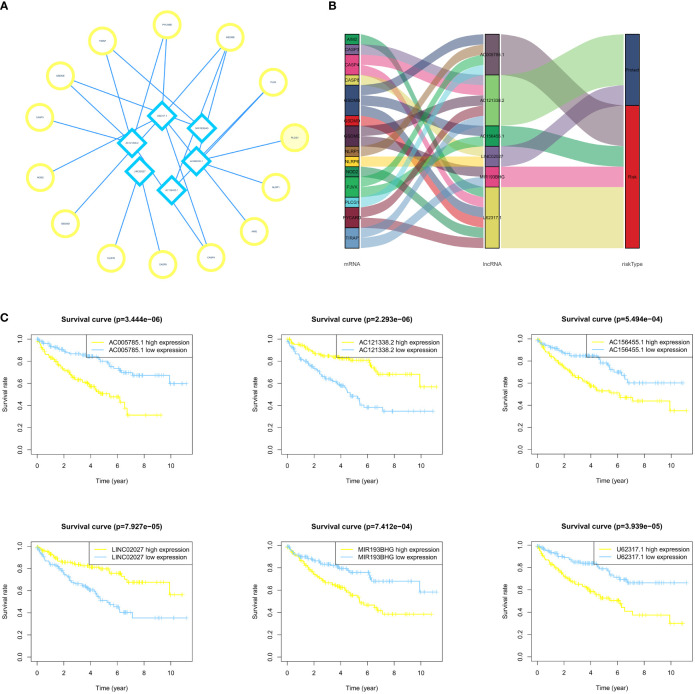
Establishment of the LncRNA-mRNA coexpression network and survival analysis. **(A, B)** The LncRNA-mRNA coexpression network indicates the interaction network between PRlncRNA and mRNA. **(C)** The survival curves of 6 PRlncRNAs in ccRCC patients show that there is an association between these lncRNAs and patient prognosis.

To examine the association between the expression level of each lncRNA in PRLncSig and the clinical outcomes, we constructed a Kaplan–Meier analysis and found that high expression of PRLncRNAs was correlated with poor prognosis of ccRCC patients (**Figure 4C**). We then conducted a lncRNA-mRNA coexpression network and visualized the result by using Cytoscape software version 3.8.2 (**Figure 4A**). Six lncRNAs and 14 pyroptosis-related mRNAs were used to build a coexpression network. The Sankey diagram showed the relationship between 15 lncRNAs and 21 pyroptosis-related mRNAs (**Figure 4B**).

### GSEA Analysis

**Figure 5:**
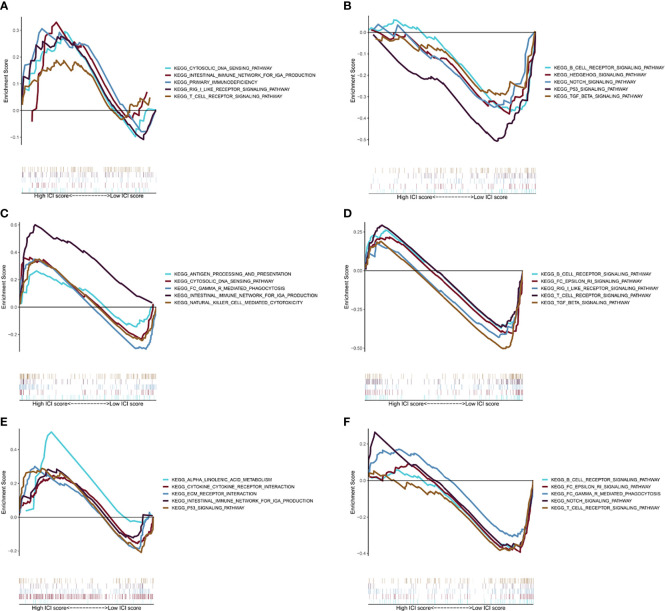
GSEA analysis. **(A)** The first five results of the enrichment plots of tumors with high-risk scores by GSEA in the training cohort. **(B)** The first five results of the enrichment plots of tumors with low-risk scores by GSEA in the training cohort. **(C)** The first five results of enrichment plots of tumors with high-risk scores by GSEA in the testing cohort. **(D)** The first five results of enrichment plots of tumors with low-risk scores by GSEA in the testing cohort. **(E)** The first five results of enrichment plots of tumors with high-risk scores by GSEA in the TCGA-KIRC cohort. **(F)** The first five results of the enrichment plots of tumors with low-risk scores by GSEA in the TCGA-KIRC cohort.

**Figures 5A, B** show KEGG pathways that were enriched in the training group, such as the cytosolic DNA sensing pathway, intestinal immune network for IgA production, T-cell receptor signaling pathway, RIG I-like receptor signaling pathway, primary immunodeficiency, B-cell receptor signaling pathway, Hedgehog signaling pathway, Notch signaling pathway, p53 signaling pathway, and TGF-β signaling pathway. While in the testing group, immune-related pathways were enriched, including the intestinal immune network for IgA production, cytosolic DNA sensing pathway, natural killer cell-mediated cytotoxicity, Fc gamma R-mediated phagocytosis, antigen processing and presentation, B-cell receptor signaling pathway, T-cell receptor signaling pathway, RIG I-like receptor signaling pathway, and Fc Epsilon RI signaling pathway (**Figures 5C, D** **)**. Overall, TCGA cohort also obtained a similar result (**Figures 5E, F** **)**. All these pathways suggest a close link with the immune system, which may play an important role in the occurrence, progression, and metastasis of ccRCC (34).

### Analysis of Cancer Immune Microenvironment

**Figure 6:**
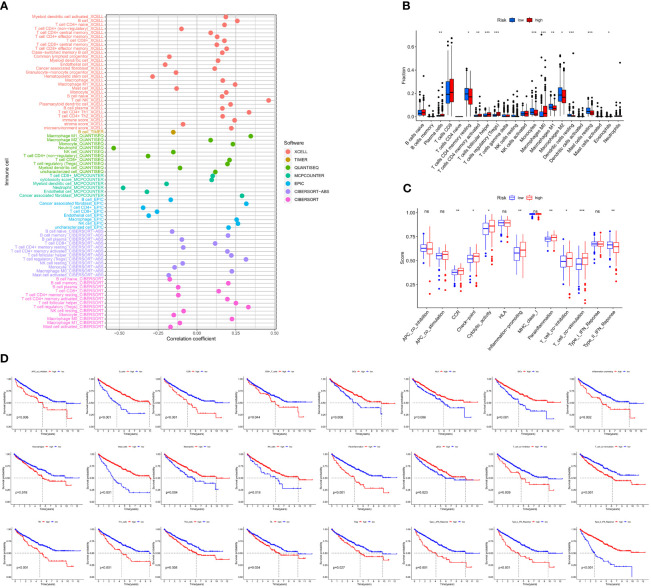
Correlation of immune microenvironment between risk scores. **(A)** Correlation analysis of the risk score and immune cells by XCELL, TIMER, QUANTISEQ, MCPCOUNTER, EPIC, CIBERSORT abs, and CIBERSORT algorithm. **(B)** Boxplot of CIBERSORT immune cell infiltrate analysis in TCGA-KIRC cohort. **(C)** Boxplot of immune function scores between high- and low-risk groups. **(D)** K-M curve of immune function between high- and low-risk groups (including APC coinhibition, B cells, CCR, CD8+ T cells, DCs, HLA, iDCs, inflammation-promoting, macrophages, mast cells, neutrophils, NK cells, parainflammation, pDCs, T-cell coinhibition, T-cell costimulation, Tfh, Th1 cells, Th2 cells, TIL, Treg, type I IFN response, type I IFN response, and type II IFN response).

We used 7 different algorithms (29–31) to calculate the infiltration of immune cells in TCGA cohort. **Figure 6A** and **Supplementary Figures S1A–H** demonstrate the pattern of immune cell infiltration of the risk model in TCGA cohort. The findings indicated that there was a positive relationship between immune cell infiltration and risk score (**Figure 6A**). The results of CIBERSORTR (**Figure 6B**) showed the differentially expressed immune cells in the high- and low-risk score groups, such as plasma cells, CD4 memory T cells, T-cell follicular helper, regulatory T cells (Tregs), monocytes, macrophages M0, macrophages M1, macrophages M2, dendritic cell resting, mast cell resting, and eosinophils. Further analysis of immune signatures demonstrated the differences in CCR, checkpoint, cytolytic activity, inflammation-promoting, parainflammation, T-cell coinhibition, T-cell costimulation, and type II IFN response between two risk groups (*p* < 0.05). Moreover, survival analysis of immune signatures in TCGA cohort is shown in **Figure 6C**. The single most striking observation to emerge from the survival analysis was that a high-risk score represents a low survival probability.

### PRLs Are Strongly Associated With Checkpoints and Immune-Associated Cells in ccRCC

**Figure 7:**
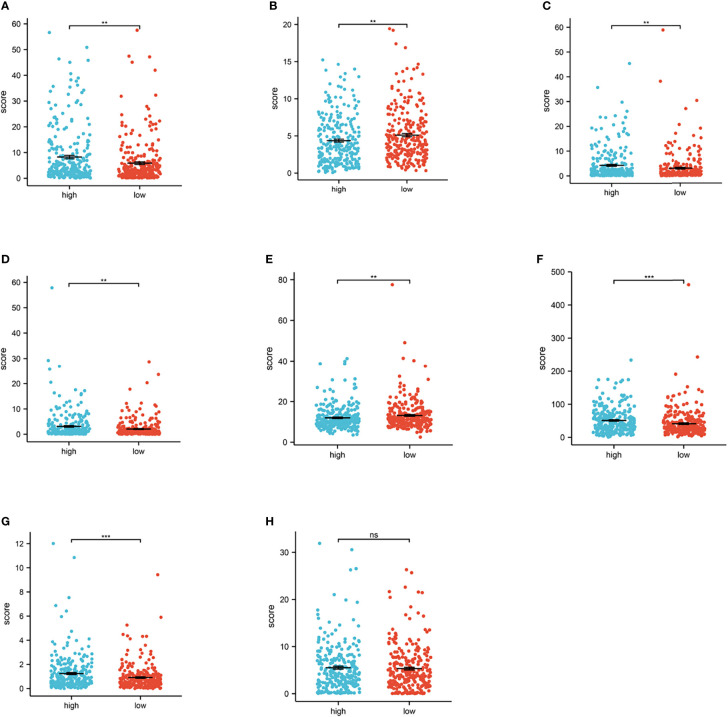
Relationship between risk score and immune checkpoint. **(A–H)** High-risk scores were positively correlated with high expression of CD27 (*p* < 0.01, **Figure 6A**), LAG3 (*p* < 0.01, **Figure 6C**), PDCD1 (*p* < 0.01, **Figure 6D**), and TNFRSF12A (*p* < 0.01, **Figure 6F**), TNFRSF18 (*p* < 0.001, **Figure 6G**) and low expression of CEACAM1 (*p* < 0.01, **Figure 6B**) and PVR (*p* < 0.01, **Figure 6E**), while TNFSF9 (**Figure 6H**) shows no statistical significance (^*^ *p* < 0.0, ^**^ *p* < 0.001, ^***^ *p* < 0.001).

In clinical practice, ICI therapy is considered an important treatment method for carcinoma. It is a series of molecules that generate costimulatory or inhibitory signals in the immune response. Thus, we tested ICI-related biomarkers to figure out the relationship between the risk model and these biomarkers. We discovered that high-risk scores represented high expression of CD27 (*p* < 0.01, **Figure 7A**), LAG3 (*p* < 0.01, **Figure 7C**), PDCD1 (*p* < 0.01, **Figure 7D**), and TNFRSF12A (*p* < 0.01 **Figure 7F**) and low expression of CEACAM1 (*p* < 0.01, **Figure 7B** **)** and PVR (*p* < 0.01, **Figure 7E**), while the latter (**Figure 6H**) showed no statistical differences.

**Figure 8:**
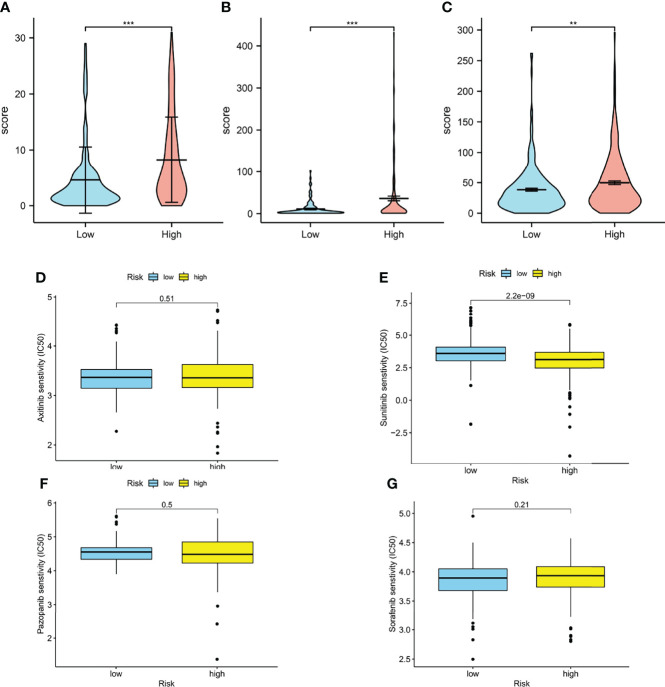
Immune-related biomarkers and drug response. **(A)** Aneuploidy scores of high- and low-risk groups (*p* < 0.001). **(B)** TCR (*p* < 0.001) and BCR (*p* < 0.01) scores of high- and low-risk groups. **(D–G)** Boxplots of estimated half-maximal inhibitory concentration (IC_50_) of the high- and low-risk groups for antitumor drugs: axitinib (**D**, *p* = 0.51), sunitinib (**E**, *p* < 0.001), pazopanib (**F**, *p* = 0.5), and sorafenib (**G**, *p* = 0.21) (^*^ *p* < 0.0, ^**^ *p* < 0.001, ^***^ *p* < 0.001).

The aneuploidy, TCR, BCR, and neoantigen were also calculated. As illustrated in the cartoon (**Figures 8A-C**), the high-risk group has lower scores in the aneuploidy and richness of TCR, BCR, and neoantigen. The TCR, BCR, aneuploidy, neoantigen, and TMB scores are shown **in** **Supplementary Tables S4**, **S5**.

### Prediction of Drug Response

Interestingly, in our model, the IC_50_ of sunitinib in the high-risk group was significantly lower than that in the low-risk group, which means higher sensitivity, implying that patients in the high-risk groups were more likely to develop chemoresistance (**Figures 8D-I**).

### Validation of the Model in Immune Therapy Response

We used the TIDE to evaluate the potential therapeutic effect of immunotherapy in the high- and low-risk groups. The result showed that in TCGA cohort, the high-risk group had higher TIDE scores (*p* < 0.001), which means high-risk patients were less likely to benefit from ICI therapy (**Supplementary Figure S3A**). Meanwhile, the MSI scores of the high-risk group were lower, but T-cell exclusion and T-cell dysfunction were higher (*p* < 0.001) (**Supplementary Figures S3B, D**). All the results demonstrated that our model had a good effect in predicting immunotherapy, T-cell dysfunction, and the exclusion of ccRCC. Furthermore, we also validated our prognostic model against another published immunotherapy data set by using subclass mapping and found that CTAL4 nonresponsiveness was more likely to occur in the high-risk group (*p* = 0.039, *p* = 0.0319) (**Supplementary Figure 3E**). These results indicate that low-risk patients based on PRLs may benefit from immunotherapy.

### Validation of PRLs in Indicated Cell Lines by Real-Time qPCR

**Figure 9:**
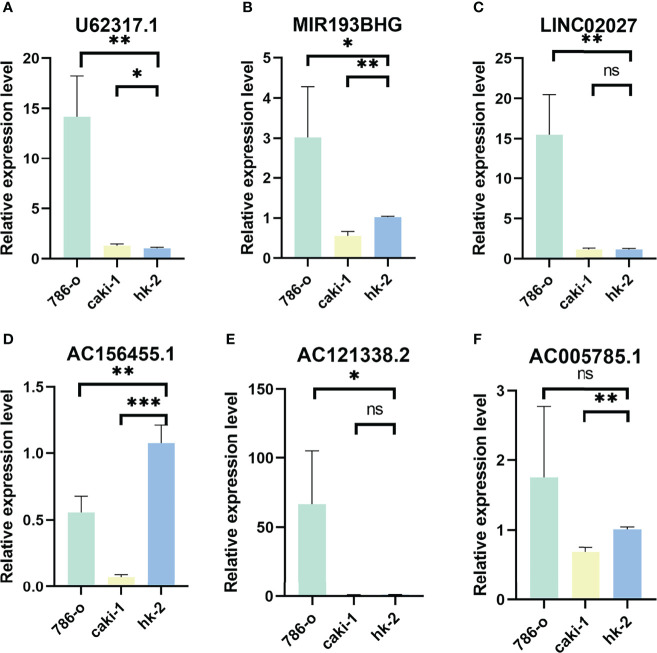
Validation of results through quantitative PCR. Relative expression of 6 PRLs in three cell lines (786-O, Caki-1, HK-2): **(A)** U62317.1, **(B)** MIR193BHG, **(C)** LINC02027, **(D)** AC156455.1, **(E)** AC121338.2, and **(F)** AC005785.1 (^*^ *p* < 0.0, ^**^ *p* < 0.001, ^***^ *p* < 0.001).

We verified the expression of PRLs in the cell lines mentioned above by qPCR, and the results are shown in **Figures 9A–E**, which found that the expression of six types of PRL was elevated in renal cancer cells. LncRNA U62317.1, MIR193BHG, AC121338.2, and LINC02027 were highly expressed in tumor cells, while AC156455.1 showed the opposite trend.

## Discussion

Recently, as we learn more about the tumor microenvironment and the immune system, significant advancements have occurred in cancer immunotherapy. In particular, immune-related genes have offered the potential for the identification of new molecular targets for cancer immunotherapy (35). Clear cell renal cell carcinoma is one of the most common renal carcinoma in China (2), and immunotherapy is clinically effective and thus a promising therapeutic option for ccRCC (36).

Pyroptosis is an inflammatory response-related form of cell death that has recently been found in tumor chemotherapy drug therapy (12). In our study, we first screened for 34 pyroptosis-related genes, then the data were randomly divided into training (50%) and testing (50%) groups. We acquired the data of lncRNAs from the received pyroptosis-related genes and calculated the coexpression coefficient to select differentially expressed PRLs. Secondly, we constructed a risk model by using univariate Cox analysis, LASSO regression analysis, and multivariate Cox regression analysis. Six PRLs were identified, including U62317.1, MIR193BHG, LINC02027, AC121338.2, AC005785.1, and AC156455.1, which have a prognostic significance, and the relationship interaction network was established. A risk score model was constructed based on these lncRNAs, and patients were divided into the high-risk and the low-risk groups. Survival analysis showed that the low-risk subgroup had a much better prognosis. Next, we evaluated the relationship between clinical characteristics and the risk scores, the prognostic analysis showed that the model-based risk score can be a good indicator of the characteristics of the KIRC. Then, we further analyzed 6 PRLs by using the GSEA algorithm, and the pathway enriched indicated that the selected lncRNAs had a close association with immune infiltration. This means that our model may well reflect the immune difference between the high- and low-risk groups.

Recent studies have indicated that tumor cells undergoing pyroptosis recruit tumor-suppressed immune cells (37, 38). Therefore, we calculated the correlation coefficient of immune cell infiltration by using various algorithms. According to the results, the high-risk group was more negatively associated with tumor-infiltrating immune cells such as CD4+ T cells, hematopoietic cells, no regulatory CD4+ T cells, and neutrophil cells. Currently, some studies found that neutrophils may drive unconventional T cells, mediate resistance against human tumors (39), and regulatory T cells may acquire cytotoxic function by CD4+ T cells to enhance antitumor activity (40). Therefore, we believed that the model constructed has the potential to determine new biomarkers for further study.

We then investigated the relationship between immune infiltration and the model in renal clear cell carcinoma. Interestingly, we found that the higher scores possessed higher expression levels of immune checkpoints such as CD27, LAG3, PDCD1, TNRSF12A, and TNRSF18, which may represent immunological resistance and poor prognosis.

Next, the aneuploidy value and the richness of TCR, BCR, and neoantigen were calculated, and patients had higher richness with higher risk scores. We also tested the drug sensitivity of ccRCC by the “pRRophetic” package and found patients more likely to develop chemoresistance with higher scores.

Furthermore, we also calculated the TIDE scores, the MSI scores, T-cell exclusion, and T-cell dysfunction of the high- and low-risk groups. In our model, a higher risk group means higher TIDE scores, higher T-cell exclusion, higher T-cell dysfunction, and lower MSI scores. To further validate, we used published immunotherapy-based data to validate our model’s evaluation of immunotherapy. Subclass mapping was used to compare the high- and low-risk groups in the cohort. Results demonstrated that the high-risk group was more likely to have unresponsive CTAL4. Therefore, it is reasonably hypothesized that higher risk scores mean less response to immunotherapy.

After integrating and analyzing these results, we discovered that our model may be valid to evaluate tumor immune cell infiltration and tumor immune microenvironment and may help and guide the immunotherapy of renal cancer.

However, our research still has certain limitations. We only used the transcriptome data acquired from TCGA. It might be better to have other database validations. Moreover, the limited sample size in TCGA may cause possible bias in the results, and further research is needed to confirm these findings. Since we only used two renal cancer cell lines, the correlation between the clear cell renal cancer and normal cell lines needs to be further verified. Further experiments should be performed to verify our results.

## Data Availability Statement

The original contributions presented in the study are included in the article/**Supplementary Material**. Further inquiries can be directed to the corresponding author.

## Ethics Statement

Ethical approval/written informed consent was not required for the study of animals/human participants in accordance with the local legislation and institutional requirements.

## Author Contributions

NS designed this work. XuZ, LY, and XiZ wrote the manuscript. XuZ, RC, and JL performed the bioinformatics analysis. XZh and XW performed the data review. All authors have read and approved the manuscript.

## Funding

This article was funded by the National Natural Science Foundation of China [grant number: 81871151; 82071638].

## Conflict of Interest

The authors declare that the research was conducted in the absence of any commercial or financial relationships that could be construed as a potential conflict of interest.

## Publisher’s Note

All claims expressed in this article are solely those of the authors and do not necessarily represent those of their affiliated organizations, or those of the publisher, the editors and the reviewers. Any product that may be evaluated in this article, or claim that may be made by its manufacturer, is not guaranteed or endorsed by the publisher.

## Supplementary Material

Supplementary Figure 1 The immune cell infiltration of different risk score groups, which calculated by the TIMER, CIBERSORT, XCELL, QUANTISEQ, MCPcounter, EPIC, and CIBERSORT-ABS algorithm **(A)**: CIBERSORT; **(B)**: CIBERSORT-ABS; **(C)**: QUANTISEQ; **(D-E)**: XCELL; **(F)**: EPIC; **(G)**: MCPCOUNTER; **(H)**: TIMER).

Supplementary Figure 2 **(A)**: Disease-Free Survival curve of the two groups; **(B)**: Disease-Specific Survival curve of the two groups; **(C)**: Progress Free Interval curve of the two groups; **(D)**: AUC of **(E-F)**: Univariate and multivariate Cox regression analysis for ccRCC with clinicopathological factors in the TCGA data.

Supplementary Figure 3 **(A-D)**: Scores of TIDE, MSI, and T-cell exclusion and dysfunction in different risk groups (*** p < 0.001); **(E)**: Heatmap of Subclass mapping analysis for predicting the likelihood of response to ICI therapy of different risk score groups.

## Abbreviations

lncRNAs, long noncoding RNAs; ccRCC, clear cell renal cell carcinoma; ROC, time-dependent receiver operating characteristic; KIRC, kidney renal clear cell carcinoma; HR, hazard ratio; PRLncSig, pyroptosis-related lncRNA signature; PRLs, pyroptosis-related lncRNAs; ICB, immune checkpoint blockade; RCC, renal cell carcinoma; ccRCC, clear cell renal cell carcinoma; ESTIMATE, Estimation of STromal and Immune cells in MAlignant Tumor tissues using Expression data; LASSO, least absolute shrinkage and selection operator; CI, confidence interval; GO, Gene Ontology; KEGG, Kyoto Encyclopedia of Genes and Genomes pathway; GSEA, gene set enrichment analysis; DFS, disease-free survival; PFI, progression-free interval; DSS, disease-specific survival; TCR, T-cell receptor; BCR, B-cell receptor.
