# Supplementary figures and images for: Pyroptosis-Related lncRNA Prognostic Model for Renal Cancer Contributes to Immunodiagnosis and Immunotherapy
Source: Front Oncol. 2022 Jul 4;12:837155. doi: 10.3389/fonc.2022.837155 (PMC9291251; doi:10.3389/fonc.2022.837155)

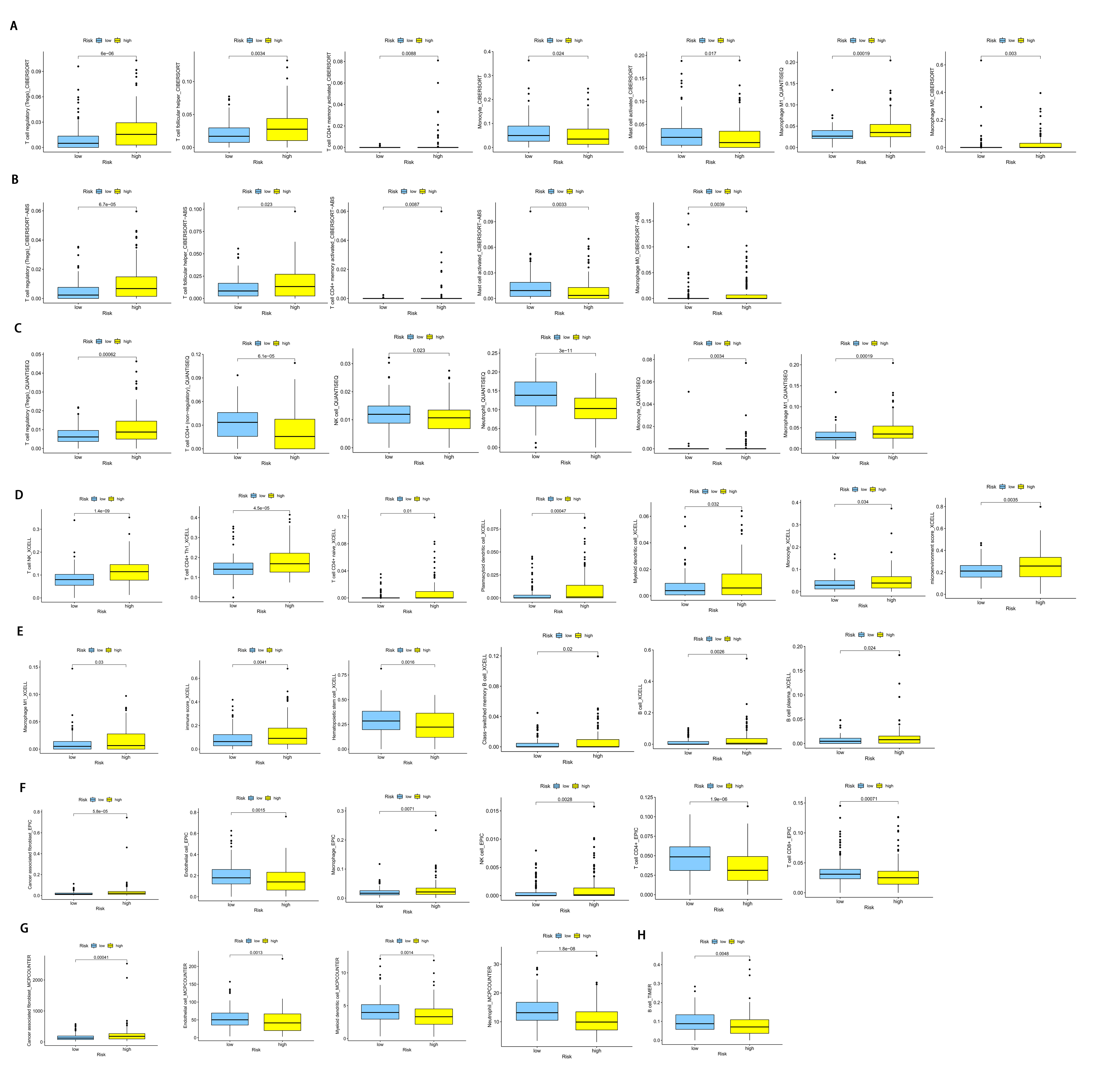

Supplement: Supplementary Figure 1 — The immune cell infiltration of different risk score groups, which calculated by the TIMER, CIBERSORT, XCELL, QUANTISEQ, MCPcounter, EPIC, and CIBERSORT-ABS algorithm (A): CIBERSORT; (B): CIBERSORT-ABS; (C): QUANTISEQ; (D-E): XCELL; (F): EPIC; (G): MCPCOUNTER; (H): TIMER). [file Image_1.tif]

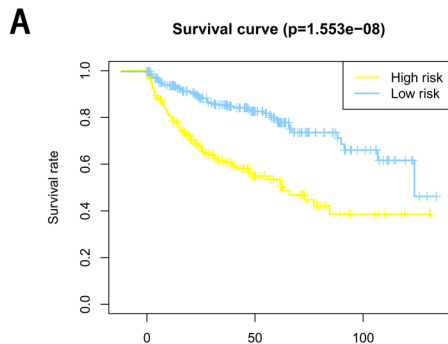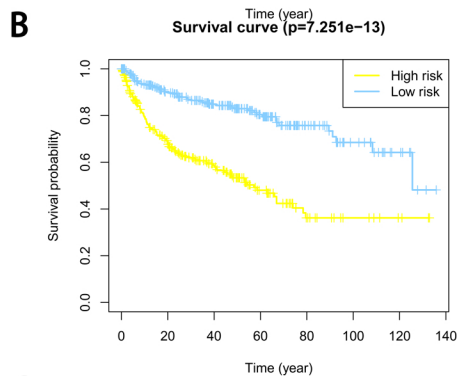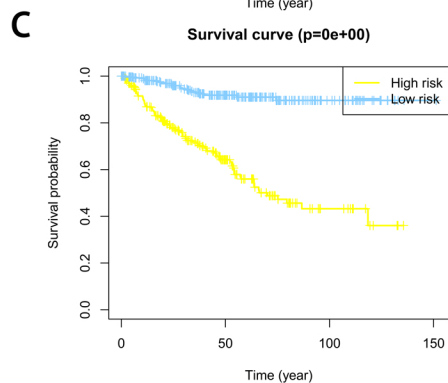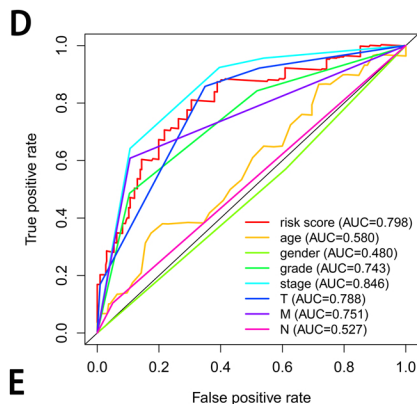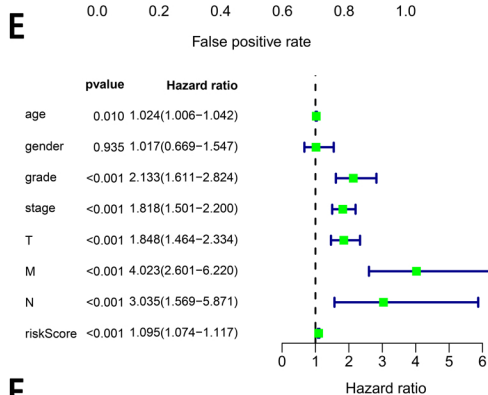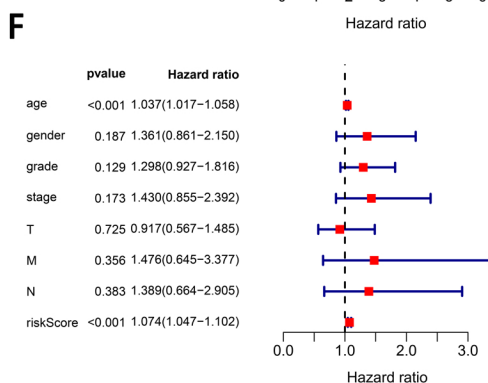

Supplement: Supplementary Figure 2 — (A): Disease-Free Survival curve of the two groups; (B): Disease-Specific Survival curve of the two groups; (C): Progress Free Interval curve of the two groups; (D): AUC of (E-F): Univariate and multivariate Cox regression analysis for ccRCC with clinicopathological factors in the TCGA data. [file Image_2.pdf]

**A**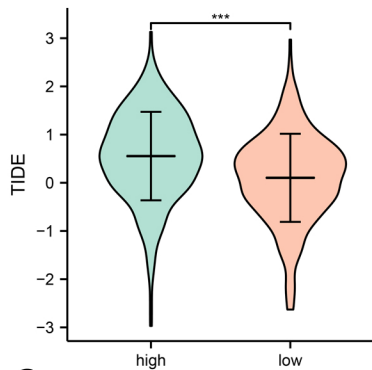**B**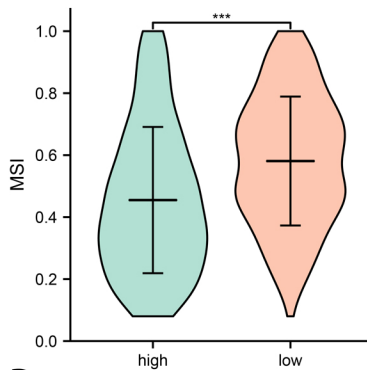**C**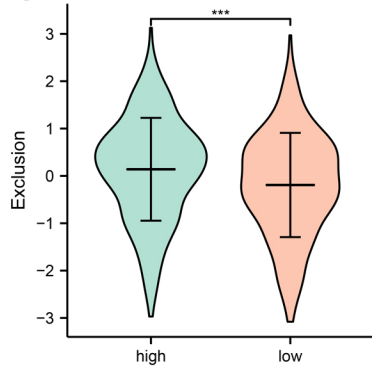**D**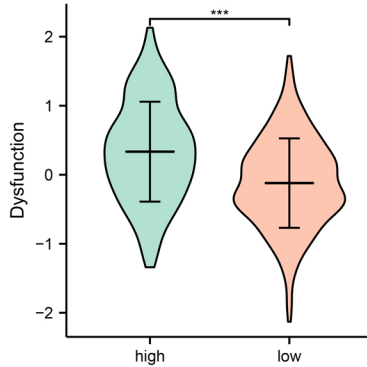**E**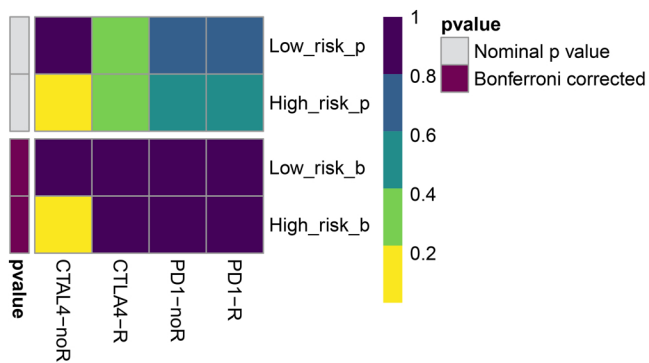

Supplement: Supplementary Figure 3 — (A-D): Scores of TIDE, MSI, and T-cell exclusion and dysfunction in different risk groups (*** p < 0.001); (E): Heatmap of Subclass mapping analysis for predicting the likelihood of response to ICI therapy of different risk score groups. [file Image_3.pdf]
