# Supplementary material for: Pyroptosis-Related lncRNA Prognostic Model for Renal Cancer Contributes to Immunodiagnosis and Immunotherapy
Source: Front Oncol. 2022 Jul 4;12:837155. doi: 10.3389/fonc.2022.837155 (PMC9291251; doi:10.3389/fonc.2022.837155)
Supplement: Supplementary file 4 [file Table_1.docx]

**Supplementary Table S1 univariate cox regression analysis of training set**

| **id** | **HR** | **HR.95L** | **HR.95H** | **pvalue** |
| --- | --- | --- | --- | --- |
| AC002401.3 | 0.937578 | 0.774101 | 1.135578 | 0.509667 |
| LINC00671 | 0.99346 | 0.979478 | 1.007643 | 0.364283 |
| AP000757.2 | 1.004879 | 0.977362 | 1.033171 | 0.731173 |
| FGD5-AS1 | 0.975085 | 0.944267 | 1.006909 | 0.123622 |
| AC000123.1 | 1.350595 | 1.136551 | 1.60495 | 0.00064 |
| HCG27 | 1.1158 | 0.98255 | 1.267121 | 0.091284 |
| AC124798.1 | 0.948541 | 0.844776 | 1.065051 | 0.371447 |
| AL139287.1 | 1.056047 | 1.01014 | 1.10404 | 0.016176 |
| AL360181.2 | 1.167178 | 1.075583 | 1.266574 | 0.000209 |
| MAPKAPK5-AS1 | 1.079357 | 1.003504 | 1.160943 | 0.03997 |
| LINC01770 | 1.353699 | 1.161095 | 1.578252 | 0.00011 |
| AL132712.1 | 1.178871 | 1.007855 | 1.378907 | 0.039609 |
| NEAT1 | 1.003008 | 0.998402 | 1.007636 | 0.200904 |
| AL157392.3 | 1.372426 | 0.948622 | 1.985569 | 0.092947 |
| AP002840.2 | 0.773125 | 0.610798 | 0.978594 | 0.032361 |
| LENG8-AS1 | 1.142411 | 1.070787 | 1.218825 | 5.57E-05 |
| AC007406.3 | 0.953681 | 0.87743 | 1.036559 | 0.264662 |
| AL132657.1 | 1.055003 | 0.763231 | 1.458315 | 0.745817 |
| LINC01532 | 0.955109 | 0.847863 | 1.075921 | 0.449773 |
| AC092171.4 | 1.673049 | 1.363632 | 2.052675 | 8.12E-07 |
| AC104211.2 | 0.69465 | 0.521998 | 0.924407 | 0.012451 |
| AC015922.2 | 0.924752 | 0.870359 | 0.982544 | 0.011427 |
| AL354733.3 | 1.178201 | 1.001822 | 1.385632 | 0.047483 |
| AC002128.1 | 1.417833 | 1.060422 | 1.895709 | 0.018481 |
| AC010776.3 | 0.983544 | 0.940135 | 1.028957 | 0.471225 |
| LINC01138 | 1.677773 | 1.368862 | 2.056396 | 6.22E-07 |
| ZNF436-AS1 | 1.321255 | 1.131702 | 1.542558 | 0.000422 |
| AC100830.2 | 1.024913 | 0.809718 | 1.297299 | 0.837851 |
| AC116667.1 | 1.626949 | 1.153798 | 2.294131 | 0.005505 |
| AC124319.2 | 1.135071 | 0.895543 | 1.438664 | 0.294793 |
| HOTAIRM1 | 1.141206 | 1.095012 | 1.189348 | 3.72E-10 |
| LINC02084 | 1.305013 | 1.112541 | 1.530783 | 0.001076 |
| AC005104.1 | 1.369807 | 1.164888 | 1.610774 | 0.000141 |
| ANKRD10-IT1 | 1.051141 | 1.01666 | 1.086793 | 0.00338 |
| SNHG16 | 1.115261 | 0.991819 | 1.254067 | 0.068349 |
| OLMALINC | 1.027318 | 0.952849 | 1.107606 | 0.482695 |
| AC084117.1 | 1.133743 | 1.064752 | 1.207205 | 8.90E-05 |
| U47924.2 | 1.380527 | 1.147122 | 1.661423 | 0.000644 |
| AC064807.1 | 0.474475 | 0.281728 | 0.799092 | 0.005059 |
| AC008966.2 | 0.893397 | 0.514807 | 1.550404 | 0.68857 |
| PSMB8-AS1 | 1.033663 | 0.984507 | 1.085273 | 0.182913 |
| CPNE8-AS1 | 1.161398 | 1.010188 | 1.33524 | 0.035518 |
| AC015977.2 | 0.997437 | 0.808859 | 1.229982 | 0.980855 |
| AC007953.1 | 1.018541 | 0.956812 | 1.084253 | 0.564655 |
| AL161421.1 | 0.96602 | 0.741447 | 1.258612 | 0.797878 |
| PTOV1-AS1 | 1.54883 | 1.25409 | 1.912841 | 4.86E-05 |
| AL157392.4 | 1.518966 | 1.165757 | 1.979193 | 0.001963 |
| GABPB1-AS1 | 1.218874 | 1.070234 | 1.388157 | 0.002855 |
| AC129507.3 | 0.591902 | 0.399223 | 0.877574 | 0.009057 |
| LINC00861 | 1.346854 | 1.029911 | 1.761334 | 0.029611 |
| AL008582.1 | 1.618946 | 1.297002 | 2.020804 | 2.06E-05 |
| HOXB-AS3 | 0.973962 | 0.86871 | 1.091966 | 0.651158 |
| HM13-IT1 | 1.460366 | 1.25935 | 1.693468 | 5.39E-07 |
| LINC02100 | 1.464894 | 1.238765 | 1.7323 | 8.09E-06 |
| AC039056.2 | 1.618759 | 1.196806 | 2.189478 | 0.001773 |
| AC026471.4 | 1.155354 | 1.087261 | 1.227711 | 3.17E-06 |
| LINC01594 | 1.180325 | 0.942513 | 1.478141 | 0.148678 |
| AC004148.1 | 1.266585 | 1.12354 | 1.427844 | 0.000111 |
| TTC28-AS1 | 1.311632 | 1.01027 | 1.70289 | 0.041682 |
| LINC02166 | 1.137051 | 0.948473 | 1.363123 | 0.16508 |
| LINC00989 | 0.882436 | 0.585518 | 1.329921 | 0.550103 |
| YTHDF3-AS1 | 1.182677 | 0.848166 | 1.649117 | 0.322601 |
| AC087222.1 | 1.089339 | 0.867788 | 1.367452 | 0.460753 |
| AC008555.1 | 0.658388 | 0.474861 | 0.912845 | 0.012179 |
| AP000844.2 | 1.130302 | 1.04069 | 1.227632 | 0.003657 |
| OCIAD1-AS1 | 1.502496 | 1.207713 | 1.869232 | 0.000259 |
| PRRT3-AS1 | 1.589616 | 1.132768 | 2.230712 | 0.007338 |
| AP001830.1 | 1.162572 | 0.95299 | 1.418246 | 0.137489 |
| AC004812.2 | 1.606224 | 1.113493 | 2.316993 | 0.011243 |
| FAM111A-DT | 0.744778 | 0.543746 | 1.020135 | 0.066392 |
| AP001011.1 | 0.966085 | 0.683421 | 1.36566 | 0.845105 |
| BX537318.1 | 1.347076 | 1.105438 | 1.641534 | 0.003139 |
| AL135999.1 | 1.429047 | 1.171349 | 1.743439 | 0.000433 |
| ALOX12-AS1 | 1.263657 | 0.861515 | 1.853512 | 0.231191 |
| AC104984.5 | 0.943417 | 0.804916 | 1.10575 | 0.472119 |
| AC138028.4 | 1.222475 | 1.050917 | 1.422038 | 0.009223 |
| AC016888.1 | 0.877381 | 0.8026 | 0.959131 | 0.004002 |
| AP003486.1 | 0.940873 | 0.687365 | 1.287878 | 0.703579 |
| AC108134.3 | 1.243922 | 1.097693 | 1.409631 | 0.000624 |
| AC004069.1 | 1.331934 | 0.974523 | 1.820426 | 0.072165 |
| AL589843.1 | 0.883402 | 0.662915 | 1.177224 | 0.397415 |
| HOXB-AS4 | 1.253157 | 1.102114 | 1.4249 | 0.000574 |
| AC234582.1 | 2.043534 | 1.566516 | 2.665807 | 1.37E-07 |
| MCF2L-AS1 | 0.976002 | 0.849554 | 1.12127 | 0.731507 |
| AC016705.2 | 0.950476 | 0.799072 | 1.130568 | 0.566144 |
| MIAT | 1.080607 | 1.028135 | 1.135756 | 0.002269 |
| AC253536.6 | 1.377797 | 1.088093 | 1.744634 | 0.007792 |
| AC096921.2 | 0.683761 | 0.462651 | 1.010543 | 0.056476 |
| AC007406.1 | 1.065192 | 0.962345 | 1.17903 | 0.222818 |
| AL138976.2 | 0.992284 | 0.652697 | 1.508552 | 0.971089 |
| AC026356.1 | 1.217026 | 0.986039 | 1.502122 | 0.067394 |
| AP002360.2 | 1.007917 | 0.939692 | 1.081096 | 0.825458 |
| AC021744.1 | 0.991461 | 0.980233 | 1.002816 | 0.139962 |
| AC008736.1 | 1.22371 | 0.977773 | 1.531508 | 0.077798 |
| ST3GAL6-AS1 | 0.793747 | 0.557505 | 1.130097 | 0.200031 |
| AC073896.3 | 1.334874 | 0.916384 | 1.944478 | 0.132329 |
| LINC02061 | 0.772863 | 0.586406 | 1.018607 | 0.067387 |
| AC010326.3 | 1.181678 | 1.118467 | 1.248461 | 2.66E-09 |
| TNFRSF10A-AS1 | 0.779926 | 0.640254 | 0.950067 | 0.01356 |
| AC012557.1 | 0.823988 | 0.450016 | 1.508738 | 0.53045 |
| ILF3-DT | 0.976721 | 0.936455 | 1.018718 | 0.272828 |
| AL139349.1 | 1.08743 | 1.039944 | 1.137084 | 0.000234 |
| AL162586.1 | 1.257493 | 1.116838 | 1.415862 | 0.000153 |
| AC004253.1 | 1.642595 | 1.295763 | 2.082262 | 4.11E-05 |
| AP003068.2 | 0.701714 | 0.541725 | 0.908954 | 0.007296 |
| U62317.1 | 1.406601 | 1.267288 | 1.561229 | 1.44E-10 |
| AL049840.4 | 1.029951 | 0.805071 | 1.317646 | 0.81436 |
| AC010976.2 | 1.042801 | 0.929572 | 1.169823 | 0.474822 |
| NR2F2-AS1 | 1.014516 | 0.712419 | 1.444716 | 0.936313 |
| LINC00460 | 1.112134 | 1.046988 | 1.181334 | 0.000559 |
| ZNF213-AS1 | 1.326633 | 1.125095 | 1.564273 | 0.000774 |
| AL096799.1 | 0.964831 | 0.91351 | 1.019035 | 0.199203 |
| AL117336.1 | 1.374128 | 1.176438 | 1.605037 | 6.06E-05 |
| AC115618.2 | 0.957583 | 0.902882 | 1.015599 | 0.148678 |
| AC004908.2 | 1.250168 | 1.019306 | 1.533319 | 0.03207 |
| AC093673.1 | 1.040032 | 1.013618 | 1.067135 | 0.002786 |
| AC009113.1 | 1.082578 | 0.849961 | 1.378858 | 0.520314 |
| LINC00571 | 0.41094 | 0.194288 | 0.869181 | 0.019976 |
| AL359091.4 | 1.620163 | 1.276983 | 2.05557 | 7.09E-05 |
| AC024337.2 | 1.189803 | 1.00882 | 1.403254 | 0.038992 |
| AL158071.3 | 1.145784 | 0.965627 | 1.359553 | 0.118946 |
| AC026367.3 | 1.373319 | 1.135707 | 1.660643 | 0.001065 |
| AC104794.3 | 1.172523 | 1.089905 | 1.261404 | 1.96E-05 |
| AC017100.1 | 0.411808 | 0.227434 | 0.745649 | 0.003402 |
| AC120498.4 | 1.027406 | 0.977523 | 1.079835 | 0.286995 |
| AC107464.2 | 1.324879 | 1.166925 | 1.504213 | 1.40E-05 |
| AC139530.1 | 1.320721 | 1.048267 | 1.663989 | 0.018282 |
| AC015802.3 | 2.181543 | 1.545534 | 3.079279 | 9.18E-06 |
| BX322562.1 | 1.025785 | 0.960192 | 1.095859 | 0.450184 |
| AC127024.4 | 1.363573 | 1.139204 | 1.632133 | 0.000723 |
| ADAMTS9-AS1 | 0.977674 | 0.956069 | 0.999766 | 0.047653 |
| AP003469.2 | 1.530837 | 1.121428 | 2.089711 | 0.007325 |
| LINC02188 | 0.870409 | 0.791101 | 0.957669 | 0.004409 |
| AC124854.1 | 0.893278 | 0.84471 | 0.944637 | 7.60E-05 |
| AC009283.1 | 1.174227 | 1.086253 | 1.269326 | 5.29E-05 |
| AL031186.1 | 1.795522 | 1.313497 | 2.454439 | 0.000243 |
| LHFPL3-AS2 | 0.93156 | 0.872021 | 0.995163 | 0.035392 |
| TOB1-AS1 | 1.400632 | 0.813416 | 2.411767 | 0.224307 |
| LINC01428 | 0.918285 | 0.819089 | 1.029494 | 0.143851 |
| AC093535.1 | 1.404058 | 1.07254 | 1.838047 | 0.013528 |
| AC012181.1 | 0.972136 | 0.797875 | 1.184457 | 0.779183 |
| U91328.1 | 0.474043 | 0.333936 | 0.672932 | 2.97E-05 |
| MIR99AHG | 0.703357 | 0.426151 | 1.160885 | 0.168688 |
| LINC00621 | 0.978618 | 0.914015 | 1.047786 | 0.535057 |
| AC004130.1 | 0.974938 | 0.75714 | 1.255387 | 0.844015 |
| AC020915.2 | 1.510873 | 1.259274 | 1.812739 | 8.97E-06 |
| LINC02391 | 1.203616 | 0.890867 | 1.626158 | 0.227348 |
| AL031673.1 | 1.525763 | 1.103418 | 2.109766 | 0.010615 |
| BCRP3 | 1.201575 | 1.064321 | 1.356528 | 0.003005 |
| AL121845.4 | 0.931445 | 0.76856 | 1.128851 | 0.468979 |
| AC034236.2 | 1.452542 | 1.188887 | 1.774667 | 0.000259 |
| AL035587.1 | 1.249616 | 0.951067 | 1.641881 | 0.109647 |
| Z97989.1 | 1.230608 | 0.8395 | 1.803926 | 0.287597 |
| LINC00174 | 1.292103 | 1.157123 | 1.442829 | 5.30E-06 |
| AC016957.2 | 2.011728 | 1.496446 | 2.70444 | 3.66E-06 |
| AF001548.2 | 1.034634 | 0.80988 | 1.321762 | 0.785259 |
| AC004264.1 | 1.091069 | 1.022485 | 1.164254 | 0.008507 |
| FBXL19-AS1 | 1.628204 | 1.201438 | 2.206562 | 0.00167 |
| SCARNA9 | 1.009018 | 0.973583 | 1.045742 | 0.622599 |
| AL118516.1 | 1.095457 | 1.018475 | 1.178258 | 0.014192 |
| CFLAR-AS1 | 0.951421 | 0.652724 | 1.386805 | 0.79561 |
| MCCC1-AS1 | 1.721945 | 1.356739 | 2.185458 | 7.88E-06 |
| ZKSCAN2-DT | 1.729772 | 1.372116 | 2.180657 | 3.54E-06 |
| EMX2OS | 0.895394 | 0.856713 | 0.935821 | 9.40E-07 |
| AL022328.3 | 1.803704 | 1.348585 | 2.412415 | 7.02E-05 |
| AL928654.1 | 0.555537 | 0.328927 | 0.938269 | 0.027931 |
| TRAF3IP2-AS1 | 1.000133 | 0.510957 | 1.957631 | 0.999691 |
| LINC02449 | 1.758347 | 1.35734 | 2.277825 | 1.93E-05 |
| AP001160.1 | 1.969001 | 1.535574 | 2.524765 | 9.24E-08 |
| AL450998.2 | 1.136683 | 0.819543 | 1.576547 | 0.442724 |
| Z95115.1 | 1.374142 | 1.201169 | 1.572024 | 3.65E-06 |
| GPRC5D-AS1 | 0.834948 | 0.539885 | 1.291273 | 0.417443 |
| AC008610.1 | 1.2087 | 1.123761 | 1.300058 | 3.42E-07 |
| DLEU2 | 1.515097 | 1.05596 | 2.17387 | 0.024098 |
| AC034231.1 | 0.912485 | 0.6134 | 1.357399 | 0.651292 |
| PHKA2-AS1 | 1.08184 | 0.837722 | 1.397097 | 0.546584 |
| AC040977.1 | 1.066833 | 1.024081 | 1.111369 | 0.001933 |
| AL590764.1 | 1.332706 | 0.949575 | 1.870423 | 0.096759 |
| RERG-IT1 | 0.975962 | 0.824223 | 1.155636 | 0.777781 |
| AC063948.1 | 1.694358 | 1.368533 | 2.097756 | 1.30E-06 |
| AC022706.1 | 1.51933 | 1.045493 | 2.207919 | 0.02829 |
| ERVK9-11 | 0.870958 | 0.764659 | 0.992035 | 0.03749 |
| LINC01355 | 1.540374 | 1.294503 | 1.832943 | 1.12E-06 |
| AL606834.1 | 1.022563 | 0.786621 | 1.329273 | 0.867602 |
| AC084824.5 | 1.296857 | 1.126125 | 1.493474 | 0.000307 |
| AC091729.3 | 1.319033 | 1.1559 | 1.505188 | 3.94E-05 |
| NCK1-DT | 1.025977 | 0.781871 | 1.346296 | 0.853235 |
| AC132872.1 | 1.16349 | 1.076486 | 1.257525 | 0.000134 |
| GLIS2-AS1 | 0.886492 | 0.710865 | 1.10551 | 0.284829 |
| AP001271.1 | 1.095691 | 0.788305 | 1.522937 | 0.586448 |
| LINC01948 | 0.751445 | 0.458732 | 1.230936 | 0.256448 |
| AC025627.1 | 0.890653 | 0.764462 | 1.037674 | 0.137399 |
| OSER1-DT | 0.968914 | 0.88482 | 1.061001 | 0.495422 |
| AC005696.1 | 0.938063 | 0.797938 | 1.102796 | 0.438591 |
| AC008982.2 | 1.116787 | 0.782455 | 1.593974 | 0.542855 |
| AL445524.1 | 1.079842 | 1.031027 | 1.130969 | 0.001136 |
| LINC00239 | 1.248642 | 0.981843 | 1.587941 | 0.07021 |
| AC139887.2 | 1.285927 | 0.918357 | 1.800616 | 0.143164 |
| AC012510.1 | 1.070541 | 0.955489 | 1.199446 | 0.239976 |
| AL662797.1 | 1.160312 | 0.936515 | 1.437591 | 0.173822 |
| DARS-AS1 | 1.009027 | 0.810357 | 1.256405 | 0.935975 |
| AP003352.1 | 1.326358 | 1.155401 | 1.522611 | 6.03E-05 |
| AP001528.2 | 0.821327 | 0.726881 | 0.928046 | 0.001588 |
| LINC01230 | 0.976931 | 0.910277 | 1.048467 | 0.517435 |
| AC009118.3 | 1.503194 | 1.060621 | 2.130444 | 0.021979 |
| AC073218.1 | 1.049858 | 1.013077 | 1.087975 | 0.007496 |
| AC009403.1 | 1.280454 | 1.102321 | 1.487373 | 0.001218 |
| AL450326.1 | 0.785793 | 0.588643 | 1.048974 | 0.101931 |
| AC084018.1 | 1.140039 | 1.057073 | 1.229515 | 0.000675 |
| AC012467.1 | 0.981617 | 0.796738 | 1.209397 | 0.861657 |
| SNHG3 | 1.15687 | 1.093566 | 1.223838 | 3.87E-07 |
| SH3BP5-AS1 | 1.228054 | 1.078116 | 1.398843 | 0.001988 |
| AC012409.1 | 0.704878 | 0.514822 | 0.965095 | 0.02914 |
| SREBF2-AS1 | 1.325251 | 0.914676 | 1.920124 | 0.13661 |
| AC022400.1 | 2.906323 | 1.537067 | 5.495343 | 0.001029 |
| LINC00265 | 1.095602 | 0.978681 | 1.226491 | 0.112808 |
| AC023024.1 | 1.024661 | 0.911652 | 1.151679 | 0.682836 |
| VIM-AS1 | 1.205817 | 0.896997 | 1.620957 | 0.215031 |
| DM1-AS | 1.677652 | 1.261393 | 2.231275 | 0.000377 |
| AC074032.1 | 1.108839 | 0.926429 | 1.327166 | 0.259899 |
| AL080248.1 | 0.794169 | 0.690027 | 0.914029 | 0.001312 |
| AP003119.3 | 1.157879 | 1.004504 | 1.334672 | 0.043182 |
| AC011477.2 | 0.783767 | 0.636277 | 0.965445 | 0.021988 |
| AC023510.2 | 1.331284 | 0.998799 | 1.774448 | 0.050966 |
| LINC00659 | 0.952623 | 0.859039 | 1.056403 | 0.357595 |
| MIR193BHG | 1.436238 | 1.254783 | 1.643934 | 1.49E-07 |
| LINC01176 | 1.083978 | 1.011708 | 1.161411 | 0.021985 |
| INE1 | 1.303836 | 1.12429 | 1.512054 | 0.000449 |
| AC022150.4 | 0.96614 | 0.696341 | 1.340475 | 0.836661 |
| LINC00173 | 1.113473 | 1.054232 | 1.176042 | 0.000117 |
| AF131215.5 | 0.949433 | 0.729475 | 1.235715 | 0.699564 |
| AC005225.2 | 0.822131 | 0.507381 | 1.332133 | 0.426404 |
| LINC01547 | 1.037068 | 0.912943 | 1.17807 | 0.575747 |
| AC104083.1 | 0.983357 | 0.958395 | 1.008969 | 0.200784 |
| AC020907.4 | 1.461041 | 1.268163 | 1.683255 | 1.53E-07 |
| AC008972.2 | 0.94543 | 0.63711 | 1.402959 | 0.780512 |
| EIF3J-DT | 0.735142 | 0.603758 | 0.895117 | 0.002192 |
| AC006435.2 | 1.491289 | 1.229999 | 1.808086 | 4.78E-05 |
| AF129075.2 | 1.59112 | 0.912128 | 2.775556 | 0.101844 |
| CASC15 | 0.824609 | 0.591319 | 1.149938 | 0.255718 |
| AC244517.7 | 0.991212 | 0.721134 | 1.362438 | 0.956626 |
| LINC01569 | 1.215183 | 1.056273 | 1.397999 | 0.006419 |
| AC116914.2 | 1.437153 | 1.265662 | 1.631881 | 2.22E-08 |
| AC007038.1 | 1.398915 | 1.201066 | 1.629356 | 1.60E-05 |
| AC012313.1 | 0.944546 | 0.702039 | 1.270822 | 0.706281 |
| HLA-F-AS1 | 1.220031 | 1.004707 | 1.481501 | 0.044711 |
| AC015819.1 | 1.363422 | 0.92106 | 2.018239 | 0.121368 |
| AC131009.1 | 1.479075 | 1.115502 | 1.961147 | 0.006541 |
| LINC01671 | 0.94269 | 0.918454 | 0.967565 | 8.94E-06 |
| AC018926.3 | 1.199208 | 0.856898 | 1.678263 | 0.289434 |
| TNFRSF14-AS1 | 1.049862 | 0.928576 | 1.186989 | 0.43724 |
| NNT-AS1 | 0.77062 | 0.637298 | 0.931834 | 0.007179 |
| COL4A2-AS1 | 1.442584 | 1.088809 | 1.911306 | 0.01069 |
| AC103691.1 | 1.146366 | 1.044874 | 1.257716 | 0.003876 |
| AC127502.2 | 1.221052 | 1.129124 | 1.320465 | 5.70E-07 |
| AF117829.1 | 1.351926 | 1.068806 | 1.710044 | 0.011904 |
| AL161669.1 | 0.840573 | 0.665145 | 1.062268 | 0.145898 |
| AC009275.1 | 1.174519 | 1.047762 | 1.31661 | 0.005768 |
| AC090517.2 | 1.894968 | 1.10226 | 3.257767 | 0.02077 |
| AC048341.2 | 1.101165 | 1.054203 | 1.150219 | 1.47E-05 |
| MMP25-AS1 | 1.295094 | 1.120024 | 1.49753 | 0.000484 |
| CHKB-DT | 1.313922 | 1.002228 | 1.722553 | 0.048147 |
| SNHG18 | 1.000523 | 0.958322 | 1.044582 | 0.981044 |
| ZNF710-AS1 | 0.988582 | 0.94513 | 1.034032 | 0.616563 |
| ADIRF-AS1 | 1.146308 | 1.01972 | 1.28861 | 0.022194 |
| AC007546.1 | 1.37146 | 1.103966 | 1.70377 | 0.004325 |
| TP53TG1 | 1.012482 | 0.992943 | 1.032406 | 0.212144 |
| AC004492.1 | 1.131484 | 0.84183 | 1.520801 | 0.412922 |
| AC108010.1 | 0.964324 | 0.806375 | 1.153211 | 0.690598 |
| AC005208.1 | 0.685017 | 0.461449 | 1.0169 | 0.060542 |
| MINCR | 1.364434 | 1.180454 | 1.577089 | 2.61E-05 |
| AC009032.1 | 0.791177 | 0.2589 | 2.417777 | 0.681094 |
| SEMA6A-AS1 | 0.911633 | 0.670759 | 1.239005 | 0.554526 |
| AC046143.2 | 1.384575 | 1.196934 | 1.601633 | 1.19E-05 |
| ARMCX5-GPRASP2 | 0.751631 | 0.453098 | 1.246859 | 0.268897 |
| AC011374.2 | 1.291781 | 1.121031 | 1.48854 | 0.000401 |
| SNHG7 | 1.016109 | 0.96826 | 1.066323 | 0.516118 |
| AC021218.1 | 0.897176 | 0.825702 | 0.974838 | 0.010419 |
| AC246817.1 | 0.724971 | 0.450942 | 1.165523 | 0.184286 |
| AC107375.1 | 1.204936 | 0.977532 | 1.485242 | 0.080635 |
| AL137003.1 | 0.794349 | 0.603541 | 1.045481 | 0.100458 |
| AC116366.1 | 1.073413 | 0.98558 | 1.169074 | 0.103845 |
| HMGA1P4 | 1.415876 | 1.211767 | 1.654366 | 1.20E-05 |
| AL121672.2 | 0.89571 | 0.487505 | 1.64572 | 0.722695 |
| AL513218.1 | 1.966909 | 1.518605 | 2.547557 | 2.97E-07 |
| MAFG-DT | 1.300126 | 1.094498 | 1.544387 | 0.002809 |
| AL603839.3 | 1.888503 | 1.189573 | 2.998086 | 0.007015 |
| AC025171.1 | 1.4688 | 1.105969 | 1.950664 | 0.007913 |
| AC026369.2 | 0.985143 | 0.938582 | 1.034014 | 0.544553 |
| SNHG9 | 1.050643 | 1.011927 | 1.090841 | 0.009911 |
| AC254562.3 | 1.20261 | 0.876925 | 1.649253 | 0.252236 |
| SNHG1 | 1.045914 | 1.021631 | 1.070774 | 0.00018 |
| AC090152.1 | 1.270275 | 0.86414 | 1.867289 | 0.223569 |
| AL589745.1 | 0.829883 | 0.672889 | 1.023506 | 0.081367 |
| AC138207.4 | 1.446181 | 1.153098 | 1.813757 | 0.001409 |
| AC103760.1 | 0.904446 | 0.723825 | 1.130138 | 0.376905 |
| AL355075.2 | 1.338856 | 1.008013 | 1.778287 | 0.043897 |
| AC009812.1 | 1.29266 | 0.974549 | 1.714608 | 0.074898 |
| WDFY3-AS2 | 0.627844 | 0.503775 | 0.782468 | 3.42E-05 |
| AC002553.2 | 1.348658 | 1.106242 | 1.644196 | 0.003089 |
| AL031717.1 | 1.340715 | 0.963376 | 1.865852 | 0.082087 |
| LINC01802 | 0.984044 | 0.948766 | 1.020633 | 0.387834 |
| AC245884.8 | 1.214385 | 1.10233 | 1.33783 | 8.41E-05 |
| MIR4435-2HG | 1.085124 | 0.996004 | 1.182218 | 0.061709 |
| HCP5 | 0.986138 | 0.967698 | 1.004929 | 0.147215 |
| AC135050.3 | 1.241273 | 1.125977 | 1.368376 | 1.39E-05 |
| AC025171.4 | 1.354444 | 1.173359 | 1.563476 | 3.42E-05 |
| AC090948.3 | 1.547497 | 1.129658 | 2.119888 | 0.006544 |
| AC004839.1 | 0.746403 | 0.584918 | 0.952469 | 0.0187 |
| AC007541.1 | 1.144546 | 0.890498 | 1.471072 | 0.291745 |
| AC145423.2 | 1.89061 | 1.494055 | 2.392418 | 1.14E-07 |
| WWC2-AS2 | 0.649816 | 0.457364 | 0.923247 | 0.016145 |
| AC008035.1 | 0.896115 | 0.700471 | 1.146403 | 0.382778 |
| AC100823.1 | 1.212872 | 0.966023 | 1.522798 | 0.096466 |
| AC111170.1 | 1.170253 | 1.044565 | 1.311066 | 0.006686 |
| AP001767.3 | 1.861527 | 1.333647 | 2.598351 | 0.00026 |
| MUC20-OT1 | 1.082968 | 1.002711 | 1.169648 | 0.042471 |
| SNHG19 | 0.999743 | 0.995053 | 1.004455 | 0.914718 |
| AC027307.2 | 1.068287 | 0.994195 | 1.1479 | 0.071667 |
| AC015912.3 | 1.778518 | 1.438945 | 2.198225 | 1.00E-07 |
| AL353593.2 | 1.120788 | 0.843715 | 1.488851 | 0.431256 |
| AC103706.1 | 1.435813 | 1.260306 | 1.635761 | 5.39E-08 |
| AL021707.3 | 1.300078 | 0.962359 | 1.756312 | 0.087274 |
| PINK1-AS | 0.682147 | 0.449099 | 1.036128 | 0.072885 |
| AC087741.1 | 1.346437 | 1.150648 | 1.575539 | 0.000207 |
| RAB11B-AS1 | 1.123154 | 0.938194 | 1.344578 | 0.205856 |
| AC109460.2 | 1.468551 | 1.083831 | 1.989832 | 0.013161 |
| AC064836.2 | 1.017744 | 0.782518 | 1.32368 | 0.895647 |
| AC026367.2 | 1.697606 | 1.269971 | 2.269239 | 0.000352 |
| AL365361.1 | 1.162468 | 0.910858 | 1.483582 | 0.226392 |
| RARA-AS1 | 1.144222 | 0.872086 | 1.501279 | 0.330924 |
| LINC00526 | 0.668238 | 0.52129 | 0.856609 | 0.001465 |
| AL118558.4 | 1.31677 | 0.888592 | 1.951271 | 0.170269 |
| AP000355.1 | 0.951962 | 0.832525 | 1.088535 | 0.471691 |
| AP001094.2 | 1.209413 | 0.808034 | 1.810171 | 0.355458 |
| AP000240.1 | 1.562661 | 1.341769 | 1.819918 | 9.42E-09 |
| AC011472.4 | 1.231403 | 0.903179 | 1.678907 | 0.188142 |
| AC093484.4 | 1.53063 | 1.149769 | 2.03765 | 0.003546 |
| AC073046.1 | 0.758624 | 0.494781 | 1.163163 | 0.205212 |
| AL355574.1 | 1.118275 | 0.895139 | 1.397034 | 0.324902 |
| AC016405.3 | 1.175337 | 1.060043 | 1.303171 | 0.002163 |
| AC092118.2 | 2.074227 | 1.458781 | 2.949325 | 4.85E-05 |
| AC090912.1 | 1.058234 | 0.661795 | 1.692155 | 0.81317 |
| AC008124.1 | 1.022282 | 0.732226 | 1.427238 | 0.897012 |
| LINC01637 | 1.244536 | 1.030316 | 1.503295 | 0.023217 |
| TBX2-AS1 | 1.03367 | 0.954768 | 1.119092 | 0.413692 |
| AC015982.1 | 1.077677 | 0.843064 | 1.37758 | 0.550384 |
| AC012467.2 | 1.098477 | 0.798297 | 1.511532 | 0.56413 |
| AC008105.3 | 1.522463 | 1.305249 | 1.775825 | 8.71E-08 |
| AC092747.4 | 0.958388 | 0.784748 | 1.170449 | 0.676863 |
| LINC01843 | 0.773986 | 0.680089 | 0.880846 | 0.000103 |
| AC110285.2 | 1.139218 | 1.059129 | 1.225364 | 0.000457 |
| EPB41L4A-DT | 0.607322 | 0.483637 | 0.762636 | 1.77E-05 |
| AC084125.2 | 1.471766 | 1.093806 | 1.980328 | 0.010708 |
| LAMA5-AS1 | 1.014768 | 0.840988 | 1.224457 | 0.878428 |
| AP002954.1 | 1.125396 | 0.887348 | 1.427305 | 0.329919 |
| AC138932.2 | 1.042129 | 1.016659 | 1.068238 | 0.001081 |
| PAN3-AS1 | 1.462634 | 0.973387 | 2.19779 | 0.06723 |
| AC016924.1 | 0.903383 | 0.763197 | 1.06932 | 0.237615 |
| AC018521.6 | 0.602943 | 0.372931 | 0.974818 | 0.039017 |
| AC009570.1 | 1.048624 | 0.874592 | 1.257287 | 0.608106 |
| AC006504.5 | 1.686626 | 0.903654 | 3.148005 | 0.100637 |
| IPO5P1 | 0.927604 | 0.726706 | 1.184038 | 0.546204 |
| AC010245.2 | 2.028229 | 1.463518 | 2.810839 | 2.16E-05 |
| AC087286.2 | 0.582493 | 0.297487 | 1.140548 | 0.11494 |
| AP001542.3 | 0.839505 | 0.78222 | 0.900986 | 1.23E-06 |
| AC011445.2 | 1.000143 | 0.935742 | 1.068975 | 0.996649 |
| AC010201.1 | 1.652379 | 1.298438 | 2.102799 | 4.44E-05 |
| AP000442.1 | 1.869467 | 1.292585 | 2.703813 | 0.00089 |
| AC135050.6 | 1.024124 | 0.988542 | 1.060986 | 0.186432 |
| THUMPD3-AS1 | 1.570421 | 1.237299 | 1.993231 | 0.000207 |
| AL606834.2 | 1.254542 | 0.856333 | 1.837924 | 0.244456 |
| PLBD1-AS1 | 0.810822 | 0.641258 | 1.025223 | 0.079797 |
| AC016747.1 | 0.989198 | 0.871905 | 1.122269 | 0.866064 |
| AL157394.1 | 1.487433 | 1.12859 | 1.960372 | 0.004821 |
| LINC00926 | 1.798959 | 1.473755 | 2.195924 | 7.83E-09 |
| AC108134.4 | 1.554838 | 1.218928 | 1.983317 | 0.000379 |
| AL133371.2 | 0.937043 | 0.788441 | 1.113654 | 0.460459 |
| AL031847.1 | 0.925305 | 0.691363 | 1.238407 | 0.601634 |
| AP002907.1 | 1.740855 | 1.302797 | 2.326209 | 0.000178 |
| AL683807.1 | 1.36818 | 1.146329 | 1.632968 | 0.000515 |
| AC011462.4 | 1.210972 | 1.115928 | 1.314111 | 4.43E-06 |
| AC104534.1 | 1.046103 | 0.99571 | 1.099045 | 0.073567 |
| AC009159.3 | 1.025452 | 0.974864 | 1.078667 | 0.3302 |
| SLC25A21-AS1 | 0.861718 | 0.634752 | 1.169838 | 0.339976 |
| RPARP-AS1 | 0.956093 | 0.799227 | 1.143746 | 0.623381 |
| ATP1A1-AS1 | 0.374418 | 0.231241 | 0.606247 | 6.46E-05 |
| AC124312.3 | 0.563067 | 0.288765 | 1.097933 | 0.091844 |
| PCAT6 | 1.106693 | 1.051376 | 1.16492 | 0.000107 |
| AC138956.1 | 1.037326 | 0.840835 | 1.279733 | 0.73234 |
| AC106820.3 | 1.0391 | 0.694109 | 1.555561 | 0.852197 |
| HOXC-AS1 | 1.055116 | 0.921942 | 1.207526 | 0.435774 |
| AC019197.1 | 1.014352 | 0.80759 | 1.27405 | 0.902485 |
| LINC00324 | 1.026521 | 0.764774 | 1.377852 | 0.861637 |
| AC005332.5 | 1.41144 | 1.176625 | 1.693117 | 0.000206 |
| AC002398.1 | 1.520234 | 1.316642 | 1.755307 | 1.13E-08 |
| AC025917.1 | 1.008312 | 0.735363 | 1.382574 | 0.959009 |
| ALG13-AS1 | 1.210425 | 0.991477 | 1.477724 | 0.060671 |
| AC015849.3 | 1.22485 | 1.043994 | 1.437035 | 0.012841 |
| AC009318.2 | 1.465314 | 1.140602 | 1.882466 | 0.002797 |
| AC007991.2 | 1.107322 | 1.011402 | 1.21234 | 0.02744 |
| DGCR9 | 1.086742 | 1.020561 | 1.157215 | 0.009464 |
| AP003419.3 | 1.409107 | 1.074181 | 1.848462 | 0.013259 |
| LINC02027 | 0.625049 | 0.497703 | 0.78498 | 5.28E-05 |
| TMEM161B-AS1 | 1.018184 | 0.825318 | 1.256118 | 0.866441 |
| AL138921.2 | 1.368195 | 0.676147 | 2.768568 | 0.383351 |
| POLR2J4 | 0.997381 | 0.669155 | 1.486602 | 0.989723 |
| PRKCZ-AS1 | 1.304767 | 0.980822 | 1.735705 | 0.067704 |
| AL021707.8 | 1.229418 | 1.067548 | 1.415832 | 0.004138 |
| AC137932.1 | 0.869672 | 0.615234 | 1.229336 | 0.429093 |
| AC009704.2 | 1.025002 | 0.875777 | 1.199655 | 0.758371 |
| AC105020.5 | 1.179979 | 0.967309 | 1.439405 | 0.102645 |
| AC093278.2 | 0.856761 | 0.792737 | 0.925956 | 9.57E-05 |
| AL133342.1 | 1.101515 | 0.762631 | 1.590985 | 0.606262 |
| AC026401.3 | 1.190951 | 1.121492 | 1.264712 | 1.20E-08 |
| LINC02298 | 0.925846 | 0.76139 | 1.125824 | 0.440005 |
| AL928654.2 | 1.144997 | 1.048392 | 1.250504 | 0.002606 |
| UBXN10-AS1 | 1.00779 | 0.911696 | 1.114013 | 0.879362 |
| JPX | 1.224713 | 1.05672 | 1.419413 | 0.007084 |
| ITPR1-DT | 1.435964 | 1.193702 | 1.727394 | 0.000124 |
| GAS6-DT | 0.864534 | 0.672963 | 1.110637 | 0.25473 |
| AC005586.2 | 1.233466 | 1.10244 | 1.380065 | 0.00025 |
| AC009237.15 | 1.151483 | 0.803951 | 1.649246 | 0.441599 |
| AC037459.2 | 1.242976 | 0.854108 | 1.808891 | 0.255873 |
| SNHG26 | 1.516244 | 1.164809 | 1.97371 | 0.001975 |
| AC027601.3 | 0.59943 | 0.379476 | 0.946874 | 0.028236 |
| THAP9-AS1 | 1.024725 | 0.978892 | 1.072703 | 0.295485 |
| AC093157.1 | 1.785756 | 1.327532 | 2.402144 | 0.000127 |
| ENTPD3-AS1 | 0.762834 | 0.481953 | 1.207412 | 0.247893 |
| AC073316.2 | 0.975997 | 0.779391 | 1.222198 | 0.832349 |
| SNHG20 | 1.150097 | 1.020686 | 1.295915 | 0.021667 |
| ZNF674-AS1 | 1.115559 | 0.941389 | 1.321954 | 0.206731 |
| AC009812.4 | 1.348428 | 1.059249 | 1.716556 | 0.01521 |
| LINC01560 | 0.916139 | 0.70202 | 1.195566 | 0.519016 |
| CIRBP-AS1 | 1.413627 | 0.987121 | 2.024413 | 0.058863 |
| AL604028.1 | 1.005986 | 0.804779 | 1.257498 | 0.958195 |
| OTUD6B-AS1 | 0.736644 | 0.621058 | 0.873742 | 0.000448 |
| LINC00944 | 1.489247 | 1.249004 | 1.775699 | 9.12E-06 |
| AC016813.1 | 0.883705 | 0.75242 | 1.037897 | 0.131897 |
| AC046143.1 | 1.776494 | 1.360613 | 2.319492 | 2.41E-05 |
| LINC00494 | 1.120955 | 0.946908 | 1.326993 | 0.184745 |
| LINC02062 | 1.688402 | 1.267986 | 2.248211 | 0.000337 |
| BAIAP2-DT | 0.907893 | 0.834393 | 0.987869 | 0.024876 |
| AC005911.1 | 2.058334 | 1.266542 | 3.345121 | 0.003572 |
| AC003070.1 | 1.256073 | 1.056937 | 1.492728 | 0.009633 |
| AP005233.2 | 1.024127 | 1.006971 | 1.041576 | 0.005677 |
| HOXB-AS1 | 1.280895 | 1.139226 | 1.440181 | 3.48E-05 |
| ADAMTS9-AS2 | 0.650048 | 0.471825 | 0.895592 | 0.008428 |
| AL117335.1 | 1.072932 | 0.959282 | 1.200046 | 0.217848 |
| LINC00909 | 0.745861 | 0.593326 | 0.93761 | 0.012011 |
| AL021878.2 | 1.184764 | 0.835677 | 1.679674 | 0.3411 |
| AP006623.1 | 1.114585 | 0.975309 | 1.273751 | 0.11119 |
| AC080013.4 | 1.050708 | 0.923174 | 1.195861 | 0.453731 |
| MIR503HG | 1.057163 | 1.014779 | 1.101318 | 0.007753 |
| AL583856.2 | 1.545403 | 0.90008 | 2.653399 | 0.114504 |
| LINC01534 | 0.793516 | 0.532256 | 1.183018 | 0.256333 |
| GAS6-AS1 | 1.044118 | 0.955557 | 1.140887 | 0.339742 |
| AC100814.1 | 1.105951 | 1.027508 | 1.190382 | 0.007298 |
| AC067852.3 | 1.072996 | 0.705005 | 1.633068 | 0.742323 |
| CCDC183-AS1 | 0.90977 | 0.77109 | 1.073392 | 0.262438 |
| AC008969.1 | 1.095595 | 0.774529 | 1.549753 | 0.605871 |
| SCAMP1-AS1 | 0.943856 | 0.841191 | 1.059051 | 0.32538 |
| LINC02482 | 0.943676 | 0.818168 | 1.088437 | 0.42594 |
| AC138696.2 | 1.148665 | 0.985153 | 1.339316 | 0.076888 |
| FAM13A-AS1 | 1.333292 | 1.119357 | 1.588116 | 0.001266 |
| AGBL5-IT1 | 1.372804 | 1.074325 | 1.75421 | 0.011305 |
| AC139887.4 | 1.204643 | 0.853812 | 1.699629 | 0.289104 |
| C1RL-AS1 | 1.040554 | 0.969602 | 1.116698 | 0.269914 |
| AP001625.2 | 0.686807 | 0.478491 | 0.985815 | 0.041606 |
| AC027458.1 | 0.897897 | 0.780306 | 1.033209 | 0.13263 |
| AC121338.2 | 0.195919 | 0.107972 | 0.355501 | 8.23E-08 |
| HLA-DQB1-AS1 | 1.022314 | 0.982464 | 1.063781 | 0.276649 |
| AC116345.3 | 0.953302 | 0.891163 | 1.019773 | 0.164342 |
| BX322234.1 | 1.506071 | 1.182837 | 1.917637 | 0.000893 |
| LINC00342 | 1.1715 | 1.098433 | 1.249427 | 1.46E-06 |
| MZF1-AS1 | 1.969857 | 1.343381 | 2.888485 | 0.000518 |
| AL513320.1 | 1.401956 | 1.205934 | 1.629841 | 1.10E-05 |
| AL133415.1 | 1.070589 | 0.837546 | 1.368475 | 0.586043 |
| LNCTAM34A | 0.997758 | 0.753888 | 1.320515 | 0.987475 |
| AC063919.1 | 0.767483 | 0.628286 | 0.93752 | 0.009547 |
| AC010973.2 | 1.581041 | 1.350285 | 1.851233 | 1.26E-08 |
| SBF2-AS1 | 0.904697 | 0.772212 | 1.059912 | 0.215075 |
| AC138956.2 | 0.964252 | 0.839064 | 1.108119 | 0.607921 |
| OVOL1-AS1 | 0.982427 | 0.8915 | 1.082629 | 0.72051 |
| AC036176.1 | 1.010954 | 0.76095 | 1.343095 | 0.940086 |
| AC015871.3 | 1.100606 | 0.957398 | 1.265235 | 0.177711 |
| LINC01578 | 1.03406 | 0.954746 | 1.119962 | 0.410746 |
| AL645939.4 | 0.663754 | 0.4569 | 0.964257 | 0.031477 |
| AC010655.2 | 1.028899 | 0.950165 | 1.114157 | 0.483055 |
| AP000894.4 | 0.921796 | 0.691116 | 1.229473 | 0.579482 |
| AC009974.1 | 1.265982 | 0.890929 | 1.798921 | 0.188278 |
| AL731567.1 | 1.481746 | 1.283564 | 1.710529 | 7.97E-08 |
| AC104564.3 | 1.562692 | 1.195413 | 2.042814 | 0.001092 |
| SNHG15 | 1.078543 | 1.03436 | 1.124614 | 0.000396 |
| ERVK13-1 | 1.249129 | 0.950533 | 1.641525 | 0.110494 |
| MIATNB | 1.174228 | 0.895596 | 1.539548 | 0.245186 |
| AC130469.1 | 1.926373 | 1.444308 | 2.569336 | 8.13E-06 |
| AC125807.2 | 1.023268 | 0.788955 | 1.327168 | 0.86237 |
| BACE1-AS | 1.227196 | 1.108259 | 1.358897 | 8.28E-05 |
| AC009159.2 | 1.136278 | 0.910216 | 1.418484 | 0.258986 |
| LINC00958 | 1.015889 | 0.991912 | 1.040446 | 0.195812 |
| AL050341.2 | 1.130516 | 0.97832 | 1.306388 | 0.09634 |
| KMT2E-AS1 | 1.096268 | 1.050904 | 1.143589 | 2.02E-05 |
| AP001189.3 | 0.902766 | 0.829252 | 0.982798 | 0.018258 |
| LINC00623 | 1.46775 | 1.20074 | 1.794135 | 0.00018 |
| AC116651.1 | 0.734613 | 0.420684 | 1.282808 | 0.278217 |
| LINC01003 | 0.971335 | 0.731372 | 1.290031 | 0.840784 |
| NR2F1-AS1 | 0.679922 | 0.427851 | 1.080502 | 0.102606 |
| AC006942.1 | 1.324113 | 1.07505 | 1.630878 | 0.008275 |
| AL135999.3 | 0.922594 | 0.786503 | 1.082233 | 0.322449 |
| AL512791.1 | 1.097955 | 0.977872 | 1.232784 | 0.113806 |
| HCG18 | 0.923431 | 0.613228 | 1.390551 | 0.702906 |
| AC098484.1 | 0.512316 | 0.372755 | 0.704131 | 3.76E-05 |
| AC136475.3 | 1.007891 | 1.000119 | 1.015723 | 0.046578 |
| AC023794.4 | 1.014606 | 0.786007 | 1.309691 | 0.911357 |
| AC009090.1 | 1.085981 | 0.945707 | 1.247063 | 0.242448 |
| IQCH-AS1 | 0.793662 | 0.474642 | 1.327103 | 0.378293 |
| AC005785.1 | 2.33808 | 1.760044 | 3.105955 | 4.58E-09 |
| SNHG14 | 0.81395 | 0.582892 | 1.1366 | 0.226907 |
| AC234775.3 | 0.486949 | 0.27354 | 0.866855 | 0.014463 |
| AC025287.3 | 1.761793 | 1.141072 | 2.720174 | 0.010605 |
| AL844908.1 | 1.046281 | 0.989144 | 1.106719 | 0.114332 |
| LBX2-AS1 | 1.027412 | 0.967224 | 1.091344 | 0.379942 |
| ZNF460-AS1 | 1.697616 | 1.28395 | 2.244557 | 0.000204 |
| HMGN3-AS1 | 1.030424 | 0.738596 | 1.437556 | 0.859971 |
| CR936218.1 | 1.333142 | 1.102607 | 1.611877 | 0.002994 |
| AC012615.6 | 1.542816 | 1.206043 | 1.973628 | 0.000559 |
| AL731571.1 | 1.241525 | 0.851511 | 1.810177 | 0.260813 |
| AP000766.1 | 0.507326 | 0.260043 | 0.989756 | 0.046573 |
| MALAT1 | 1.000202 | 0.998777 | 1.001629 | 0.781212 |
| NUP50-DT | 1.52962 | 1.276754 | 1.832567 | 4.03E-06 |
| LINC00501 | 0.873028 | 0.561371 | 1.357707 | 0.546714 |
| AC135803.1 | 1.017865 | 0.942387 | 1.099387 | 0.652391 |
| MAN1B1-DT | 0.598369 | 0.43689 | 0.819532 | 0.001373 |
| AC002553.1 | 1.391374 | 1.167158 | 1.658662 | 0.00023 |
| RAD51-AS1 | 1.1394 | 1.044543 | 1.242872 | 0.003255 |
| NAPA-AS1 | 0.638713 | 0.430523 | 0.947579 | 0.025913 |
| AC068338.3 | 0.681086 | 0.419622 | 1.105467 | 0.120134 |
| AC090948.1 | 1.067338 | 0.819879 | 1.389487 | 0.628213 |
| AC096992.2 | 1.18563 | 0.790298 | 1.778721 | 0.410639 |
| AL133338.1 | 1.358737 | 1.021756 | 1.806855 | 0.035034 |
| TGFB2-AS1 | 0.637156 | 0.372459 | 1.089968 | 0.099872 |
| AC026471.1 | 0.75492 | 0.531472 | 1.072311 | 0.116399 |
| BNC2-AS1 | 1.040289 | 0.97163 | 1.113799 | 0.256868 |
| AC006064.3 | 1.240195 | 1.021073 | 1.506339 | 0.029991 |
| AL138826.1 | 0.947925 | 0.907242 | 0.990432 | 0.01687 |
| AC010761.3 | 0.823749 | 0.516671 | 1.313335 | 0.415254 |
| AC010136.1 | 1.003055 | 0.839608 | 1.19832 | 0.973187 |
| AC139795.2 | 1.129834 | 0.803581 | 1.588546 | 0.482589 |
| AC087752.3 | 1.177669 | 0.997341 | 1.390603 | 0.053785 |
| SLC9A3-AS1 | 0.996382 | 0.985646 | 1.007235 | 0.512013 |
| AC006449.2 | 0.851543 | 0.52684 | 1.376368 | 0.51183 |
| AC025171.2 | 1.40453 | 1.04609 | 1.88579 | 0.02384 |
| AC022079.2 | 1.484757 | 1.085786 | 2.030329 | 0.013308 |
| AL158166.1 | 1.681449 | 0.985453 | 2.869006 | 0.056623 |
| LINC00513 | 1.032253 | 0.888447 | 1.199335 | 0.678353 |
| ADNP-AS1 | 0.920451 | 0.682123 | 1.242047 | 0.587695 |
| AP000866.2 | 0.838193 | 0.632562 | 1.11067 | 0.219046 |
| ZEB1-AS1 | 0.952924 | 0.73357 | 1.237869 | 0.717903 |
| SNHG12 | 1.044715 | 1.017803 | 1.072339 | 0.001019 |
| AL596094.1 | 1.720867 | 1.147007 | 2.581837 | 0.008726 |
| URB1-AS1 | 1.123322 | 1.015712 | 1.242334 | 0.023612 |
| TMEM9B-AS1 | 1.088973 | 0.88625 | 1.338068 | 0.417369 |
| NORAD | 0.984364 | 0.972907 | 0.995957 | 0.008336 |
| AC008014.1 | 0.523335 | 0.292323 | 0.936907 | 0.029309 |
| AL662844.3 | 1.11146 | 0.908518 | 1.359734 | 0.304281 |
| AC108488.1 | 1.421172 | 1.205384 | 1.675589 | 2.88E-05 |
| AC092794.1 | 1.706626 | 1.229115 | 2.36965 | 0.001414 |
| AC069281.2 | 2.31412 | 1.722222 | 3.109442 | 2.60E-08 |
| AC025857.2 | 1.162816 | 1.092828 | 1.237286 | 1.91E-06 |
| AL136531.1 | 1.68896 | 1.142684 | 2.496393 | 0.008563 |
| AC027796.4 | 1.364282 | 1.204178 | 1.545672 | 1.08E-06 |
| AL353803.1 | 1.006677 | 0.925033 | 1.095527 | 0.877446 |
| AC009133.1 | 1.212768 | 1.083943 | 1.356905 | 0.000761 |
| AC104958.2 | 0.995246 | 0.953245 | 1.039097 | 0.828499 |
| HNF1A-AS1 | 1.039268 | 0.945178 | 1.142725 | 0.426327 |
| AL122010.1 | 1.003724 | 0.831739 | 1.211271 | 0.969081 |
| AC011498.6 | 1.799214 | 1.282846 | 2.523429 | 0.000666 |
| AL359091.3 | 1.746215 | 1.192483 | 2.557075 | 0.004176 |
| AC008443.5 | 0.845796 | 0.704748 | 1.015073 | 0.071981 |
| CRNDE | 1.043126 | 0.990071 | 1.099025 | 0.1129 |
| AC068792.1 | 2.069182 | 1.400106 | 3.057992 | 0.000264 |
| DICER1-AS1 | 1.482296 | 1.222012 | 1.798019 | 6.47E-05 |
| AC083964.1 | 0.803895 | 0.646749 | 0.999223 | 0.049187 |
| AC093110.1 | 1.191119 | 0.983018 | 1.443274 | 0.074238 |
| TMEM147-AS1 | 1.410219 | 1.196709 | 1.661822 | 4.06E-05 |
| AC008770.3 | 0.913396 | 0.593983 | 1.404573 | 0.679907 |
| AC008870.2 | 2.655195 | 1.847799 | 3.815384 | 1.30E-07 |
| AC018690.1 | 2.52253 | 1.643733 | 3.871161 | 2.29E-05 |
| AC004908.3 | 1.719314 | 1.438987 | 2.054251 | 2.41E-09 |
| AL137247.1 | 1.194762 | 1.06436 | 1.341141 | 0.002547 |
| AL138756.1 | 1.057116 | 0.84437 | 1.323464 | 0.628053 |
| AC005332.3 | 1.13036 | 1.034338 | 1.235297 | 0.006823 |
| AC093726.1 | 0.948468 | 0.809969 | 1.11065 | 0.511236 |
| IGFL2-AS1 | 1.006683 | 0.999949 | 1.013463 | 0.051757 |
| AC018816.1 | 1.06781 | 0.959085 | 1.18886 | 0.231118 |
| AC027644.3 | 1.053192 | 0.933738 | 1.187927 | 0.398804 |
| Z69706.1 | 1.379628 | 1.113085 | 1.709998 | 0.003302 |
| AC025175.1 | 1.093299 | 0.879509 | 1.359056 | 0.421702 |
| AL357033.3 | 0.891983 | 0.646834 | 1.230043 | 0.485698 |
| AC090198.1 | 0.818078 | 0.580146 | 1.153593 | 0.252158 |
| AC004918.1 | 1.020487 | 0.953752 | 1.091891 | 0.556724 |
| AC009120.2 | 1.341045 | 1.180434 | 1.523509 | 6.53E-06 |
| AC015819.2 | 1.032994 | 0.964229 | 1.106662 | 0.355709 |
| LINC02532 | 0.984854 | 0.963566 | 1.006613 | 0.171052 |
| AL161669.3 | 1.101103 | 1.034162 | 1.172377 | 0.002615 |
| NIFK-AS1 | 0.907602 | 0.674741 | 1.220827 | 0.521578 |
| AC004477.3 | 2.083833 | 1.191401 | 3.64475 | 0.010055 |
| AC067838.1 | 1.419781 | 1.125576 | 1.790887 | 0.003092 |
| DLGAP1-AS2 | 1.484856 | 1.312576 | 1.679748 | 3.33E-10 |
| AL139246.5 | 0.964763 | 0.800573 | 1.162628 | 0.706262 |
| AC084782.3 | 1.072152 | 0.693281 | 1.658072 | 0.754137 |
| AL031429.2 | 0.906584 | 0.811687 | 1.012576 | 0.082136 |
| AC020558.2 | 1.763474 | 1.280049 | 2.429471 | 0.00052 |
| AC103591.3 | 1.170532 | 1.030326 | 1.329818 | 0.015567 |
| AC093726.2 | 1.61774 | 1.320157 | 1.982402 | 3.52E-06 |
| LINC00278 | 0.826875 | 0.684681 | 0.998599 | 0.048322 |
| AC116407.2 | 1.328828 | 1.181145 | 1.494975 | 2.25E-06 |
| AC018742.1 | 0.823222 | 0.72679 | 0.93245 | 0.002212 |
| ARHGAP27P1-BPTFP1-KPNA2P3 | 1.311734 | 1.120926 | 1.535023 | 0.000716 |
| AC133552.5 | 0.94034 | 0.803378 | 1.100653 | 0.443741 |
| ARRDC1-AS1 | 1.210026 | 1.093194 | 1.339343 | 0.000233 |
| AC073346.1 | 0.687754 | 0.562142 | 0.841436 | 0.000275 |
| STAG3L5P-PVRIG2P-PILRB | 1.342322 | 1.142919 | 1.576514 | 0.000333 |
| AC125437.1 | 2.173635 | 1.416687 | 3.335028 | 0.000378 |
| AC253536.3 | 0.865775 | 0.632007 | 1.186009 | 0.36941 |
| AL162424.1 | 1.04541 | 0.91902 | 1.189183 | 0.499366 |
| PRR34-AS1 | 0.990641 | 0.925143 | 1.060776 | 0.787606 |
| AC010776.2 | 0.971838 | 0.90849 | 1.039604 | 0.406193 |
| AL360181.1 | 1.249763 | 0.995813 | 1.568474 | 0.054384 |
| AL645568.1 | 2.71234 | 1.511812 | 4.866206 | 0.00082 |
| TNRC6C-AS1 | 1.41946 | 1.123718 | 1.793038 | 0.003298 |
| PARD3-AS1 | 1.267494 | 1.100563 | 1.459745 | 0.001002 |
| AL049869.3 | 0.956221 | 0.602877 | 1.516659 | 0.849144 |
| N4BP2L2-IT2 | 1.300752 | 1.071798 | 1.578615 | 0.00777 |
| LINC01615 | 1.071732 | 1.031432 | 1.113607 | 0.000396 |
| AL390719.2 | 1.013351 | 0.94115 | 1.091092 | 0.725074 |
| MIR200CHG | 0.944505 | 0.78441 | 1.137274 | 0.546837 |
| PVT1 | 1.16116 | 1.076105 | 1.252937 | 0.000118 |
| AC010201.2 | 1.358682 | 1.104169 | 1.671861 | 0.003776 |
| AC145098.1 | 1.353367 | 1.069827 | 1.712055 | 0.011647 |
| LINC01150 | 1.107254 | 0.917354 | 1.336464 | 0.288532 |
| AC018730.1 | 0.954586 | 0.887796 | 1.0264 | 0.209167 |
| AL121992.3 | 0.852524 | 0.706137 | 1.029258 | 0.096927 |
| AC092287.1 | 1.103643 | 0.68757 | 1.771497 | 0.682939 |
| RUSC1-AS1 | 1.352534 | 1.160087 | 1.576906 | 0.000115 |
| HOXA-AS2 | 1.390297 | 1.188556 | 1.626281 | 3.80E-05 |
| DNAJC3-DT | 0.976831 | 0.737048 | 1.294621 | 0.870421 |
| OIP5-AS1 | 0.897977 | 0.831208 | 0.97011 | 0.006338 |
| LINC01697 | 0.798321 | 0.694371 | 0.917832 | 0.001553 |
| AC018653.3 | 1.33744 | 1.182387 | 1.512825 | 3.75E-06 |
| AC007637.1 | 0.699092 | 0.488176 | 1.001133 | 0.050727 |
| AL662791.1 | 1.071165 | 0.791714 | 1.449255 | 0.655801 |
| AC004847.1 | 1.11268 | 1.033622 | 1.197786 | 0.004521 |
| AL031670.1 | 1.674829 | 1.255356 | 2.234467 | 0.000455 |
| AP004608.1 | 0.958962 | 0.841979 | 1.092198 | 0.527844 |
| AC060766.4 | 1.092168 | 0.980454 | 1.216609 | 0.109284 |
| AL031710.1 | 0.990425 | 0.944428 | 1.038662 | 0.691698 |
| AL136295.6 | 1.216289 | 1.034209 | 1.430424 | 0.017957 |
| AL022316.1 | 1.949301 | 1.302071 | 2.918256 | 0.001187 |
| LINC01004 | 1.25663 | 1.140236 | 1.384905 | 4.10E-06 |
| LINC01320 | 0.993466 | 0.978651 | 1.008505 | 0.392483 |
| MEG3 | 1.426232 | 1.194539 | 1.702863 | 8.66E-05 |
| HOXD-AS2 | 1.179242 | 1.035309 | 1.343185 | 0.013049 |
| AC068870.2 | 0.906748 | 0.820095 | 1.002557 | 0.056116 |
| LINC01943 | 1.587599 | 1.298269 | 1.941407 | 6.70E-06 |
| AC012640.4 | 1.041086 | 0.718129 | 1.509281 | 0.831717 |
| AL365330.1 | 1.221805 | 1.001711 | 1.490257 | 0.048061 |
| CCDC18-AS1 | 1.216422 | 1.137629 | 1.300672 | 9.82E-09 |
| LINC00472 | 0.826668 | 0.626486 | 1.090814 | 0.178455 |
| AC108673.3 | 1.238511 | 1.141705 | 1.343526 | 2.59E-07 |
| STPG3-AS1 | 1.032248 | 0.896278 | 1.188847 | 0.659627 |
| AF186192.1 | 0.970974 | 0.677902 | 1.390747 | 0.872346 |
| AC091185.1 | 1.243194 | 0.935777 | 1.651601 | 0.133105 |
| CARD8-AS1 | 0.898342 | 0.731813 | 1.102766 | 0.305444 |
| AC026347.1 | 0.948501 | 0.792201 | 1.135639 | 0.564956 |
| AC018638.7 | 1.416106 | 1.13613 | 1.765076 | 0.001965 |
| LINC00106 | 1.072843 | 1.010323 | 1.139233 | 0.021722 |
| BX255925.1 | 1.077474 | 0.795837 | 1.45878 | 0.629301 |
| LINC00667 | 0.936869 | 0.816153 | 1.075439 | 0.354146 |
| FOXN3-AS1 | 0.959551 | 0.866523 | 1.062567 | 0.427445 |
| NDUFV2-AS1 | 1.736304 | 1.221783 | 2.467501 | 0.00209 |
| AC008946.1 | 1.118564 | 0.774297 | 1.615898 | 0.550503 |
| AC100803.3 | 0.935828 | 0.779411 | 1.123636 | 0.477237 |
| AC147067.2 | 1.117487 | 0.99799 | 1.251291 | 0.054217 |
| ARHGAP31-AS1 | 0.454975 | 0.243889 | 0.848753 | 0.013307 |
| AC002550.2 | 1.484586 | 0.980455 | 2.247933 | 0.061942 |
| LINC00893 | 1.452931 | 1.192585 | 1.770113 | 0.000209 |
| AC008735.2 | 1.100422 | 1.045609 | 1.158108 | 0.000242 |
| AP005899.1 | 1.298609 | 1.019468 | 1.654182 | 0.034335 |
| AL451165.2 | 1.302712 | 1.01488 | 1.672177 | 0.037902 |
| GSEC | 1.371817 | 1.060119 | 1.775161 | 0.016221 |
| AL354836.1 | 1.066457 | 1.031106 | 1.103019 | 0.000183 |
| CCNT2-AS1 | 0.92831 | 0.585922 | 1.470776 | 0.75137 |
| AC010883.1 | 1.194699 | 1.06371 | 1.341818 | 0.002679 |
| SNHG10 | 1.580878 | 1.274744 | 1.960532 | 3.04E-05 |
| AC124312.2 | 0.754309 | 0.57157 | 0.995472 | 0.046368 |
| DLGAP1-AS1 | 1.125095 | 1.055401 | 1.199391 | 0.000303 |
| AC147067.1 | 1.276307 | 1.104126 | 1.475337 | 0.000968 |
| AL359962.2 | 1.279364 | 0.863169 | 1.896237 | 0.219796 |
| LY6E-DT | 0.798925 | 0.629282 | 1.014301 | 0.065276 |
| Z98884.2 | 1.313587 | 1.017208 | 1.696321 | 0.036551 |
| PSORS1C3 | 0.997398 | 0.984761 | 1.010198 | 0.688828 |
| AL049838.1 | 1.105171 | 0.947461 | 1.289134 | 0.203034 |
| HCG15 | 0.831434 | 0.535237 | 1.291545 | 0.411371 |
| MIR222HG | 1.008141 | 0.934226 | 1.087903 | 0.834687 |
| SNHG17 | 1.179725 | 1.111549 | 1.252083 | 5.27E-08 |
| AC037198.2 | 1.452122 | 1.174966 | 1.794655 | 0.000556 |
| AC254633.1 | 0.843823 | 0.721352 | 0.987088 | 0.033804 |
| AC108704.1 | 1.225344 | 0.974977 | 1.540003 | 0.081393 |
| CNNM3-DT | 0.915796 | 0.777643 | 1.078492 | 0.291754 |
| AC002401.4 | 1.026054 | 0.952123 | 1.105726 | 0.500243 |
| AC024075.1 | 0.919743 | 0.777246 | 1.088364 | 0.330017 |
| AC022509.3 | 0.969295 | 0.813809 | 1.154487 | 0.726641 |
| AL139246.3 | 1.122266 | 0.838658 | 1.50178 | 0.437686 |
| FOXD2-AS1 | 1.504391 | 1.305768 | 1.733227 | 1.58E-08 |
| AP000254.1 | 1.271338 | 1.014816 | 1.592703 | 0.036809 |
| AL513550.1 | 1.136658 | 0.852354 | 1.515791 | 0.383104 |
| ACVR2B-AS1 | 0.345785 | 0.192724 | 0.620407 | 0.00037 |
| AC010834.3 | 1.071039 | 0.799681 | 1.434478 | 0.645243 |
| AC022306.2 | 1.223874 | 1.049363 | 1.427406 | 0.010058 |
| DGCR10 | 1.12424 | 1.03369 | 1.222722 | 0.006269 |
| AC242842.1 | 1.127788 | 0.938148 | 1.355763 | 0.200456 |
| AC005674.2 | 1.1821 | 0.915095 | 1.527012 | 0.200295 |
| AC060780.1 | 1.143834 | 1.006378 | 1.300064 | 0.039658 |
| AL450992.1 | 0.798269 | 0.526304 | 1.210768 | 0.2891 |
| AL022322.1 | 1.217217 | 1.07541 | 1.377723 | 0.001869 |
| AL158212.3 | 0.808834 | 0.552189 | 1.18476 | 0.275974 |
| AFDN-DT | 1.055644 | 0.791553 | 1.407846 | 0.712398 |
| AC096677.1 | 1.316168 | 1.050381 | 1.649209 | 0.016984 |
| AC129510.1 | 1.651829 | 1.352634 | 2.017205 | 8.54E-07 |
| AL645608.7 | 1.094654 | 0.986134 | 1.215117 | 0.089539 |
| LINC00886 | 0.59608 | 0.396374 | 0.896405 | 0.012944 |
| SEMA3B-AS1 | 0.988111 | 0.890819 | 1.096029 | 0.821078 |
| AC012368.1 | 1.155958 | 1.034192 | 1.292061 | 0.010712 |
| MIR4458HG | 0.874015 | 0.72626 | 1.05183 | 0.15411 |
| GATA2-AS1 | 0.802042 | 0.597338 | 1.076896 | 0.142315 |
| AP000873.2 | 1.583399 | 1.000328 | 2.50633 | 0.049836 |
| AC068580.1 | 1.161167 | 1.049639 | 1.284545 | 0.003728 |
| AL133243.2 | 1.222208 | 0.913986 | 1.63437 | 0.175941 |
| ZNF32-AS2 | 1.576635 | 1.198821 | 2.073518 | 0.001125 |
| AC010618.3 | 2.320909 | 1.505405 | 3.578187 | 0.000138 |
| AC020659.1 | 1.056097 | 0.900276 | 1.238889 | 0.502777 |
| AC073611.1 | 1.291072 | 1.184966 | 1.40668 | 5.26E-09 |
| SNHG25 | 1.054727 | 0.991678 | 1.121784 | 0.090219 |
| AL031775.1 | 1.531713 | 0.997137 | 2.352881 | 0.05155 |
| AC002091.1 | 0.89869 | 0.583856 | 1.383292 | 0.62737 |
| BX470102.1 | 1.06994 | 0.960798 | 1.191479 | 0.218146 |
| AC015813.1 | 1.280618 | 1.129941 | 1.451387 | 0.000108 |
| AL133355.1 | 0.50557 | 0.370266 | 0.690317 | 1.77E-05 |
| AC139768.1 | 0.881782 | 0.664444 | 1.17021 | 0.383569 |
| SNHG6 | 1.012704 | 1.005462 | 1.019997 | 0.000566 |
| LMO7-AS1 | 0.902856 | 0.694973 | 1.172923 | 0.444047 |
| AC090114.2 | 0.993651 | 0.601642 | 1.64108 | 0.98015 |
| AL109811.2 | 1.175228 | 1.072993 | 1.287205 | 0.000507 |
| CYTOR | 1.044967 | 1.009254 | 1.081943 | 0.013169 |
| AC018752.1 | 0.529101 | 0.392201 | 0.713786 | 3.08E-05 |
| GK-AS1 | 1.159076 | 0.952953 | 1.409784 | 0.13951 |
| AC012181.2 | 0.897729 | 0.734849 | 1.096712 | 0.290877 |
| AL117336.2 | 1.33304 | 1.148242 | 1.54758 | 0.00016 |
| AC036214.2 | 0.847776 | 0.437201 | 1.643921 | 0.625014 |
| AL031123.1 | 0.960304 | 0.869512 | 1.060576 | 0.42409 |
| AP000759.1 | 0.877222 | 0.726515 | 1.059191 | 0.173185 |
| AC023043.1 | 1.203612 | 1.123752 | 1.289147 | 1.22E-07 |
| AC010737.1 | 0.780323 | 0.633946 | 0.960498 | 0.019273 |
| AL359715.3 | 0.500947 | 0.216451 | 1.159375 | 0.106406 |
| AC010761.1 | 1.394344 | 1.159936 | 1.676123 | 0.0004 |
| LINC02361 | 1.170493 | 0.932872 | 1.468642 | 0.173905 |
| ATP1B3-AS1 | 1.094131 | 0.892481 | 1.341342 | 0.386743 |
| AL078581.2 | 0.581643 | 0.398705 | 0.84852 | 0.004916 |
| FTX | 0.92433 | 0.625803 | 1.365264 | 0.692542 |
| AC005840.2 | 1.352752 | 1.135953 | 1.610929 | 0.000698 |
| AC124944.1 | 1.057273 | 0.991716 | 1.127164 | 0.088145 |
| VPS9D1-AS1 | 1.863296 | 1.473372 | 2.356413 | 2.05E-07 |
| AP000345.2 | 0.988541 | 0.841654 | 1.161064 | 0.888325 |
| AC012442.1 | 1.053013 | 0.977957 | 1.13383 | 0.17095 |
| AL359881.1 | 1.242134 | 1.113973 | 1.38504 | 9.52E-05 |
| LINC02041 | 0.910813 | 0.848356 | 0.977868 | 0.009953 |
| AL355488.1 | 1.264451 | 1.137364 | 1.405738 | 1.41E-05 |
| AL118558.3 | 0.987388 | 0.807156 | 1.207864 | 0.901767 |
| SNHG11 | 1.243455 | 1.101566 | 1.40362 | 0.000424 |
| AC003102.1 | 1.59736 | 1.298439 | 1.965098 | 9.40E-06 |
| AC007566.1 | 1.15253 | 1.013329 | 1.310855 | 0.030651 |
| AC015726.1 | 1.158916 | 0.976804 | 1.374981 | 0.090858 |
| AC006213.4 | 0.859402 | 0.515959 | 1.431455 | 0.56053 |
| MMP2-AS1 | 1.122658 | 1.022036 | 1.233187 | 0.015739 |
| LINC01436 | 1.047127 | 1.021316 | 1.07359 | 0.000299 |
| SLC25A25-AS1 | 1.101455 | 0.989438 | 1.226154 | 0.077409 |
| AP006621.3 | 1.145065 | 1.060143 | 1.23679 | 0.00057 |
| AC087276.2 | 1.227037 | 1.017332 | 1.479971 | 0.032382 |
| AC091849.2 | 1.17017 | 0.92034 | 1.487817 | 0.199668 |
| AC019080.5 | 1.123412 | 0.772073 | 1.634632 | 0.543091 |
| AL162171.1 | 0.448938 | 0.253994 | 0.793504 | 0.005853 |
| AC078883.1 | 0.907175 | 0.649745 | 1.266601 | 0.56726 |
| AC123912.1 | 0.887413 | 0.602829 | 1.306344 | 0.544891 |
| AC132192.2 | 1.762222 | 1.432732 | 2.167485 | 8.10E-08 |
| LINC00957 | 0.860261 | 0.714625 | 1.035578 | 0.111713 |
| AC025181.2 | 1.079259 | 0.895467 | 1.300775 | 0.42325 |
| AC112496.1 | 1.319163 | 1.055563 | 1.648591 | 0.014876 |
| LAMC1-AS1 | 1.887402 | 1.34308 | 2.652327 | 0.000253 |
| AC067930.3 | 1.191502 | 0.900365 | 1.57678 | 0.220297 |
| AC110792.3 | 1.466513 | 1.036696 | 2.074533 | 0.030494 |
| AC073487.1 | 1.894629 | 1.382376 | 2.596701 | 7.09E-05 |
| LINC01023 | 0.992632 | 0.95481 | 1.031951 | 0.709055 |
| AC008608.2 | 1.002413 | 0.872822 | 1.151245 | 0.972775 |
| AP006621.2 | 1.144052 | 1.056493 | 1.238868 | 0.000924 |
| AC002467.1 | 1.413224 | 1.033493 | 1.932477 | 0.030288 |
| AC048341.1 | 1.534992 | 1.180043 | 1.996707 | 0.001404 |
| AC079921.2 | 0.69991 | 0.429872 | 1.139578 | 0.151396 |
| AC090589.3 | 1.447258 | 1.207462 | 1.734677 | 6.34E-05 |
| AC139100.2 | 1.125005 | 1.034543 | 1.223377 | 0.005888 |
| LINC00115 | 1.694696 | 1.325434 | 2.166835 | 2.59E-05 |
| AL023284.4 | 0.99368 | 0.960291 | 1.028231 | 0.716203 |
| AC069544.1 | 2.27463 | 1.434193 | 3.607563 | 0.000479 |
| AC034206.1 | 0.365003 | 0.224392 | 0.593727 | 4.90E-05 |
| AC010719.1 | 1.348962 | 1.173862 | 1.550181 | 2.45E-05 |
| AC008966.1 | 0.539407 | 0.349062 | 0.833549 | 0.005438 |
| AL118506.1 | 0.904014 | 0.675059 | 1.210623 | 0.498264 |
| APTR | 1.02901 | 0.924537 | 1.145289 | 0.600602 |
| AL157838.1 | 1.433346 | 0.991347 | 2.072415 | 0.05565 |
| AC109454.2 | 1.639878 | 0.697617 | 3.854837 | 0.256694 |
| PAXIP1-AS1 | 1.099321 | 0.990485 | 1.220115 | 0.075038 |
| AC078864.1 | 0.9906 | 0.964683 | 1.017213 | 0.485026 |
| AL080317.2 | 1.634011 | 1.176354 | 2.269719 | 0.003404 |
| AC097468.3 | 0.987442 | 0.926406 | 1.052499 | 0.697861 |
| AP001062.1 | 1.208719 | 0.857106 | 1.704575 | 0.279782 |
| AC007098.1 | 1.379012 | 0.976313 | 1.947811 | 0.068165 |
| LINC01612 | 0.808651 | 0.589013 | 1.110191 | 0.189016 |
| TRIM52-AS1 | 1.002333 | 0.917758 | 1.094701 | 0.958678 |
| AC116351.1 | 0.937698 | 0.819276 | 1.073237 | 0.350366 |
| EGOT | 0.877281 | 0.811961 | 0.947855 | 0.000911 |
| RERG-AS1 | 0.576705 | 0.384499 | 0.864993 | 0.007787 |
| PAXIP1-AS2 | 0.76739 | 0.633107 | 0.930153 | 0.006981 |
| AC024075.3 | 0.951019 | 0.733951 | 1.232286 | 0.704014 |
| AC008393.1 | 1.169331 | 0.825545 | 1.656281 | 0.378494 |
| LINC00467 | 1.153669 | 0.844455 | 1.576109 | 0.36921 |
| AL031775.2 | 1.257315 | 0.986896 | 1.601831 | 0.063852 |
| AC004585.1 | 1.245485 | 1.057526 | 1.466851 | 0.008537 |
| AC015722.2 | 1.000062 | 0.986081 | 1.014241 | 0.993145 |
| MIR600HG | 0.834485 | 0.505986 | 1.376253 | 0.478423 |
| AC012409.3 | 1.185681 | 0.972015 | 1.446316 | 0.09296 |
| AL353804.1 | 0.797996 | 0.528988 | 1.203805 | 0.282054 |
| FMR1-IT1 | 1.353715 | 1.041694 | 1.759197 | 0.02348 |
| LINC01137 | 1.003354 | 0.911675 | 1.104253 | 0.945392 |
| AL392172.1 | 1.018611 | 0.923565 | 1.123438 | 0.712152 |
| AC004832.5 | 1.244014 | 0.872098 | 1.774536 | 0.228276 |
| AC090948.2 | 0.717921 | 0.345288 | 1.492697 | 0.37489 |
| LINC01801 | 0.560185 | 0.341704 | 0.91836 | 0.021582 |
| AL157931.1 | 0.999996 | 0.987935 | 1.012203 | 0.999429 |
| AC093788.1 | 1.441099 | 1.161959 | 1.787296 | 0.00088 |
| EP300-AS1 | 0.805298 | 0.52864 | 1.226741 | 0.313289 |
| AC068473.5 | 1.109329 | 0.919139 | 1.338874 | 0.279579 |
| AC104031.1 | 0.98416 | 0.945456 | 1.024447 | 0.435373 |
| ITGB2-AS1 | 1.137948 | 1.064799 | 1.216123 | 0.000138 |
| RFX3-AS1 | 1.101823 | 0.769486 | 1.577693 | 0.596536 |
| AL031846.2 | 1.221898 | 0.817446 | 1.826464 | 0.328498 |
| AC073896.4 | 1.027083 | 1.010141 | 1.04431 | 0.001639 |
| AC020978.3 | 1.604077 | 1.252532 | 2.05429 | 0.000181 |
| AP001189.1 | 1.055987 | 0.928094 | 1.201504 | 0.408208 |
| AP001363.2 | 0.864066 | 0.592672 | 1.259735 | 0.447512 |
| LINC02275 | 0.59215 | 0.419065 | 0.836724 | 0.002973 |
| LINC01089 | 1.230163 | 1.133858 | 1.334647 | 6.35E-07 |
| LINC00641 | 1.041079 | 0.859998 | 1.260289 | 0.679658 |
| AC125494.2 | 1.194141 | 0.928557 | 1.535686 | 0.166841 |
| NALT1 | 1.632202 | 1.36656 | 1.949482 | 6.45E-08 |
| AC008554.1 | 0.759026 | 0.610121 | 0.944274 | 0.013339 |
| AC244197.2 | 1.386583 | 1.199432 | 1.602937 | 9.96E-06 |
| AC092171.5 | 0.986681 | 0.748803 | 1.300129 | 0.924109 |
| AC138207.2 | 1.171005 | 1.021855 | 1.341926 | 0.023148 |
| AC040970.1 | 1.340665 | 1.146159 | 1.568178 | 0.000247 |
| AGAP2-AS1 | 1.074057 | 1.049261 | 1.09944 | 2.03E-09 |
| AC005261.1 | 1.136259 | 1.075642 | 1.200291 | 4.95E-06 |
| AC084036.1 | 1.087573 | 1.034897 | 1.14293 | 0.000919 |
| AL080317.1 | 1.43213 | 1.103041 | 1.859402 | 0.007014 |
| AC018904.1 | 1.212395 | 1.092572 | 1.345358 | 0.000286 |
| AC108449.2 | 0.861434 | 0.761897 | 0.973974 | 0.017271 |
| AC073957.3 | 1.198275 | 0.927016 | 1.54891 | 0.1672 |
| TRHDE-AS1 | 0.680316 | 0.502158 | 0.921681 | 0.012904 |
| AC007292.1 | 1.24115 | 0.996499 | 1.545867 | 0.053774 |
| AC040169.1 | 1.095348 | 0.962312 | 1.246776 | 0.168055 |
| AP003392.1 | 1.056007 | 0.916177 | 1.217179 | 0.452078 |
| AC006042.1 | 1.171681 | 1.005617 | 1.365168 | 0.042175 |
| AC007878.1 | 0.828971 | 0.491283 | 1.39877 | 0.482238 |
| AC011468.1 | 1.211888 | 1.108324 | 1.325129 | 2.48E-05 |
| AL160006.1 | 1.128971 | 0.829348 | 1.536841 | 0.440777 |
| AL513327.1 | 1.336434 | 1.111705 | 1.606592 | 0.00202 |
| AL031714.1 | 1.712987 | 1.297943 | 2.260751 | 0.000143 |
| AC011472.1 | 1.128949 | 1.047575 | 1.216644 | 0.001485 |
| PSPC1-AS2 | 1.70315 | 1.310107 | 2.214109 | 6.96E-05 |
| LINC01503 | 1.032235 | 1.005912 | 1.059246 | 0.016074 |
| LINC02585 | 1.364061 | 0.992874 | 1.874018 | 0.055386 |
| ZNF667-AS1 | 0.884189 | 0.77857 | 1.004135 | 0.057909 |
| AC009318.3 | 1.396038 | 1.027708 | 1.896378 | 0.032774 |
| AC097359.2 | 0.943726 | 0.701224 | 1.270092 | 0.702307 |
| CA3-AS1 | 0.943409 | 0.802131 | 1.10957 | 0.481545 |
| PCED1B-AS1 | 1.127564 | 1.060909 | 1.198408 | 0.000113 |
| Z83843.1 | 1.04318 | 0.954379 | 1.140243 | 0.351708 |
| AC074124.1 | 1.143259 | 0.938204 | 1.39313 | 0.184347 |
| AC092718.4 | 1.190279 | 1.120858 | 1.263999 | 1.34E-08 |
| AC087482.1 | 0.98817 | 0.967981 | 1.00878 | 0.258487 |
| AC093297.2 | 1.008963 | 0.892636 | 1.140451 | 0.886469 |
| AL356481.3 | 1.737708 | 1.185852 | 2.54638 | 0.004592 |
| AC005288.1 | 0.943356 | 0.87822 | 1.013321 | 0.110168 |
| CDC37L1-DT | 0.928883 | 0.672009 | 1.283946 | 0.655114 |
| AC084876.1 | 1.583512 | 1.313113 | 1.909591 | 1.50E-06 |
| TMEM246-AS1 | 0.924499 | 0.79652 | 1.07304 | 0.301765 |
| AC048382.2 | 1.50277 | 1.145108 | 1.972143 | 0.003314 |
| AL121832.2 | 1.045262 | 0.926349 | 1.17944 | 0.47251 |
| DOCK9-DT | 0.405696 | 0.247365 | 0.66537 | 0.000352 |
| AL391244.2 | 1.843352 | 1.470739 | 2.310367 | 1.11E-07 |
| LINC00294 | 0.98861 | 0.906441 | 1.078228 | 0.795835 |
| AL021068.1 | 0.994186 | 0.918102 | 1.076575 | 0.88586 |
| AC079414.3 | 1.785464 | 1.316816 | 2.420902 | 0.00019 |
| TMEM44-AS1 | 1.081522 | 0.987031 | 1.185059 | 0.092936 |
| PCAT19 | 0.925196 | 0.790482 | 1.082868 | 0.332856 |
| USP46-AS1 | 0.734033 | 0.533794 | 1.009385 | 0.057108 |
| AC092542.1 | 0.93024 | 0.662331 | 1.306516 | 0.676496 |
| AC006504.7 | 1.423864 | 1.195368 | 1.696036 | 7.51E-05 |
| AL359921.2 | 1.210961 | 1.013291 | 1.447191 | 0.035276 |
| LINC01011 | 1.528704 | 1.225461 | 1.906986 | 0.000168 |
| AL606489.1 | 0.647942 | 0.473999 | 0.885717 | 0.006511 |
| AC022150.2 | 1.131165 | 0.923758 | 1.38514 | 0.233032 |
| AC008669.1 | 0.638846 | 0.477623 | 0.854492 | 0.002531 |
| AC148477.4 | 0.972485 | 0.893788 | 1.058112 | 0.516973 |
| AC105206.2 | 1.168747 | 0.762542 | 1.791338 | 0.474182 |
| AC107021.1 | 1.097312 | 0.943374 | 1.276369 | 0.228545 |
| AC007406.2 | 0.977483 | 0.943092 | 1.013127 | 0.212662 |
| LINC01508 | 0.876786 | 0.795522 | 0.966351 | 0.008056 |
| AP001318.2 | 0.965971 | 0.707222 | 1.319389 | 0.827713 |
| HOXC-AS2 | 1.189176 | 0.884153 | 1.599428 | 0.251897 |
| LINC02526 | 1.206019 | 1.086408 | 1.338799 | 0.000439 |
| AL161785.1 | 0.787631 | 0.617653 | 1.004387 | 0.054271 |
| AL049552.1 | 1.551612 | 1.110969 | 2.167027 | 0.009955 |
| MHENCR | 1.086646 | 1.050482 | 1.124054 | 1.49E-06 |
| DHRS4-AS1 | 0.864835 | 0.790461 | 0.946206 | 0.00155 |
| AC232271.1 | 1.884594 | 1.449914 | 2.449591 | 2.17E-06 |
| AC024075.2 | 1.170081 | 1.057685 | 1.294421 | 0.002301 |
| AC009060.1 | 0.878704 | 0.715627 | 1.078943 | 0.217002 |
| AC009120.3 | 1.161583 | 0.993085 | 1.35867 | 0.061043 |
| AC098483.1 | 0.703988 | 0.510632 | 0.970561 | 0.032165 |
| AC013403.2 | 1.578287 | 0.88734 | 2.807253 | 0.120386 |
| NKILA | 1.193132 | 1.010104 | 1.409325 | 0.037683 |
| LINC01507 | 0.712526 | 0.619908 | 0.818981 | 1.83E-06 |
| AL662844.4 | 1.004425 | 0.794363 | 1.270036 | 0.970578 |
| COLCA1 | 0.691245 | 0.502412 | 0.95105 | 0.023314 |
| SCGB1B2P | 1.212769 | 1.103098 | 1.333344 | 6.64E-05 |
| AC004825.2 | 1.292183 | 0.937548 | 1.780961 | 0.11735 |
| AC012615.1 | 1.468462 | 1.223694 | 1.76219 | 3.63E-05 |
| ALDH1L1-AS2 | 0.569804 | 0.38996 | 0.83259 | 0.003651 |
| AC009053.2 | 1.22372 | 0.78723 | 1.902227 | 0.369703 |
| AC008760.1 | 1.451172 | 1.163632 | 1.809764 | 0.00095 |
| LINC01144 | 1.187371 | 0.769194 | 1.832891 | 0.438151 |
| AC080162.1 | 0.991155 | 0.584439 | 1.68091 | 0.973702 |
| AC005261.3 | 1.225956 | 1.146074 | 1.311407 | 3.10E-09 |
| LINC01786 | 1.332583 | 1.105218 | 1.606722 | 0.002629 |
| AC008759.3 | 0.957563 | 0.753803 | 1.216401 | 0.722421 |
| AC129492.1 | 0.754397 | 0.490144 | 1.161116 | 0.200195 |
| AL353622.1 | 1.204529 | 1.08433 | 1.338052 | 0.000522 |
| AC055822.1 | 1.146592 | 1.017292 | 1.292325 | 0.025039 |
| SMIM2-AS1 | 0.954084 | 0.912925 | 0.997097 | 0.036693 |
| AC092894.1 | 0.536517 | 0.376115 | 0.765326 | 0.000591 |
| AL591895.1 | 1.01898 | 0.997541 | 1.04088 | 0.083093 |
| AC136475.2 | 1.077211 | 1.000309 | 1.160025 | 0.049052 |
| AC103563.7 | 0.889398 | 0.70816 | 1.117019 | 0.313388 |
| AC103702.2 | 1.07621 | 1.034611 | 1.119481 | 0.000261 |
| AP003717.1 | 1.209637 | 1.02901 | 1.42197 | 0.02108 |
| SDCBP2-AS1 | 1.055727 | 0.72329 | 1.540958 | 0.778666 |
| AC090425.2 | 1.034021 | 0.933024 | 1.145952 | 0.52349 |
| AC025265.1 | 1.127649 | 1.052268 | 1.20843 | 0.000666 |
| TEX41 | 0.966765 | 0.767965 | 1.217026 | 0.773524 |
| AC004923.4 | 1.905925 | 1.360046 | 2.670903 | 0.00018 |
| ADORA2A-AS1 | 0.853194 | 0.750388 | 0.970084 | 0.015367 |
| AC145423.3 | 1.719505 | 1.290741 | 2.290697 | 0.000212 |
| U62317.2 | 1.080301 | 1.048558 | 1.113006 | 3.86E-07 |
| AL512598.1 | 1.384265 | 1.047832 | 1.82872 | 0.022088 |
| AC073115.2 | 0.981506 | 0.93072 | 1.035062 | 0.491042 |
| AC005674.1 | 0.394415 | 0.184133 | 0.844841 | 0.016675 |
| AC120053.1 | 1.334485 | 1.193468 | 1.492164 | 4.11E-07 |
| USP30-AS1 | 1.121473 | 1.014889 | 1.23925 | 0.024448 |
| LINC02446 | 1.109085 | 1.046359 | 1.175571 | 0.000491 |
| AL162274.2 | 1.238287 | 1.018133 | 1.506046 | 0.032364 |
| AP001462.1 | 1.147866 | 0.803646 | 1.639524 | 0.448349 |
| AC002091.2 | 0.969248 | 0.616984 | 1.522635 | 0.892187 |
| AC129507.4 | 1.02817 | 0.98377 | 1.074575 | 0.217407 |
| AC131009.3 | 1.167409 | 1.032771 | 1.319599 | 0.013297 |
| AC010969.2 | 1.203826 | 0.939659 | 1.542258 | 0.142219 |
| PCBP1-AS1 | 1.104363 | 0.746063 | 1.634738 | 0.619849 |
| LINP1 | 0.976968 | 0.878428 | 1.086563 | 0.667524 |
| AL513477.2 | 1.660778 | 1.287326 | 2.14257 | 9.49E-05 |
| AC087741.2 | 0.890961 | 0.761477 | 1.042463 | 0.149599 |
| MIR210HG | 1.015941 | 0.987956 | 1.044719 | 0.267126 |
| AC244035.1 | 1.080429 | 0.802556 | 1.45451 | 0.610074 |
| HID1-AS1 | 0.809691 | 0.553996 | 1.183403 | 0.275595 |
| ASMTL-AS1 | 1.056142 | 1.029934 | 1.083018 | 2.04E-05 |
| AC004687.1 | 1.236574 | 1.066168 | 1.434218 | 0.005002 |
| AP000695.2 | 1.325187 | 1.147695 | 1.530129 | 0.000124 |
| KLF3-AS1 | 0.857138 | 0.562843 | 1.30531 | 0.472533 |
| AC017048.3 | 1.084543 | 0.970733 | 1.211697 | 0.151337 |
| AC116366.2 | 1.280485 | 1.010772 | 1.622167 | 0.040487 |
| AC009065.3 | 1.141046 | 1.041269 | 1.250384 | 0.004711 |
| LINC01857 | 1.137358 | 1.009452 | 1.28147 | 0.034471 |
| STARD4-AS1 | 1.008273 | 0.839004 | 1.21169 | 0.929986 |
| AC005332.4 | 1.215081 | 0.991883 | 1.488506 | 0.059937 |
| SOS1-IT1 | 0.980201 | 0.69037 | 1.391707 | 0.910968 |
| PPP1R26-AS1 | 1.048739 | 0.854244 | 1.287518 | 0.649321 |
| AC083799.1 | 1.109701 | 1.03575 | 1.188931 | 0.003094 |
| LINC01480 | 1.216221 | 1.044463 | 1.416224 | 0.011735 |
| DGUOK-AS1 | 2.500613 | 1.721537 | 3.632257 | 1.49E-06 |
| AL136295.7 | 1.271199 | 1.090588 | 1.48172 | 0.002147 |
| AL731533.2 | 0.985196 | 0.910358 | 1.066185 | 0.711361 |
| AL136084.3 | 0.813684 | 0.64275 | 1.030077 | 0.08659 |
| AL021707.7 | 1.334877 | 0.93204 | 1.911824 | 0.115035 |
| AL358472.3 | 2.228815 | 1.708775 | 2.907123 | 3.37E-09 |
| PTOV1-AS2 | 1.149659 | 1.069049 | 1.236347 | 0.00017 |
| AC025165.5 | 1.575254 | 1.112973 | 2.229547 | 0.010351 |
| MAGI2-AS3 | 0.982815 | 0.80101 | 1.205886 | 0.868084 |
| AC005519.1 | 1.398876 | 1.087132 | 1.800015 | 0.00907 |
| AP002807.1 | 1.48127 | 1.275523 | 1.720204 | 2.61E-07 |
| AC022784.1 | 1.201476 | 0.953204 | 1.514414 | 0.120145 |
| AC156455.1 | 1.258951 | 1.161195 | 1.364936 | 2.35E-08 |
| AC068580.3 | 1.128898 | 0.993975 | 1.282137 | 0.061915 |
| AL132989.1 | 1.130016 | 0.996917 | 1.280885 | 0.055918 |
| AC022144.1 | 1.021565 | 0.984987 | 1.059502 | 0.251443 |
| LINC01094 | 1.143499 | 0.970176 | 1.347786 | 0.109837 |
| AC007743.1 | 0.634107 | 0.448996 | 0.895535 | 0.009698 |
| AC006547.1 | 1.137519 | 0.971194 | 1.332329 | 0.11014 |
| AP000692.1 | 1.587166 | 1.17661 | 2.140978 | 0.002487 |
| AC011450.1 | 1.0964 | 0.878443 | 1.368437 | 0.415726 |
| ATP6V0E2-AS1 | 1.136156 | 0.856567 | 1.507005 | 0.37577 |
| A2M-AS1 | 1.220703 | 0.825748 | 1.804566 | 0.317339 |
| AL031716.1 | 1.116635 | 0.775728 | 1.607359 | 0.552797 |
| AC004908.1 | 1.232207 | 1.150372 | 1.319864 | 2.60E-09 |
| AC118754.1 | 0.899018 | 0.639789 | 1.263281 | 0.53964 |
| AL049555.1 | 0.910699 | 0.709323 | 1.169246 | 0.463163 |
| ZNF503-AS2 | 0.859218 | 0.568898 | 1.297693 | 0.470749 |
| AC145207.5 | 1.826357 | 1.133969 | 2.941511 | 0.01325 |
| LINC02035 | 0.805289 | 0.579278 | 1.119481 | 0.197591 |
| LINC00899 | 1.118529 | 0.90224 | 1.386668 | 0.30694 |
| Z68871.1 | 0.584146 | 0.299186 | 1.140517 | 0.1153 |
| AL049780.1 | 1.491416 | 1.177813 | 1.888518 | 0.000904 |
| DNM3OS | 1.250123 | 0.857461 | 1.822598 | 0.245831 |
| AC066613.1 | 1.919448 | 1.280948 | 2.876215 | 0.001578 |
| AC037198.1 | 1.205739 | 1.087812 | 1.33645 | 0.000367 |
| NFYC-AS1 | 1.31986 | 1.012139 | 1.721138 | 0.040458 |
| LINC00662 | 1.531604 | 0.699476 | 3.353669 | 0.286368 |
| MIR3936HG | 1.014689 | 0.943557 | 1.091182 | 0.694151 |
| AL035071.1 | 1.176163 | 1.095298 | 1.262998 | 8.02E-06 |
| BHLHE40-AS1 | 1.117504 | 0.867138 | 1.440158 | 0.39065 |
| ASH1L-AS1 | 1.554217 | 1.098099 | 2.199794 | 0.012849 |
| AC107952.2 | 0.980135 | 0.699584 | 1.373195 | 0.907159 |
| TRAM2-AS1 | 0.740927 | 0.624965 | 0.878405 | 0.000555 |
| LINC00997 | 1.191372 | 1.077351 | 1.31746 | 0.000646 |
| MELTF-AS1 | 1.156465 | 1.106716 | 1.208451 | 9.19E-11 |
| AL359076.1 | 1.001893 | 0.961403 | 1.044087 | 0.928421 |
| ZNF32-AS1 | 2.111151 | 1.478687 | 3.014133 | 3.91E-05 |
| AC008764.2 | 0.985833 | 0.867568 | 1.120221 | 0.826785 |
| AC002070.1 | 0.648589 | 0.511501 | 0.822419 | 0.000352 |
| LINC01550 | 0.384511 | 0.260859 | 0.566776 | 1.38E-06 |
| AC009779.2 | 1.198 | 0.972611 | 1.47562 | 0.089355 |
| AL365181.2 | 1.276366 | 1.08853 | 1.496614 | 0.002661 |
| AC135050.5 | 1.06832 | 0.866288 | 1.317469 | 0.536638 |
| MIR100HG | 1.095302 | 1.00011 | 1.199555 | 0.049723 |
| AC021078.1 | 1.145035 | 1.042615 | 1.257516 | 0.004614 |
| AL109614.1 | 0.82603 | 0.494903 | 1.378706 | 0.464627 |
| AL121899.1 | 0.945206 | 0.763204 | 1.17061 | 0.605566 |
| AC024361.1 | 1.215422 | 0.934554 | 1.5807 | 0.145636 |
| AL035661.1 | 0.982203 | 0.947261 | 1.018434 | 0.331243 |
| LUCAT1 | 1.075963 | 1.028073 | 1.126084 | 0.001623 |
| DBH-AS1 | 1.167712 | 1.101838 | 1.237524 | 1.66E-07 |
| AC073896.2 | 1.285933 | 1.002917 | 1.648814 | 0.047375 |
| AC002401.2 | 0.759542 | 0.5401 | 1.068143 | 0.113873 |
| AL136295.2 | 1.546899 | 1.152513 | 2.076244 | 0.00367 |
| AC018755.4 | 1.124045 | 0.987388 | 1.279616 | 0.077051 |
| LINC00240 | 1.522126 | 1.042495 | 2.222427 | 0.029595 |
| SOCS2-AS1 | 1.040489 | 0.722987 | 1.497421 | 0.830795 |
| AC060766.7 | 1.122197 | 0.844094 | 1.491926 | 0.427511 |
| AC104825.1 | 0.982684 | 0.722313 | 1.336911 | 0.911446 |
| AL035446.1 | 1.162234 | 1.010032 | 1.337372 | 0.035786 |
| AC005837.3 | 1.134643 | 1.046238 | 1.230518 | 0.002272 |
| AC048344.4 | 1.392475 | 1.140872 | 1.699566 | 0.001129 |
| NPTN-IT1 | 0.987512 | 0.684225 | 1.425232 | 0.946479 |
| ASB16-AS1 | 1.495192 | 1.268291 | 1.762687 | 1.67E-06 |
| LINC00853 | 0.96417 | 0.807218 | 1.151638 | 0.687308 |
| AC107021.2 | 1.085846 | 1.038325 | 1.135541 | 0.00031 |
| AF064858.2 | 0.968778 | 0.933438 | 1.005457 | 0.094338 |
| AC008115.3 | 1.139441 | 0.920568 | 1.410354 | 0.230347 |
| TPRG1-AS1 | 0.700241 | 0.410882 | 1.193376 | 0.190188 |
| MIR155HG | 1.070786 | 1.032232 | 1.110779 | 0.000257 |
| AC005332.6 | 0.907286 | 0.846609 | 0.972313 | 0.005869 |
| CKMT2-AS1 | 0.957203 | 0.654215 | 1.400515 | 0.82178 |
| AC027020.2 | 1.24393 | 1.01787 | 1.520195 | 0.03292 |
| AC005840.4 | 1.228153 | 1.037914 | 1.453261 | 0.016696 |
| LINC02048 | 1.086956 | 0.975996 | 1.21053 | 0.129087 |
| AC004921.1 | 1.372185 | 0.926648 | 2.031938 | 0.114191 |
| AC007996.1 | 0.899185 | 0.684371 | 1.181425 | 0.445487 |
| ZNF529-AS1 | 1.689581 | 1.373224 | 2.078819 | 7.11E-07 |
| LINC01278 | 1.075407 | 0.937137 | 1.234076 | 0.300513 |
| AC093752.3 | 1.395399 | 1.030465 | 1.889572 | 0.031242 |
| AL137186.2 | 1.796122 | 1.385915 | 2.327743 | 9.55E-06 |
| RASSF8-AS1 | 1.235645 | 1.019743 | 1.497259 | 0.030812 |
| ZSCAN16-AS1 | 0.975465 | 0.900097 | 1.057144 | 0.544863 |
| AC105020.1 | 1.217784 | 1.061981 | 1.396445 | 0.004788 |
| AC120498.10 | 1.092291 | 1.025664 | 1.163246 | 0.005976 |
| CFAP58-DT | 0.992756 | 0.727214 | 1.355259 | 0.963484 |
| LINC01871 | 1.205608 | 1.114022 | 1.304725 | 3.51E-06 |
| AL596442.2 | 1.166073 | 1.031228 | 1.318549 | 0.01427 |
| LINC-PINT | 1.030896 | 0.987088 | 1.076648 | 0.16963 |
| AC079015.1 | 1.212675 | 0.87386 | 1.682857 | 0.248734 |
| UBR5-AS1 | 1.376775 | 1.123898 | 1.686549 | 0.002015 |
| AL122035.1 | 0.702791 | 0.546961 | 0.903016 | 0.005823 |
| AL031600.1 | 1.357473 | 1.128787 | 1.632491 | 0.001166 |
| AC074117.1 | 1.736566 | 1.406355 | 2.14431 | 2.91E-07 |
| CD27-AS1 | 1.14066 | 1.092958 | 1.190445 | 1.56E-09 |
| AL049840.2 | 1.050958 | 0.930627 | 1.186849 | 0.423067 |
| AC010186.3 | 1.214983 | 0.974579 | 1.51469 | 0.083441 |
| AC000120.1 | 1.109063 | 0.981315 | 1.25344 | 0.097341 |
| AC007406.5 | 0.891258 | 0.712791 | 1.114409 | 0.312595 |
| PANTR1 | 0.985291 | 0.9703 | 1.000514 | 0.058182 |
| AC138207.5 | 1.105483 | 1.03141 | 1.184875 | 0.004598 |
| AC087500.1 | 1.514036 | 1.014149 | 2.260325 | 0.042491 |
| MIR3142HG | 1.361543 | 0.970159 | 1.910822 | 0.0743 |
| AC020594.1 | 1.744408 | 1.435394 | 2.119946 | 2.23E-08 |
| AC022306.3 | 1.473057 | 0.957806 | 2.265487 | 0.077787 |
| AL117379.1 | 1.273764 | 1.149362 | 1.411631 | 3.93E-06 |
| AP006621.4 | 1.159847 | 0.989217 | 1.359908 | 0.067785 |
| EPB41L4A-AS1 | 0.976748 | 0.944679 | 1.009905 | 0.167194 |
| PRKAG2-AS1 | 1.185687 | 1.075548 | 1.307104 | 0.000617 |
| AC096586.2 | 1.131155 | 0.801406 | 1.596583 | 0.483373 |
| AC005899.7 | 1.3856 | 1.170446 | 1.640305 | 0.000152 |
| AC025580.3 | 0.694643 | 0.460026 | 1.048915 | 0.083124 |
| AL355001.2 | 0.987405 | 0.780122 | 1.249763 | 0.916033 |
| AC100810.1 | 0.983255 | 0.929984 | 1.039579 | 0.552395 |
| TOLLIP-AS1 | 0.989739 | 0.679976 | 1.440613 | 0.957052 |
| AL691432.2 | 0.872809 | 0.720459 | 1.057377 | 0.164549 |
| AL359513.1 | 1.439771 | 1.134049 | 1.827911 | 0.002763 |
| SPART-AS1 | 1.991296 | 1.499784 | 2.643888 | 1.91E-06 |
| AC007114.1 | 0.904366 | 0.706021 | 1.158433 | 0.42618 |
| AC015911.3 | 1.338477 | 1.030297 | 1.73884 | 0.028998 |
| AC002401.1 | 0.792818 | 0.624427 | 1.006618 | 0.056674 |
| HCG11 | 0.935643 | 0.844748 | 1.03632 | 0.20204 |
| AL391834.1 | 0.975066 | 0.765348 | 1.242252 | 0.83808 |
| AC022034.1 | 1.017214 | 0.604481 | 1.711756 | 0.948751 |
| AL021707.6 | 1.096603 | 1.025931 | 1.172144 | 0.006664 |
| MKLN1-AS | 0.795793 | 0.505446 | 1.252924 | 0.323975 |
| SNHG22 | 1.21384 | 0.87954 | 1.675202 | 0.238385 |
| AC005253.1 | 1.538815 | 1.152378 | 2.054838 | 0.003487 |
| LINC01679 | 1.061431 | 0.966802 | 1.165321 | 0.210812 |
| AC011481.1 | 1.518207 | 1.264008 | 1.823528 | 7.97E-06 |
| AL049840.5 | 1.116172 | 0.889314 | 1.4009 | 0.343095 |
| AP001107.4 | 1.208567 | 0.979534 | 1.491152 | 0.077216 |
| AC005034.4 | 1.073977 | 0.903686 | 1.276357 | 0.417808 |
| LINC00685 | 1.214679 | 1.099446 | 1.34199 | 0.000131 |
| AC139887.1 | 1.085529 | 0.798666 | 1.475428 | 0.600178 |
| AC010168.2 | 1.450854 | 1.141021 | 1.844819 | 0.002395 |
| SENCR | 1.446943 | 1.094387 | 1.913075 | 0.009515 |
| AC027682.6 | 0.932281 | 0.800303 | 1.086024 | 0.367931 |
| MIR497HG | 1.3314 | 1.029569 | 1.721716 | 0.029101 |
| AC018695.6 | 1.271359 | 1.097851 | 1.472289 | 0.001342 |
| AD001527.1 | 1.856937 | 1.397092 | 2.468138 | 2.01E-05 |
| LINC00987 | 0.88177 | 0.677511 | 1.14761 | 0.349334 |
| LINC01738 | 0.849614 | 0.716383 | 1.007623 | 0.061109 |
| AL035413.1 | 0.778861 | 0.677737 | 0.895074 | 0.000428 |
| EBLN3P | 0.940979 | 0.890721 | 0.994073 | 0.029839 |
| AL357079.1 | 1.015013 | 0.832079 | 1.238165 | 0.88316 |
| AC026356.2 | 1.427474 | 1.225044 | 1.663353 | 5.08E-06 |
| AL450384.2 | 1.546908 | 1.253638 | 1.908784 | 4.75E-05 |
| AC087500.2 | 1.728322 | 1.223687 | 2.441065 | 0.001897 |
| SPINT1-AS1 | 0.875969 | 0.791555 | 0.969385 | 0.010426 |
| AC093227.1 | 1.23175 | 0.995169 | 1.524572 | 0.055434 |
| AC132872.3 | 1.04194 | 1.012184 | 1.072571 | 0.00545 |
| AL390728.6 | 1.073182 | 1.037631 | 1.109951 | 3.97E-05 |
| AL353801.1 | 0.80919 | 0.667614 | 0.980789 | 0.030955 |
| AC068888.1 | 1.105307 | 0.907576 | 1.346117 | 0.319437 |
| AC127024.5 | 1.392053 | 1.171212 | 1.654536 | 0.000175 |
| AL078581.1 | 0.860625 | 0.707076 | 1.047518 | 0.134402 |
| GAS5-AS1 | 0.710851 | 0.473365 | 1.067483 | 0.099934 |
| PAX8-AS1 | 1.197929 | 1.054394 | 1.361003 | 0.005548 |
| AF131215.6 | 1.379564 | 1.063735 | 1.789165 | 0.015276 |
| AC092119.2 | 1.9925 | 1.554017 | 2.554707 | 5.44E-08 |
| FAM160A1-DT | 0.444374 | 0.235881 | 0.837153 | 0.012072 |
| PSMA3-AS1 | 1.085583 | 1.013027 | 1.163336 | 0.019981 |
| AL136304.1 | 1.560774 | 1.227697 | 1.984215 | 0.000278 |
| AC019257.1 | 1.078676 | 1.043221 | 1.115336 | 8.94E-06 |
| AP000757.1 | 0.977333 | 0.907682 | 1.052328 | 0.543303 |
| AL162724.2 | 1.073157 | 0.937079 | 1.228997 | 0.307455 |
| AC108463.2 | 0.98591 | 0.742145 | 1.309743 | 0.921995 |
| AL590822.1 | 1.065211 | 0.917058 | 1.237299 | 0.408362 |
| LINC01187 | 0.996988 | 0.989292 | 1.004743 | 0.445411 |
| AC004816.1 | 1.157523 | 0.841489 | 1.592248 | 0.36857 |
| DUBR | 0.429612 | 0.256164 | 0.720502 | 0.001362 |
| AC009779.4 | 1.283279 | 1.108564 | 1.485529 | 0.000837 |
| AP001160.3 | 0.552168 | 0.397173 | 0.767649 | 0.000411 |
| AC095057.3 | 2.146311 | 1.568994 | 2.936054 | 1.77E-06 |
| AL355803.1 | 0.897577 | 0.787805 | 1.022645 | 0.104477 |
| AC090559.1 | 0.958487 | 0.847048 | 1.084587 | 0.501364 |
| AC023043.4 | 1.43761 | 1.273932 | 1.622318 | 3.96E-09 |
| AC009084.1 | 0.929013 | 0.834058 | 1.034779 | 0.180734 |
| AC008906.1 | 1.283607 | 0.993299 | 1.658762 | 0.056318 |
| AC092279.1 | 0.867231 | 0.579797 | 1.297159 | 0.488033 |
| CTBP1-DT | 0.725246 | 0.532126 | 0.988453 | 0.042004 |
| TAPT1-AS1 | 0.995811 | 0.534986 | 1.85358 | 0.989435 |
| AL139089.1 | 1.867455 | 1.428827 | 2.440735 | 4.82E-06 |
| AC068790.5 | 1.527796 | 1.182934 | 1.973196 | 0.001166 |
| AC023794.5 | 1.113578 | 0.838544 | 1.478821 | 0.457301 |
| AC243960.1 | 1.28914 | 1.138688 | 1.459471 | 6.04E-05 |
| AF064858.1 | 1.022694 | 0.934231 | 1.119533 | 0.626865 |
| AC020915.3 | 1.097776 | 0.820468 | 1.46881 | 0.530036 |
| AC006001.2 | 1.328273 | 0.99278 | 1.77714 | 0.055982 |
| AP001528.1 | 0.751711 | 0.640164 | 0.882695 | 0.000497 |
| AL022328.1 | 1.087091 | 0.89176 | 1.325208 | 0.408612 |
| LNCSRLR | 1.090089 | 0.967748 | 1.227895 | 0.155548 |
| AC026979.2 | 1.037403 | 0.972141 | 1.107047 | 0.268003 |
| AC011476.3 | 0.921222 | 0.781833 | 1.085461 | 0.326947 |
| AC083967.1 | 1.14337 | 1.031072 | 1.267899 | 0.011083 |
| AC025580.1 | 0.989755 | 0.894918 | 1.094642 | 0.841185 |
| AC110995.1 | 0.901771 | 0.573266 | 1.418523 | 0.65463 |
| TPT1-AS1 | 1.672256 | 1.228835 | 2.275685 | 0.001072 |
| AL022328.2 | 1.139375 | 1.017547 | 1.275789 | 0.023732 |
| TRG-AS1 | 1.393991 | 0.989438 | 1.963953 | 0.057531 |
| AP001505.1 | 1.027945 | 0.99396 | 1.063092 | 0.108108 |
| AL133367.1 | 1.141398 | 0.858083 | 1.518255 | 0.363596 |
| AC020916.1 | 1.006957 | 0.998497 | 1.015489 | 0.107269 |
| AC027031.2 | 1.057234 | 1.026256 | 1.089146 | 0.000244 |
| ZNF582-AS1 | 0.804967 | 0.531247 | 1.219717 | 0.306204 |
| AC018647.2 | 0.681703 | 0.535792 | 0.867349 | 0.00182 |
| HPN-AS1 | 1.113932 | 0.925936 | 1.340097 | 0.252604 |
| SLC16A1-AS1 | 3.324979 | 2.17004 | 5.0946 | 3.42E-08 |
| LINC00857 | 1.273721 | 0.96387 | 1.683179 | 0.088903 |
| TRAPPC12-AS1 | 1.587776 | 1.206875 | 2.088893 | 0.000955 |
| AL117209.1 | 1.539289 | 1.127415 | 2.101632 | 0.006631 |
| AC136475.1 | 1.115199 | 0.739884 | 1.680896 | 0.602474 |
| LINC02256 | 0.895724 | 0.546719 | 1.467521 | 0.661978 |
| AL355075.4 | 0.963863 | 0.805268 | 1.153694 | 0.688221 |
| AC087623.1 | 1.281916 | 1.094687 | 1.501167 | 0.002049 |
| AC135178.4 | 1.357463 | 0.687364 | 2.680833 | 0.378739 |
| ACTA2-AS1 | 1.224956 | 1.043836 | 1.437503 | 0.012937 |
| AL356356.1 | 1.320098 | 1.148856 | 1.516864 | 8.95E-05 |
| AC090617.5 | 0.961911 | 0.800888 | 1.155309 | 0.677811 |
| ZNF528-AS1 | 1.234908 | 1.09175 | 1.396838 | 0.00079 |
| AC008537.2 | 0.792365 | 0.503753 | 1.246329 | 0.313889 |
| UBA6-AS1 | 0.879426 | 0.60427 | 1.279876 | 0.502161 |
| MIR29B2CHG | 1.080071 | 0.842838 | 1.384077 | 0.542703 |
