# Supplementary material for: Pyroptosis-Related lncRNA Prognostic Model for Renal Cancer Contributes to Immunodiagnosis and Immunotherapy
Source: Front Oncol. 2022 Jul 4;12:837155. doi: 10.3389/fonc.2022.837155 (PMC9291251; doi:10.3389/fonc.2022.837155)
Supplement: Supplementary file 5 [file Table_2.docx]

**Supplementary Table S2 multivariate cox regression analysis of training set**

| **id** | **coef** | **HR** | **HR.95L** | **HR.95H** | **pvalue** |
| --- | --- | --- | --- | --- | --- |
| U62317.1 | 0.150748966 | 1.162705 | 1.012925 | 1.334632 | 0.032155 |
| MIR193BHG | 0.166669613 | 1.181364 | 0.993419 | 1.404866 | 0.059392 |
| LINC02027 | -0.272022183 | 0.761837 | 0.607572 | 0.955272 | 0.018456 |
| AC121338.2 | -0.949481971 | 0.386941 | 0.214069 | 0.699419 | 0.001669 |
| AC005785.1 | 0.375208928 | 1.455295 | 0.997734 | 2.122694 | 0.051393 |
| AC156455.1 | 0.100810118 | 1.106067 | 1.001405 | 1.221667 | 0.046851 |
