# Supplementary material for: Pyroptosis-Related lncRNA Prognostic Model for Renal Cancer Contributes to Immunodiagnosis and Immunotherapy
Source: Front Oncol. 2022 Jul 4;12:837155. doi: 10.3389/fonc.2022.837155 (PMC9291251; doi:10.3389/fonc.2022.837155)
Supplement: Supplementary file 6 [file Table_3.docx]

**Supplementary Table S3** Oligonucleotide sequences used in this study

| Primes and probes |  | |  | | Sequences |
| --- | --- | --- | --- | --- | --- |
| U62317.1 | | Forward | | 5′-CCTCTGTGATCCAGCAGGTG-3′ | |
|  |  | Reverse | | 5′-GACAGGAGTGACAGGTGTGG-3′ | |
| MIR193BHG | | Forward | | 5′- TTAAGGCTGGGCTCCAATCG -3′ | |
|  |  | Reverse | | 5′- TTCAATGGCAGCAGGAGGTT -3′ | |
| LINC02027 | | Forward | | 5′- GGAACAAAGCACAGTTGGGG -3′ | |
|  |  | Reverse | | 5′- GGCCCCTCCTCTTAGAATGG -3′ | |
| AC121338.2 | | Forward | | 5′-CATCGACCGTCGTCCATAGG-3′ | |
|  | | Reverse | | 5′-GTGAGTTTTCCGCAGCCATC-3′ | |
| AC005785.1 | | Forward | | 5′- TTTGTGGCGTCTTCGGAAGA -3′ | |
| AC156455.1 | | Reverse | | 5′- TCCCACCAGCAGAGAAGACT -3′ | |
|  |  | Reverse | | 5′- AATCGGGATCCTGTTTGGT -3′ | |
| β-actin | | Forward | | 5′- TCTCTTCCTGAAATCTCTGGG -3′ | |
|  |  | Reverse | | 5′- CTGAACCCCAAGGCCAACAG -3′ | |
|  | | Forward | | 5′- CCAGAGAAGAGGAGGATGCG-3′ | |
|  |  |  | |  | |
