# Supplementary material for: Pyroptosis-Related lncRNA Prognostic Model for Renal Cancer Contributes to Immunodiagnosis and Immunotherapy
Source: Front Oncol. 2022 Jul 4;12:837155. doi: 10.3389/fonc.2022.837155 (PMC9291251; doi:10.3389/fonc.2022.837155)
Supplement: Supplementary file 7 [file Table_4.docx]

**Supplementary Table S4** The TMB Scores of KIRC

| TCGA-B0-5108-01A | 1.394737 | KIRC |
| --- | --- | --- |
| TCGA-B0-5691-01A | 1.5 | KIRC |
| TCGA-A3-3331-01A | 2 | KIRC |
| TCGA-T7-A92I-01A | 1 | KIRC |
| TCGA-CJ-4901-01A | 1.342105 | KIRC |
| TCGA-B0-5109-01A | 1.394737 | KIRC |
| TCGA-B8-5552-01B | 0.710526 | KIRC |
| TCGA-CZ-5986-01A | 1.815789 | KIRC |
| TCGA-B0-5701-01A | 3.052632 | KIRC |
| TCGA-A3-3385-01A | 1.894737 | KIRC |
| TCGA-CZ-5988-01A | 1.342105 | KIRC |
| TCGA-BP-4963-01A | 2.657895 | KIRC |
| TCGA-CZ-5985-01A | 1.631579 | KIRC |
| TCGA-B0-5110-01A | 1.894737 | KIRC |
| TCGA-CZ-5989-01A | 1.105263 | KIRC |
| TCGA-BP-5200-01A | 1.289474 | KIRC |
| TCGA-B0-5106-01A | 2.526316 | KIRC |
| TCGA-A3-3374-01A | 0.447368 | KIRC |
| TCGA-BP-4981-01A | 1.473684 | KIRC |
| TCGA-B0-4842-01A | 1.552632 | KIRC |
| TCGA-B0-5706-01A | 1.736842 | KIRC |
| TCGA-BP-5176-01A | 3.552632 | KIRC |
| TCGA-BP-4989-01A | 2 | KIRC |
| TCGA-EU-5906-01A | 1.736842 | KIRC |
| TCGA-CJ-4882-01A | 1.736842 | KIRC |
| TCGA-B0-5710-01A | 1.157895 | KIRC |
| TCGA-BP-5184-01A | 0.815789 | KIRC |
| TCGA-BP-4988-01A | 0.815789 | KIRC |
| TCGA-BP-4985-01A | 2.236842 | KIRC |
| TCGA-B0-5696-01A | 1.421053 | KIRC |
| TCGA-BP-4986-01A | 1.131579 | KIRC |
| TCGA-B0-5709-01A | 2.052632 | KIRC |
| TCGA-B2-4102-01A | 2.236842 | KIRC |
| TCGA-BP-4962-01A | 0.921053 | KIRC |
| TCGA-B0-5698-01A | 2.157895 | KIRC |
| TCGA-B0-5695-01A | 1.763158 | KIRC |
| TCGA-A3-3363-01A | 1.368421 | KIRC |
| TCGA-BP-5175-01A | 1.157895 | KIRC |
| TCGA-BP-5178-01A | 1.789474 | KIRC |
| TCGA-MM-A84U-01A | 2.736842 | KIRC |
| TCGA-BP-4974-01A | 0.894737 | KIRC |
| TCGA-B0-5705-01A | 2.394737 | KIRC |
| TCGA-B0-5699-01A | 1.473684 | KIRC |
| TCGA-EU-5905-01A | 1.710526 | KIRC |
| TCGA-BP-5000-01A | 1.315789 | KIRC |
| TCGA-CJ-4869-01A | 2.105263 | KIRC |
| TCGA-CJ-4905-01A | 1.736842 | KIRC |
| TCGA-CJ-4908-01A | 0.868421 | KIRC |
| TCGA-GK-A6C7-01A | 3.315789 | KIRC |
| TCGA-CJ-5676-01A | 1.736842 | KIRC |
| TCGA-BP-5187-01A | 1.552632 | KIRC |
| TCGA-AK-3455-01A | 1.736842 | KIRC |
| TCGA-B8-5545-01A | 0.631579 | KIRC |
| TCGA-A3-3311-01A | 1.684211 | KIRC |
| TCGA-CJ-4912-01A | 2.447368 | KIRC |
| TCGA-CJ-5684-01A | 1.421053 | KIRC |
| TCGA-B4-5836-01A | 2.131579 | KIRC |
| TCGA-B0-5712-01A | 2.973684 | KIRC |
| TCGA-B8-5549-01A | 1.789474 | KIRC |
| TCGA-BP-4977-01A | 1.368421 | KIRC |
| TCGA-B4-5838-01A | 1.815789 | KIRC |
| TCGA-CJ-5679-01A | 2.736842 | KIRC |
| TCGA-B4-5835-01A | 1.868421 | KIRC |
| TCGA-BP-4960-01A | 1.789474 | KIRC |
| TCGA-B0-5113-01A | 1.131579 | KIRC |
| TCGA-DV-5574-01A | 0.315789 | KIRC |
| TCGA-BP-4795-01A | 0.657895 | KIRC |
| TCGA-BP-4177-01A | 0.421053 | KIRC |
| TCGA-A3-A6NN-01A | 2.947368 | KIRC |
| TCGA-CJ-5675-01A | 1.789474 | KIRC |
| TCGA-AK-3427-01A | 0.447368 | KIRC |
| TCGA-CJ-5678-01A | 1.5 | KIRC |
| TCGA-B4-5844-01A | 1.736842 | KIRC |
| TCGA-BP-4991-01A | 1.394737 | KIRC |
| TCGA-B8-5546-01A | 0.368421 | KIRC |
| TCGA-DV-A4W0-01A | 1.947368 | KIRC |
| TCGA-B0-5812-01A | 1.342105 | KIRC |
| TCGA-BP-4760-01A | 0.315789 | KIRC |
| TCGA-BP-4999-01A | 1.368421 | KIRC |
| TCGA-AK-3465-01A | 0.842105 | KIRC |
| TCGA-B8-5164-01A | 2.289474 | KIRC |
| TCGA-B0-5713-01A | 2.868421 | KIRC |
| TCGA-BP-5194-01A | 0.710526 | KIRC |
| TCGA-BP-4998-01A | 1 | KIRC |
| TCGA-BP-4995-01A | 1.289474 | KIRC |
| TCGA-A3-3316-01A | 1.210526 | KIRC |
| TCGA-CJ-4913-01A | 1.342105 | KIRC |
| TCGA-MM-A564-01A | 3.026316 | KIRC |
| TCGA-CJ-5671-01A | 1.736842 | KIRC |
| TCGA-A3-3319-01A | 2.157895 | KIRC |
| TCGA-B8-4151-01A | 1.631579 | KIRC |
| TCGA-BP-5202-01A | 1.105263 | KIRC |
| TCGA-MM-A563-01A | 1.394737 | KIRC |
| TCGA-CJ-5682-01A | 2.184211 | KIRC |
| TCGA-CZ-5466-01A | 1.921053 | KIRC |
| TCGA-B8-A54E-01A | 1.342105 | KIRC |
| TCGA-BP-5180-01A | 1.315789 | KIRC |
| TCGA-B8-5163-01A | 1.973684 | KIRC |
| TCGA-CZ-5451-01A | 2.368421 | KIRC |
| TCGA-B8-4148-01A | 0.973684 | KIRC |
| TCGA-B0-5081-01A | 0.684211 | KIRC |
| TCGA-CJ-4923-01A | 1.263158 | KIRC |
| TCGA-BP-5004-01A | 1.552632 | KIRC |
| TCGA-A3-3357-01A | 2.842105 | KIRC |
| TCGA-BP-4967-01A | 2.052632 | KIRC |
| TCGA-A3-3326-01A | 1.736842 | KIRC |
| TCGA-BP-4970-01A | 0.789474 | KIRC |
| TCGA-CZ-5469-01A | 1.421053 | KIRC |
| TCGA-B0-5402-01A | 1.394737 | KIRC |
| TCGA-CJ-6033-01A | 2.263158 | KIRC |
| TCGA-CZ-5465-01A | 3.236842 | KIRC |
| TCGA-CW-5591-01A | 1.052632 | KIRC |
| TCGA-B0-4700-01A | 0.394737 | KIRC |
| TCGA-CZ-5468-01A | 3.552632 | KIRC |
| TCGA-CJ-6027-01A | 2.447368 | KIRC |
| TCGA-B2-5636-01A | 0.552632 | KIRC |
| TCGA-BP-5192-01A | 1.657895 | KIRC |
| TCGA-CZ-5456-01A | 1.868421 | KIRC |
| TCGA-BP-4770-01A | 1.552632 | KIRC |
| TCGA-CJ-5683-01A | 1.894737 | KIRC |
| TCGA-CZ-5461-01A | 1.894737 | KIRC |
| TCGA-B8-5162-01A | 1.710526 | KIRC |
| TCGA-G6-A8L8-01A | 2.421053 | KIRC |
| TCGA-B0-5399-01A | 1.131579 | KIRC |
| TCGA-B2-5639-01A | 1.736842 | KIRC |
| TCGA-B8-5551-01A | 1.078947 | KIRC |
| TCGA-A3-3367-01A | 2.052632 | KIRC |
| TCGA-A3-3370-01A | 1.131579 | KIRC |
| TCGA-B4-5843-01A | 1.289474 | KIRC |
| TCGA-G6-A8L6-01A | 2.026316 | KIRC |
| TCGA-CZ-5459-01A | 2.789474 | KIRC |
| TCGA-B2-5635-01A | 1.736842 | KIRC |
| TCGA-B8-4621-01A | 2.552632 | KIRC |
| TCGA-B0-5088-01A | 1.578947 | KIRC |
| TCGA-DV-5573-01A | 0.842105 | KIRC |
| TCGA-B0-5085-01A | 2 | KIRC |
| TCGA-CZ-5455-01A | 1.236842 | KIRC |
| TCGA-CZ-5458-01A | 0.921053 | KIRC |
| TCGA-CJ-6032-01A | 1.421053 | KIRC |
| TCGA-B0-5077-01A | 1.5 | KIRC |
| TCGA-BP-5190-01A | 1.263158 | KIRC |
| TCGA-BP-5183-01A | 1.5 | KIRC |
| TCGA-CZ-4866-01A | 2.5 | KIRC |
| TCGA-MW-A4EC-01A | 1.421053 | KIRC |
| TCGA-A3-A6NJ-01A | 1.421053 | KIRC |
| TCGA-AS-3777-01A | 0.578947 | KIRC |
| TCGA-B8-A54D-01A | 1.789474 | KIRC |
| TCGA-CZ-4865-01A | 2.578947 | KIRC |
| TCGA-B8-A54J-01A | 2.078947 | KIRC |
| TCGA-CJ-4920-01A | 4.131579 | KIRC |
| TCGA-BP-4973-01A | 0.842105 | KIRC |
| TCGA-A3-3372-01A | 1.763158 | KIRC |
| TCGA-B0-5117-01A | 0.315789 | KIRC |
| TCGA-B8-A54F-01A | 0.894737 | KIRC |
| TCGA-CJ-6030-01A | 3.105263 | KIRC |
| TCGA-CW-5581-01A | 1.552632 | KIRC |
| TCGA-CW-5589-01A | 1.5 | KIRC |
| TCGA-B0-5096-01A | 2.631579 | KIRC |
| TCGA-BP-5182-01A | 2.078947 | KIRC |
| TCGA-B8-4153-01B | 1.842105 | KIRC |
| TCGA-CJ-5680-01A | 1.657895 | KIRC |
| TCGA-CZ-4856-01A | 1.473684 | KIRC |
| TCGA-A3-A8OV-01A | 4.868421 | KIRC |
| TCGA-CW-5588-01A | 1.315789 | KIRC |
| TCGA-CW-5585-01A | 1.526316 | KIRC |
| TCGA-B0-5120-01A | 1.710526 | KIRC |
| TCGA-BP-4972-01A | 1.263158 | KIRC |
| TCGA-B0-5099-01A | 2.105263 | KIRC |
| TCGA-A3-3373-01A | 1.842105 | KIRC |
| TCGA-BP-5169-01A | 1.5 | KIRC |
| TCGA-BP-5007-01A | 0.736842 | KIRC |
| TCGA-DV-A4VZ-01A | 0.342105 | KIRC |
| TCGA-B0-5098-01A | 21.15789 | KIRC |
| TCGA-DV-5567-01A | 0.368421 | KIRC |
| TCGA-B0-5095-01A | 2.210526 | KIRC |
| TCGA-CZ-4859-01A | 2.973684 | KIRC |
| TCGA-B0-5400-01A | 0.842105 | KIRC |
| TCGA-BP-5168-01A | 3.684211 | KIRC |
| TCGA-BP-4964-01A | 2.078947 | KIRC |
| TCGA-CZ-5984-01A | 1.236842 | KIRC |
| TCGA-A3-3317-01A | 2.421053 | KIRC |
| TCGA-BP-5181-01A | 1.657895 | KIRC |
| TCGA-A3-3378-01A | 2 | KIRC |
| TCGA-CZ-4853-01A | 3.421053 | KIRC |
| TCGA-B0-5104-01A | 1.868421 | KIRC |
| TCGA-CW-5583-01A | 0.736842 | KIRC |
| TCGA-A3-3376-01A | 1.289474 | KIRC |
| TCGA-B8-A54K-01A | 0.736842 | KIRC |
| TCGA-BP-4971-01A | 1.236842 | KIRC |
| TCGA-BP-5186-01A | 0.921053 | KIRC |
| TCGA-CJ-5677-01A | 1.921053 | KIRC |
| TCGA-B0-5092-01A | 1.421053 | KIRC |
| TCGA-CZ-5460-01A | 1.921053 | KIRC |
| TCGA-CW-6093-01A | 3.263158 | KIRC |
| TCGA-B0-4827-01A | 2.447368 | KIRC |
| TCGA-B0-5694-01A | 1.947368 | KIRC |
| TCGA-BP-4975-01A | 0.5 | KIRC |
| TCGA-BP-5174-01A | 1.157895 | KIRC |
| TCGA-CJ-4904-01A | 1.052632 | KIRC |
| TCGA-CZ-4863-01A | 1.131579 | KIRC |
| TCGA-EU-5904-01A | 1.078947 | KIRC |
| TCGA-A3-3346-01A | 2.605263 | KIRC |
| TCGA-B8-A54G-01A | 1.263158 | KIRC |
| TCGA-BP-4976-01A | 2.789474 | KIRC |
| TCGA-A3-3320-01A | 2.421053 | KIRC |
| TCGA-BP-5189-01A | 1.342105 | KIRC |
| TCGA-B4-5377-01A | 1.447368 | KIRC |
| TCGA-AK-3440-01A | 0.605263 | KIRC |
| TCGA-BP-5185-01A | 2.368421 | KIRC |
| TCGA-B8-5550-01A | 3 | KIRC |
| TCGA-CJ-5686-01A | 1.921053 | KIRC |
| TCGA-BP-5177-01A | 1.368421 | KIRC |
| TCGA-B0-5707-01A | 1.184211 | KIRC |
| TCGA-B4-5834-01A | 1 | KIRC |
| TCGA-CZ-5453-01A | 2.315789 | KIRC |
| TCGA-B2-5633-01A | 1.578947 | KIRC |
| TCGA-EU-5907-01A | 1.289474 | KIRC |
| TCGA-B0-5083-01A | 0.236842 | KIRC |
| TCGA-CJ-4907-01A | 1.578947 | KIRC |
| TCGA-CZ-5462-01A | 1.894737 | KIRC |
| TCGA-DV-5575-01A | 0.684211 | KIRC |
| TCGA-B0-5697-01A | 1.631579 | KIRC |
| TCGA-BP-5191-01A | 1.657895 | KIRC |
| TCGA-A3-A6NL-01A | 1.342105 | KIRC |
| TCGA-CJ-6031-01A | 1.842105 | KIRC |
| TCGA-BP-4987-01A | 0.710526 | KIRC |
| TCGA-A3-A8OU-01A | 2.289474 | KIRC |
| TCGA-CJ-5689-01A | 2.105263 | KIRC |
| TCGA-B8-5158-01A | 1.289474 | KIRC |
| TCGA-CW-5580-01A | 2.684211 | KIRC |
| TCGA-A3-3322-01A | 1.552632 | KIRC |
| TCGA-B8-4622-01A | 1.684211 | KIRC |
| TCGA-B8-5159-01A | 1.552632 | KIRC |
| TCGA-DV-5576-01A | 0.026316 | KIRC |
| TCGA-BP-5196-01A | 1.421053 | KIRC |
| TCGA-CW-6090-01A | 3.368421 | KIRC |
| TCGA-CZ-5463-01A | 1.552632 | KIRC |
| TCGA-B0-5121-01A | 1.342105 | KIRC |
| TCGA-CZ-5452-01A | 0.894737 | KIRC |
| TCGA-CZ-5987-01A | 1.605263 | KIRC |
| TCGA-CJ-5681-01A | 0.552632 | KIRC |
| TCGA-B8-4143-01A | 1.894737 | KIRC |
| TCGA-A3-3323-01A | 1.289474 | KIRC |
| TCGA-BP-5199-01A | 2.342105 | KIRC |
| TCGA-G6-A5PC-01A | 1.921053 | KIRC |
| TCGA-A3-3387-01A | 2.078947 | KIRC |
| TCGA-B8-5165-01A | 0.526316 | KIRC |
| TCGA-AK-3443-01A | 0.236842 | KIRC |
| TCGA-B0-5107-01A | 1.842105 | KIRC |
| TCGA-BP-5195-01A | 2 | KIRC |
| TCGA-B8-5553-01A | 1.421053 | KIRC |
| TCGA-BP-5198-01A | 2.236842 | KIRC |
| TCGA-BP-5008-01A | 0.973684 | KIRC |
| TCGA-CJ-5672-01A | 2.842105 | KIRC |
| TCGA-CJ-4900-01A | 1.394737 | KIRC |
| TCGA-B0-5097-01A | 1.684211 | KIRC |
| TCGA-DV-5568-01A | 0.473684 | KIRC |
| TCGA-DV-5565-01A | 1.789474 | KIRC |
| TCGA-BP-5170-01A | 1.368421 | KIRC |
| TCGA-B0-5700-01A | 2.131579 | KIRC |
| TCGA-BP-5009-01A | 1.210526 | KIRC |
| TCGA-B4-5832-01A | 1.684211 | KIRC |
| TCGA-B8-A7U6-01A | 1.447368 | KIRC |
| TCGA-B0-5690-01A | 1.631579 | KIRC |
| TCGA-DV-5569-01A | 0.289474 | KIRC |
| TCGA-CJ-4916-01A | 1.368421 | KIRC |
| TCGA-A3-3313-01A | 2.789474 | KIRC |
| TCGA-CJ-4918-01A | 2.131579 | KIRC |
| TCGA-CW-5587-01A | 1.815789 | KIRC |
| TCGA-BP-5201-01A | 1.210526 | KIRC |
| TCGA-BP-5010-01A | 1.973684 | KIRC |
| TCGA-B2-A4SR-01A | 1.289474 | KIRC |
| TCGA-A3-A8OX-01A | 0.236842 | KIRC |
| TCGA-BP-4993-01A | 1.473684 | KIRC |
| TCGA-AK-3444-01A | 2.684211 | KIRC |
| TCGA-B2-5641-01A | 1.921053 | KIRC |
| TCGA-DV-5566-01A | 1.578947 | KIRC |
| TCGA-BP-5006-01A | 1.105263 | KIRC |
| TCGA-A3-A8CQ-01A | 1.5 | KIRC |
| TCGA-B0-4823-01A | 3.236842 | KIRC |
| TCGA-B0-5711-01A | 0.894737 | KIRC |
| TCGA-B0-5119-01A | 2.631579 | KIRC |
| TCGA-CW-6097-01A | 0.342105 | KIRC |
| TCGA-B0-5084-01A | 1.447368 | KIRC |
| TCGA-B8-A54H-01A | 2.368421 | KIRC |
| TCGA-CZ-5454-01A | 0.868421 | KIRC |
| TCGA-B0-5115-01A | 1.578947 | KIRC |
| TCGA-A3-3380-01A | 0.947368 | KIRC |
| TCGA-BP-4992-01A | 1.105263 | KIRC |
| TCGA-B0-5116-01A | 1.842105 | KIRC |
| TCGA-AS-3778-01A | 1.763158 | KIRC |
| TCGA-BP-5001-01A | 0.973684 | KIRC |
| TCGA-6D-AA2E-01A | 0.815789 | KIRC |
| TCGA-CJ-4899-01A | 0.921053 | KIRC |
| TCGA-B0-5075-01A | 2.736842 | KIRC |
| TCGA-AK-3453-01A | 0.368421 | KIRC |
| TCGA-B0-5100-01A | 0.973684 | KIRC |
| TCGA-CZ-4864-01A | 2.526316 | KIRC |
| TCGA-BP-4782-01A | 2.184211 | KIRC |
| TCGA-CJ-4903-01A | 1.368421 | KIRC |
| TCGA-B8-4146-01B | 1.131579 | KIRC |
| TCGA-BP-5173-01A | 2.236842 | KIRC |
| TCGA-B0-5703-01A | 2.315789 | KIRC |
| TCGA-CZ-5457-01A | 2.157895 | KIRC |
| TCGA-B0-5693-01A | 1.078947 | KIRC |
| TCGA-CZ-5982-01A | 1.342105 | KIRC |
| TCGA-B8-A54I-01A | 1.657895 | KIRC |
| TCGA-A3-3365-01A | 1.131579 | KIRC |
| TCGA-A3-A6NI-01A | 2.631579 | KIRC |
| TCGA-BP-4801-01A | 1.631579 | KIRC |
| TCGA-A3-3382-01A | 2.973684 | KIRC |
| TCGA-G6-A8L7-01A | 2.5 | KIRC |
| TCGA-BP-4983-01A | 1.710526 | KIRC |
| TCGA-B0-5102-01A | 1.342105 | KIRC |
| TCGA-CJ-6028-01A | 1.184211 | KIRC |
| TCGA-BP-4961-01A | 1.052632 | KIRC |
| TCGA-B2-4101-01A | 1.105263 | KIRC |
| TCGA-A3-3308-01A | 2.394737 | KIRC |
| TCGA-B0-5702-01A | 1.315789 | KIRC |
| TCGA-DV-A4VX-01A | 2.605263 | KIRC |
| TCGA-B0-4945-01A | 1.131579 | KIRC |
| TCGA-CZ-5470-01A | 1.394737 | KIRC |
| TCGA-CJ-4902-01A | 1.736842 | KIRC |
| TCGA-CW-6087-01A | 1.394737 | KIRC |
| TCGA-CZ-5467-01A | 1.736842 | KIRC |
| TCGA-B0-5094-01A | 2.394737 | KIRC |
| TCGA-A3-3358-01A | 1.921053 | KIRC |
| TCGA-A3-A8OW-01A | 0.552632 | KIRC |
| TCGA-B0-5692-01A | 2.210526 | KIRC |
| TCGA-BP-4968-01A | 1.184211 | KIRC |
| TCGA-BP-4965-01A | 1.552632 | KIRC |
| TCGA-A3-3383-01A | 1.657895 | KIRC |
| TCGA-BP-4982-01A | 1.052632 | KIRC |
| TCGA-3Z-A93Z-01A | 2.947368 | KIRC |
| TCGA-B8-A8YJ-01A | 0.052632 | KIRC |
| TCGA-AK-3447-01A | 0.631579 | KIRC |
| TCGA-P4-AAVM-01A | 1.921053 | KIRP |
| TCGA-BQ-7051-01A | 2.815789 | KIRP |
| TCGA-SX-A71R-01A | 1.526316 | KIRP |
| TCGA-G7-6795-01A | 1.921053 | KIRP |
| TCGA-BQ-5892-01A | 1.736842 | KIRP |
| TCGA-MH-A562-01A | 1.947368 | KIRP |
| TCGA-HE-7129-01A | 1.315789 | KIRP |
| TCGA-A4-7996-01A | 2.5 | KIRP |
| TCGA-GL-A59T-01A | 2.789474 | KIRP |
| TCGA-5P-A9JY-01A | 4.105263 | KIRP |
| TCGA-G7-6796-01A | 1.605263 | KIRP |
| TCGA-F9-A7VF-01A | 2.631579 | KIRP |
| TCGA-HE-7128-01A | 1.131579 | KIRP |
| TCGA-2Z-A9JE-01A | 3.131579 | KIRP |
| TCGA-5P-A9JU-01A | 2.5 | KIRP |
| TCGA-B1-A47O-01A | 1.131579 | KIRP |
| TCGA-P4-A5E8-01A | 1.421053 | KIRP |
| TCGA-SX-A7SS-01A | 3.763158 | KIRP |
| TCGA-B9-4115-01A | 1.657895 | KIRP |
| TCGA-V9-A7HT-01A | 2.578947 | KIRP |
| TCGA-BQ-7061-01A | 2.5 | KIRP |
| TCGA-BQ-5880-01A | 1.263158 | KIRP |
| TCGA-BQ-5893-01A | 1.394737 | KIRP |
| TCGA-BQ-7056-01A | 1.052632 | KIRP |
| TCGA-MH-A856-01A | 2.421053 | KIRP |
| TCGA-GL-7966-01A | 0.684211 | KIRP |
| TCGA-A4-A5DU-01A | 1.026316 | KIRP |
| TCGA-DW-7836-01A | 1.368421 | KIRP |
| TCGA-BQ-7055-01A | 0.210526 | KIRP |
| TCGA-BQ-7058-01A | 2.210526 | KIRP |
| TCGA-MH-A855-01A | 0.894737 | KIRP |
| TCGA-DW-7838-01A | 2.5 | KIRP |
| TCGA-A4-7915-01A | 0.710526 | KIRP |
| TCGA-HE-A5NK-01A | 2.921053 | KIRP |
| TCGA-UZ-A9PM-01A | 1.868421 | KIRP |
| TCGA-BQ-7059-01A | 2.131579 | KIRP |
| TCGA-B9-4116-01A | 2.578947 | KIRP |
| TCGA-P4-A5E6-01A | 2.842105 | KIRP |
| TCGA-B3-4104-01A | 2.289474 | KIRP |
| TCGA-DW-7839-01A | 1.473684 | KIRP |
| TCGA-2K-A9WE-01A | 1 | KIRP |
| TCGA-A4-A4ZT-01A | 1.657895 | KIRP |
| TCGA-Y8-A8RZ-01A | 2.184211 | KIRP |
| TCGA-B3-8121-01A | 1 | KIRP |
| TCGA-GL-A59R-01A | 3.131579 | KIRP |
| TCGA-G7-6789-01A | 0.815789 | KIRP |
| TCGA-2Z-A9JD-01A | 3.868421 | KIRP |
| TCGA-2Z-A9JJ-01A | 0.473684 | KIRP |
| TCGA-F9-A8NY-01A | 1.447368 | KIRP |
| TCGA-MH-A55Z-01A | 3.157895 | KIRP |
| TCGA-AL-3466-01A | 1 | KIRP |
| TCGA-BQ-5882-01A | 1.842105 | KIRP |
| TCGA-UZ-A9Q1-01A | 3.026316 | KIRP |
| TCGA-SX-A7SO-01A | 1.5 | KIRP |
| TCGA-A4-8630-01A | 1.710526 | KIRP |
| TCGA-UZ-A9PN-01A | 2.289474 | KIRP |
| TCGA-5P-A9KE-01A | 2.710526 | KIRP |
| TCGA-AL-3468-01A | 2.631579 | KIRP |
| TCGA-KV-A74V-01A | 0.815789 | KIRP |
| TCGA-G7-7502-01A | 2.026316 | KIRP |
| TCGA-IA-A40Y-01A | 1.684211 | KIRP |
| TCGA-UZ-A9PU-01A | 1.842105 | KIRP |
| TCGA-B9-A69E-01A | 3.105263 | KIRP |
| TCGA-HE-A5NL-01A | 2.342105 | KIRP |
| TCGA-BQ-5890-01A | 2.289474 | KIRP |
| TCGA-GL-8500-01A | 2.763158 | KIRP |
| TCGA-BQ-5883-01A | 0.210526 | KIRP |
| TCGA-IA-A40U-01A | 1.526316 | KIRP |
| TCGA-IA-A83W-01A | 2.578947 | KIRP |
| TCGA-MH-A560-01A | 1.631579 | KIRP |
| TCGA-A4-7583-01A | 1.868421 | KIRP |
| TCGA-2Z-A9JP-01A | 2.157895 | KIRP |
| TCGA-A4-7287-01A | 1.210526 | KIRP |
| TCGA-B1-7332-01A | 1.421053 | KIRP |
| TCGA-A4-7584-01A | 2.421053 | KIRP |
| TCGA-HE-A5NH-01A | 2.921053 | KIRP |
| TCGA-BQ-5884-01A | 1.263158 | KIRP |
| TCGA-Y8-A897-01A | 2.394737 | KIRP |
| TCGA-DW-5560-01A | 0.973684 | KIRP |
| TCGA-SX-A7SL-01A | 2.973684 | KIRP |
| TCGA-BQ-5876-01A | 2.315789 | KIRP |
| TCGA-2Z-A9J5-01A | 3.210526 | KIRP |
| TCGA-A4-A5Y0-01A | 2.026316 | KIRP |
| TCGA-2Z-A9J8-01A | 1.052632 | KIRP |
| TCGA-SX-A71V-01A | 3.842105 | KIRP |
| TCGA-DW-7840-01A | 2.526316 | KIRP |
| TCGA-SX-A7SR-01A | 2.842105 | KIRP |
| TCGA-B1-A654-01A | 2.105263 | KIRP |
| TCGA-2Z-A9J6-01A | 1.763158 | KIRP |
| TCGA-BQ-5875-01A | 2.105263 | KIRP |
| TCGA-BQ-5878-01A | 2.710526 | KIRP |
| TCGA-J7-6720-01A | 1.078947 | KIRP |
| TCGA-A4-8310-01A | 2.026316 | KIRP |
| TCGA-BQ-5879-01A | 0.578947 | KIRP |
| TCGA-B9-5155-01A | 3 | KIRP |
| TCGA-5P-A9JW-01A | 2.710526 | KIRP |
| TCGA-UZ-A9PX-01A | 3.736842 | KIRP |
| TCGA-G7-A8LD-01A | 1.473684 | KIRP |
| TCGA-A4-A48D-01A | 3.263158 | KIRP |
| TCGA-B3-3926-01A | 0.5 | KIRP |
| TCGA-IA-A40X-01A | 0.736842 | KIRP |
| TCGA-J7-A8I2-01A | 2.026316 | KIRP |
| TCGA-B3-3925-01A | 2.263158 | KIRP |
| TCGA-EV-5901-01A | 1.263158 | KIRP |
| TCGA-2Z-A9J1-01A | 3.552632 | KIRP |
| TCGA-2Z-A9JM-01A | 2 | KIRP |
| TCGA-B9-5156-01A | 2.5 | KIRP |
| TCGA-SX-A71S-01A | 5.052632 | KIRP |
| TCGA-GL-A9DE-01A | 3.710526 | KIRP |
| TCGA-A4-8517-01A | 2.447368 | KIRP |
| TCGA-G7-A8LB-01A | 3.763158 | KIRP |
| TCGA-PJ-A5Z8-01A | 2.289474 | KIRP |
| TCGA-5P-A9K8-01A | 0.5 | KIRP |
| TCGA-UZ-A9PJ-01A | 2.157895 | KIRP |
| TCGA-DZ-6131-01A | 0.842105 | KIRP |
| TCGA-BQ-5894-01A | 0.710526 | KIRP |
| TCGA-PJ-A5Z9-01A | 1.973684 | KIRP |
| TCGA-AL-7173-01A | 1.736842 | KIRP |
| TCGA-SX-A7SQ-01A | 3.236842 | KIRP |
| TCGA-GL-A4EM-01A | 2.026316 | KIRP |
| TCGA-J7-8537-01A | 1.315789 | KIRP |
| TCGA-B3-4103-01A | 1.552632 | KIRP |
| TCGA-A4-7828-01A | 0.815789 | KIRP |
| TCGA-2Z-A9JN-01A | 0.552632 | KIRP |
| TCGA-5P-A9K6-01A | 1.815789 | KIRP |
| TCGA-Y8-A894-01A | 3.210526 | KIRP |
| TCGA-B1-5398-01A | 3.236842 | KIRP |
| TCGA-B9-A5W7-01A | 0.631579 | KIRP |
| TCGA-P4-A5ED-01A | 0.289474 | KIRP |
| TCGA-HE-A5NI-01A | 3.815789 | KIRP |
| TCGA-Y8-A8S0-01A | 1.315789 | KIRP |
| TCGA-A4-A5XZ-01A | 2.394737 | KIRP |
| TCGA-BQ-7046-01A | 1.131579 | KIRP |
| TCGA-BQ-5887-01A | 0.394737 | KIRP |
| TCGA-GL-A9DD-01A | 3.447368 | KIRP |
| TCGA-DZ-6135-01A | 1 | KIRP |
| TCGA-BQ-7048-01A | 1.947368 | KIRP |
| TCGA-B9-7268-01A | 1.605263 | KIRP |
| TCGA-BQ-7045-01A | 2.526316 | KIRP |
| TCGA-G7-A8LE-01A | 3.078947 | KIRP |
| TCGA-O9-A75Z-01A | 0.605263 | KIRP |
| TCGA-DW-7842-01A | 0.684211 | KIRP |
| TCGA-BQ-7049-01A | 0.368421 | KIRP |
| TCGA-AL-A5DJ-01A | 1.052632 | KIRP |
| TCGA-B1-A657-01A | 2.631579 | KIRP |
| TCGA-A4-8312-01A | 0.368421 | KIRP |
| TCGA-B9-4617-01A | 1.631579 | KIRP |
| TCGA-P4-A5EB-01A | 5.105263 | KIRP |
| TCGA-UZ-A9PP-01A | 2.631579 | KIRP |
| TCGA-DW-5561-01A | 1.289474 | KIRP |
| TCGA-A4-A5Y1-01A | 2.605263 | KIRP |
| TCGA-F9-A97G-01A | 2.210526 | KIRP |
| TCGA-5P-A9KC-01A | 2.657895 | KIRP |
| TCGA-DZ-6133-01A | 2.894737 | KIRP |
| TCGA-A4-7288-01A | 2.184211 | KIRP |
| TCGA-UZ-A9PK-01A | 2.710526 | KIRP |
| TCGA-2Z-A9JO-01A | 1.236842 | KIRP |
| TCGA-B9-A8YI-01A | 4.157895 | KIRP |
| TCGA-A4-8311-01A | 1.526316 | KIRP |
| TCGA-A4-7286-01A | 1.210526 | KIRP |
| TCGA-HE-7130-01A | 5.078947 | KIRP |
| TCGA-DW-7841-01A | 1.421053 | KIRP |
| TCGA-WN-AB4C-01A | 3.5 | KIRP |
| TCGA-5P-A9K2-01A | 2.342105 | KIRP |
| TCGA-MH-A854-01A | 4.342105 | KIRP |
| TCGA-GL-6846-01A | 2.105263 | KIRP |
| TCGA-DW-7834-01A | 1.763158 | KIRP |
| TCGA-DZ-6132-01A | 3.657895 | KIRP |
| TCGA-SX-A71U-01A | 3.368421 | KIRP |
| TCGA-AL-3467-01A | 0.342105 | KIRP |
| TCGA-MH-A55W-01A | 2.710526 | KIRP |
| TCGA-SX-A7SP-01A | 2.605263 | KIRP |
| TCGA-B9-4114-01A | 2.263158 | KIRP |
| TCGA-KV-A6GE-01A | 1.684211 | KIRP |
| TCGA-5P-A9K3-01A | 2.078947 | KIRP |
| TCGA-5P-A9KA-01A | 2.763158 | KIRP |
| TCGA-GL-7773-01A | 3.657895 | KIRP |
| TCGA-IA-A83S-01A | 4.289474 | KIRP |
| TCGA-F9-A4JJ-01A | 0.894737 | KIRP |
| TCGA-UZ-A9PR-01A | 3.789474 | KIRP |
| TCGA-A4-8098-01A | 2.578947 | KIRP |
| TCGA-AT-A5NU-01A | 2.052632 | KIRP |
| TCGA-MH-A857-01A | 0.763158 | KIRP |
| TCGA-AL-3472-01A | 1.605263 | KIRP |
| TCGA-DW-7837-01A | 0.763158 | KIRP |
| TCGA-Q2-A5QZ-01A | 2.210526 | KIRP |
| TCGA-B3-A6W5-01A | 1.236842 | KIRP |
| TCGA-4A-A93Y-01A | 0.078947 | KIRP |
| TCGA-2Z-A9JI-01A | 0.210526 | KIRP |
| TCGA-EV-5902-01A | 2.394737 | KIRP |
| TCGA-A4-7734-01A | 1.552632 | KIRP |
| TCGA-2Z-A9J2-01A | 2.526316 | KIRP |
| TCGA-P4-A5E7-01A | 3.368421 | KIRP |
| TCGA-B9-4117-01A | 1.289474 | KIRP |
| TCGA-UZ-A9PL-01A | 2.552632 | KIRP |
| TCGA-Y8-A8S1-01A | 1.526316 | KIRP |
| TCGA-KV-A6GD-01A | 1.684211 | KIRP |
| TCGA-P4-AAVL-01A | 2.657895 | KIRP |
| TCGA-IA-A83V-01A | 2.105263 | KIRP |
| TCGA-A4-A57E-01A | 2.631579 | KIRP |
| TCGA-AL-3473-01A | 1.394737 | KIRP |
| TCGA-B9-A8YH-01A | 3.552632 | KIRP |
| TCGA-A4-7997-01A | 2.210526 | KIRP |
| TCGA-EV-5903-01A | 3.105263 | KIRP |
| TCGA-2Z-A9J3-01A | 4.421053 | KIRP |
| TCGA-G7-6797-01A | 1.894737 | KIRP |
| TCGA-A4-A6HP-01A | 3.078947 | KIRP |
| TCGA-IZ-A6M8-01A | 3.184211 | KIRP |
| TCGA-5P-A9K0-01A | 3.342105 | KIRP |
| TCGA-DW-7963-01B | 0.815789 | KIRP |
| TCGA-5P-A9KH-01A | 0.631579 | KIRP |
| TCGA-IZ-A6M9-01A | 2.157895 | KIRP |
| TCGA-2Z-A9JS-01A | 4.157895 | KIRP |
| TCGA-B1-A655-01A | 3.052632 | KIRP |
| TCGA-IZ-8196-01A | 2.605263 | KIRP |
| TCGA-2Z-A9JK-01A | 2.868421 | KIRP |
| TCGA-SX-A7SN-01A | 3.710526 | KIRP |
| TCGA-BQ-7050-01A | 0.552632 | KIRP |
| TCGA-BQ-5886-01A | 1.710526 | KIRP |
| TCGA-2Z-A9JQ-01A | 3.157895 | KIRP |
| TCGA-Y8-A8RY-01A | 2.394737 | KIRP |
| TCGA-F9-A7Q0-01A | 0.526316 | KIRP |
| TCGA-WN-A9G9-01A | 1.342105 | KIRP |
| TCGA-SX-A71W-01A | 1.157895 | KIRP |
| TCGA-A4-7585-01A | 2.105263 | KIRP |
| TCGA-BQ-5885-01A | 3.184211 | KIRP |
| TCGA-BQ-5888-01A | 0.552632 | KIRP |
| TCGA-B9-A5W9-01A | 2 | KIRP |
| TCGA-B1-A656-01A | 4.052632 | KIRP |
| TCGA-IZ-8195-01A | 2.131579 | KIRP |
| TCGA-BQ-5889-01A | 0.789474 | KIRP |
| TCGA-A4-7732-01A | 1.447368 | KIRP |
| TCGA-B9-A5W8-01A | 1.605263 | KIRP |
| TCGA-UZ-A9PO-01A | 3.421053 | KIRP |
| TCGA-2Z-A9JT-01A | 2.710526 | KIRP |
| TCGA-4A-A93X-01A | 3.342105 | KIRP |
| TCGA-IA-A83T-01A | 1.894737 | KIRP |
| TCGA-G7-A8LC-01A | 0.368421 | KIRP |
| TCGA-BQ-5881-01A | 1.078947 | KIRP |
| TCGA-5P-A9JV-01A | 1.736842 | KIRP |
| TCGA-2Z-A9JG-01A | 3.078947 | KIRP |
| TCGA-BQ-7060-01A | 1.157895 | KIRP |
| TCGA-B9-A44B-01A | 4.552632 | KIRP |
| TCGA-G7-6790-01A | 2.342105 | KIRP |
| TCGA-SX-A7SU-01A | 1.421053 | KIRP |
| TCGA-G7-7501-01A | 1.026316 | KIRP |
| TCGA-B1-A47M-01A | 3.421053 | KIRP |
| TCGA-UZ-A9PZ-01A | 1.710526 | KIRP |
| TCGA-A4-8515-01A | 1.605263 | KIRP |
| TCGA-UZ-A9Q0-01A | 2.131579 | KIRP |
| TCGA-5P-A9JZ-01A | 1.631579 | KIRP |
| TCGA-BQ-7062-01A | 1.210526 | KIRP |
| TCGA-PJ-A8JU-01A | 3.394737 | KIRP |
| TCGA-BQ-7053-01A | 1.605263 | KIRP |
| TCGA-A4-A772-01A | 1.394737 | KIRP |
| TCGA-2Z-A9JR-01A | 1.710526 | KIRP |
| TCGA-2Z-A9JL-01A | 3.184211 | KIRP |
| TCGA-G7-6792-01A | 0.947368 | KIRP |
| TCGA-B1-A47N-01A | 0.763158 | KIRP |
| TCGA-UZ-A9PV-01A | 1.947368 | KIRP |
| TCGA-UN-AAZ9-01A | 0.815789 | KIRP |
| TCGA-B9-4113-01A | 2.210526 | KIRP |
| TCGA-P4-A5EA-01A | 1.657895 | KIRP |
| TCGA-MH-A561-01A | 3.315789 | KIRP |
| TCGA-4A-A93W-01A | 3.263158 | KIRP |
| TCGA-BQ-7044-01A | 2.342105 | KIRP |
| TCGA-DZ-6134-01A | 1.026316 | KIRP |
| TCGA-BQ-5891-01A | 0.657895 | KIRP |
| TCGA-G7-A4TM-01A | 1.184211 | KIRP |
| TCGA-A4-A7UZ-01A | 2.236842 | KIRP |
| TCGA-SX-A7SM-01A | 2.657895 | KIRP |
| TCGA-GL-A9DC-01A | 3.894737 | KIRP |
| TCGA-Y8-A896-01A | 3.684211 | KIRP |
| TCGA-BQ-5877-01A | 2.394737 | KIRP |
| TCGA-Y8-A898-01A | 2.315789 | KIRP |
| TCGA-G7-6793-01A | 0.921053 | KIRP |
| TCGA-Y8-A895-01A | 3.710526 | KIRP |
| TCGA-UZ-A9PS-01A | 5.421053 | KIRP |
| TCGA-HE-A5NF-01A | 2.078947 | KIRP |
| TCGA-2Z-A9J7-01A | 1.447368 | KIRP |
| TCGA-HE-A5NJ-01A | 1.973684 | KIRP |
