# Supplementary material for: Pyroptosis-Related lncRNA Prognostic Model for Renal Cancer Contributes to Immunodiagnosis and Immunotherapy
Source: Front Oncol. 2022 Jul 4;12:837155. doi: 10.3389/fonc.2022.837155 (PMC9291251; doi:10.3389/fonc.2022.837155)
Supplement: Supplementary file 8 [file Table_5.docx]

**Supplementary S5** TCR,BCR,Aneuploidy,neoantigen scores of KIRC

| TCGA Participant Barcode | Aneuploidy Score | BCR Richness | TCR Richness | SNV Neoantigens |
| --- | --- | --- | --- | --- |
| TCGA-A3-3319 | 14 | 10 | 30 | 32 |
| TCGA-A3-3323 | 1 | NA | 34 | NA |
| TCGA-A3-3335 | 3 | NA | 22 | NA |
| TCGA-A3-3383 | 1 | NA | 14 | 20 |
| TCGA-A3-A6NJ | 1 | 2 | 26 | 26 |
| TCGA-A3-A8OX | 0 | 12 | 49 | 26 |
| TCGA-AK-3434 | 6 | NA | 14 | NA |
| TCGA-AK-3445 | 9 | NA | 68 | NA |
| TCGA-AK-3454 | 1 | NA | 6 | NA |
| TCGA-B0-4688 | 10 | 15 | 10 | NA |
| TCGA-B0-4691 | 6 | 34 | 71 | NA |
| TCGA-B0-4697 | NA | 147 | 55 | NA |
| TCGA-B0-4706 | 1 | NA | 35 | 21 |
| TCGA-B0-5081 | 0 | 214 | 235 | 21 |
| TCGA-B0-5088 | 1 | NA | 52 | 22 |
| TCGA-B0-5700 | NA | NA | 24 | 25 |
| TCGA-B4-5834 | 3 | NA | 26 | 15 |
| TCGA-B8-4153 | 0 | NA | 13 | 28 |
| TCGA-B8-4619 | 4 | 14 | 4 | 11 |
| TCGA-B8-5162 | 3 | 167 | 129 | 32 |
| TCGA-B8-A54J | 1 | NA | 20 | 29 |
| TCGA-BP-4169 | 1 | 3 | 65 | NA |
| TCGA-BP-4176 | 16 | 2 | 75 | NA |
| TCGA-BP-4325 | 4 | NA | 26 | NA |
| TCGA-BP-4335 | 2 | 34 | 44 | NA |
| TCGA-BP-4341 | 1 | NA | 4 | NA |
| TCGA-BP-4355 | 0 | NA | 12 | NA |
| TCGA-BP-4758 | 2 | 1 | 99 | NA |
| TCGA-BP-4763 | 8 | NA | 19 | NA |
| TCGA-BP-4775 | 14 | 5 | 34 | NA |
| TCGA-BP-4782 | 5 | 1 | 60 | 48 |
| TCGA-BP-4784 | NA | 17 | 3 | NA |
| TCGA-BP-4789 | 3 | NA | 12 | NA |
| TCGA-BP-4963 | 8 | NA | 28 | 41 |
| TCGA-BP-4992 | 14 | NA | 39 | 25 |
| TCGA-BP-4998 | 3 | 6 | 50 | 17 |
| TCGA-BP-5007 | 0 | NA | 39 | 12 |
| TCGA-BP-5169 | 7 | 332 | 52 | 15 |
| TCGA-BP-5175 | 19 | NA | 19 | 17 |
| TCGA-BP-5189 | 3 | 1 | 58 | 28 |
| TCGA-BP-5201 | 7 | NA | 58 | 14 |
| TCGA-CJ-4638 | 2 | NA | 21 | 29 |
| TCGA-CJ-4878 | 3 | NA | 5 | NA |
| TCGA-CJ-4891 | 3 | 4 | 23 | NA |
| TCGA-CJ-4892 | 3 | NA | 71 | NA |
| TCGA-CJ-4893 | 6 | 4 | 71 | NA |
| TCGA-CJ-4897 | 29 | 24 | 45 | NA |
| TCGA-CJ-4920 | 0 | 3 | 24 | 40 |
| TCGA-CJ-5678 | 3 | 4 | 105 | 21 |
| TCGA-CJ-5683 | 6 | NA | 13 | 33 |
| TCGA-CJ-6033 | 4 | 4 | 24 | 40 |
| TCGA-CW-5583 | 3 | 1 | 19 | 11 |
| TCGA-CW-5585 | 3 | NA | 6 | 14 |
| TCGA-CZ-4866 | 25 | 2 | 19 | 32 |
| TCGA-CZ-5454 | 4 | NA | 43 | 11 |
| TCGA-CZ-5463 | 6 | NA | 35 | 27 |
| TCGA-CZ-5465 | 27 | 3 | 18 | 50 |
| TCGA-CZ-5466 | 10 | NA | 37 | 27 |
| TCGA-CZ-5982 | 2 | 1 | 11 | 20 |
| TCGA-CZ-5986 | 6 | NA | 16 | 15 |
| TCGA-DV-5565 | 7 | NA | 12 | 28 |
| TCGA-G6-A8L6 | 1 | NA | 10 | 29 |
| TCGA-T7-A92I | 2 | NA | 14 | 23 |
| TCGA-3Z-A93Z | 7 | 1 | 8 | 29 |
| TCGA-A3-3317 | 7 | NA | 33 | 34 |
| TCGA-A3-3351 | 1 | 2 | 44 | NA |
| TCGA-A3-3365 | NA | NA | 28 | 21 |
| TCGA-A3-3367 | 1 | NA | 34 | 29 |
| TCGA-AK-3427 | 6 | NA | 0 | 5 |
| TCGA-AK-3430 | 15 | NA | NA | NA |
| TCGA-AK-3431 | 1 | NA | 15 | NA |
| TCGA-AK-3433 | 13 | NA | 1 | NA |
| TCGA-B0-4712 | 25 | 3 | 55 | 53 |
| TCGA-B0-4813 | 6 | NA | 0 | NA |
| TCGA-B0-4816 | 2 | NA | 28 | 31 |
| TCGA-B0-4828 | 6 | NA | 29 | NA |
| TCGA-B0-4847 | 0 | 1 | 84 | NA |
| TCGA-B0-4848 | 3 | 10 | 41 | NA |
| TCGA-B0-4945 | 3 | NA | 22 | 27 |
| TCGA-B0-5080 | 2 | 197 | 103 | 34 |
| TCGA-B0-5110 | 1 | NA | 28 | 28 |
| TCGA-B0-5121 | 1 | NA | 60 | 14 |
| TCGA-B0-5400 | 2 | NA | 12 | 14 |
| TCGA-B0-5696 | 6 | NA | 24 | 28 |
| TCGA-B0-5706 | NA | 133 | 22 | 24 |
| TCGA-B2-3923 | 10 | NA | 3 | 10 |
| TCGA-B2-4102 | 0 | 8 | 40 | 25 |
| TCGA-B2-5639 | 3 | 2 | 18 | 18 |
| TCGA-B8-4148 | 2 | 6 | 49 | 17 |
| TCGA-B8-5159 | 5 | NA | 34 | 18 |
| TCGA-B8-A54G | 1 | NA | 29 | 18 |
| TCGA-BP-4327 | 4 | 5 | 17 | NA |
| TCGA-BP-4337 | 15 | 7 | 16 | NA |
| TCGA-BP-4352 | 22 | 3 | 4 | NA |
| TCGA-BP-4354 | 4 | NA | 35 | NA |
| TCGA-BP-4759 | NA | NA | 23 | NA |
| TCGA-BP-4777 | 2 | 11 | 159 | NA |
| TCGA-BP-4804 | NA | 9 | 95 | NA |
| TCGA-BP-4962 | 5 | 14 | 70 | 15 |
| TCGA-BP-4991 | 2 | NA | 10 | 17 |
| TCGA-BP-4994 | 16 | 52 | 34 | 5 |
| TCGA-BP-5004 | 5 | NA | 35 | 19 |
| TCGA-BP-5187 | 4 | NA | 24 | 39 |
| TCGA-BP-5198 | 16 | 85 | 42 | 18 |
| TCGA-CJ-4634 | 2 | NA | 16 | 32 |
| TCGA-CJ-4635 | 22 | NA | 75 | NA |
| TCGA-CJ-4882 | 3 | 51 | 84 | 26 |
| TCGA-CJ-4902 | 1 | 13 | 93 | 26 |
| TCGA-CJ-5672 | 6 | 2 | 26 | 39 |
| TCGA-CZ-4853 | 3 | 4 | 44 | 29 |
| TCGA-CZ-5464 | 14 | 7 | 97 | NA |
| TCGA-CZ-5467 | 4 | 8 | 15 | 18 |
| TCGA-DV-5567 | 0 | NA | 3 | 8 |
| TCGA-DV-5574 | NA | 27 | 39 | NA |
| TCGA-DV-A4VZ | 0 | 1 | 0 | 6 |
| TCGA-EU-5906 | 1 | NA | 26 | 33 |
| TCGA-A3-3370 | 1 | 2 | 62 | 19 |
| TCGA-A3-A6NL | 3 | NA | 10 | 22 |
| TCGA-A3-A8OV | 3 | NA | 3 | 45 |
| TCGA-AK-3429 | NA | 1 | 88 | 14 |
| TCGA-AK-3440 | 14 | NA | 1 | NA |
| TCGA-AK-3458 | 9 | NA | 16 | 24 |
| TCGA-AS-3777 | 12 | NA | 9 | NA |
| TCGA-B0-4698 | 13 | 8 | 110 | 73 |
| TCGA-B0-4699 | 13 | NA | 56 | NA |
| TCGA-B0-4710 | 19 | 39 | 20 | 29 |
| TCGA-B0-4714 | 3 | NA | 60 | NA |
| TCGA-B0-4718 | 2 | 1 | 10 | NA |
| TCGA-B0-4814 | 4 | 40 | 25 | NA |
| TCGA-B0-4817 | 4 | 2 | 6 | NA |
| TCGA-B0-4824 | 2 | NA | 42 | NA |
| TCGA-B0-4842 | 3 | NA | 10 | 21 |
| TCGA-B0-5094 | 12 | NA | 24 | 40 |
| TCGA-B0-5097 | 6 | NA | 59 | 19 |
| TCGA-B0-5100 | 2 | NA | 4 | 13 |
| TCGA-B0-5119 | 2 | NA | 20 | 15 |
| TCGA-B0-5402 | 5 | 8 | 22 | 23 |
| TCGA-B0-5703 | 5 | 1 | 49 | 25 |
| TCGA-B4-5377 | 4 | 1 | 53 | 18 |
| TCGA-B8-5165 | 0 | 5 | 52 | 5 |
| TCGA-B8-A54E | 4 | NA | 6 | 27 |
| TCGA-BP-4163 | 6 | 1 | 37 | 9 |
| TCGA-BP-4164 | 13 | NA | 12 | 19 |
| TCGA-BP-4349 | 0 | NA | 20 | NA |
| TCGA-BP-4351 | 24 | NA | 13 | NA |
| TCGA-BP-4762 | 1 | 5 | 57 | NA |
| TCGA-BP-4769 | NA | NA | 1 | NA |
| TCGA-BP-4787 | 16 | 1 | 25 | NA |
| TCGA-BP-4799 | 25 | 9 | 34 | NA |
| TCGA-BP-4971 | 5 | NA | 83 | 13 |
| TCGA-BP-4973 | 2 | NA | 55 | 17 |
| TCGA-BP-4975 | 1 | 5 | 27 | 7 |
| TCGA-BP-4985 | 22 | 1 | 27 | 42 |
| TCGA-BP-5191 | 14 | 18 | 103 | 19 |
| TCGA-CJ-4872 | NA | 1 | 63 | NA |
| TCGA-CJ-4886 | 1 | 4 | 67 | NA |
| TCGA-CJ-4912 | 8 | 13 | 14 | 36 |
| TCGA-CJ-5682 | 20 | 1 | 13 | 29 |
| TCGA-CJ-6027 | 9 | 5 | 125 | 34 |
| TCGA-CJ-6031 | 2 | 14 | 91 | 28 |
| TCGA-CW-5580 | 3 | 2 | 21 | 44 |
| TCGA-CW-6093 | 2 | NA | 4 | 37 |
| TCGA-CZ-4862 | 2 | NA | 142 | NA |
| TCGA-CZ-5453 | 3 | NA | 1 | 35 |
| TCGA-CZ-5461 | 18 | NA | 118 | 23 |
| TCGA-G6-A8L7 | 9 | NA | 5 | 29 |
| TCGA-G6-A8L8 | 5 | NA | 10 | 29 |
| TCGA-A3-3307 | 1 | NA | 23 | NA |
| TCGA-A3-3311 | 1 | 23 | 49 | 29 |
| TCGA-A3-3329 | NA | NA | 38 | NA |
| TCGA-A3-3331 | 4 | 5 | 47 | 39 |
| TCGA-A3-3346 | 13 | NA | 20 | 29 |
| TCGA-A3-3347 | 9 | 61 | 45 | 21 |
| TCGA-A3-3359 | 1 | NA | 47 | NA |
| TCGA-A3-3380 | 10 | NA | 23 | 16 |
| TCGA-A3-A6NN | 6 | NA | 18 | 23 |
| TCGA-A3-A8OU | 0 | 26 | 22 | 37 |
| TCGA-AK-3456 | 1 | NA | 7 | 10 |
| TCGA-B0-4690 | 11 | 6 | 54 | NA |
| TCGA-B0-4815 | 14 | 294 | 147 | 31 |
| TCGA-B0-4841 | 3 | NA | 3 | NA |
| TCGA-B0-4843 | 2 | NA | 8 | NA |
| TCGA-B0-5098 | 6 | 1 | 4 | 239 |
| TCGA-B0-5691 | 0 | 3 | 15 | 23 |
| TCGA-B0-5705 | 2 | NA | 63 | 35 |
| TCGA-B0-5712 | 3 | 13 | 26 | 48 |
| TCGA-B0-5812 | 0 | NA | 55 | 19 |
| TCGA-B2-5636 | NA | 16 | 9 | 11 |
| TCGA-B2-5641 | 3 | 1 | 87 | 33 |
| TCGA-B8-5164 | 3 | NA | 232 | 27 |
| TCGA-B8-5552 | 1 | 1 | 52 | 14 |
| TCGA-BP-4158 | 6 | NA | 24 | NA |
| TCGA-BP-4159 | 3 | 1 | 11 | NA |
| TCGA-BP-4160 | 3 | 5 | 39 | NA |
| TCGA-BP-4334 | 10 | NA | 0 | NA |
| TCGA-BP-4338 | NA | 26 | 32 | NA |
| TCGA-BP-4343 | 17 | 6 | 58 | NA |
| TCGA-BP-4790 | 2 | 9 | 41 | NA |
| TCGA-BP-4977 | 11 | NA | 51 | 19 |
| TCGA-BP-4982 | 1 | NA | 43 | 23 |
| TCGA-BP-4987 | 4 | NA | 37 | 13 |
| TCGA-BP-5008 | 2 | 1 | 89 | 18 |
| TCGA-BP-5010 | 3 | 1 | 26 | 26 |
| TCGA-BP-5173 | 26 | NA | 45 | 43 |
| TCGA-BP-5176 | 1 | 1 | 34 | 42 |
| TCGA-BP-5180 | 6 | NA | 78 | 14 |
| TCGA-BP-5200 | 24 | 17 | 52 | 20 |
| TCGA-CJ-4640 | 7 | NA | 81 | 36 |
| TCGA-CJ-4873 | 18 | 40 | 58 | NA |
| TCGA-CJ-4901 | 29 | 76 | 186 | 17 |
| TCGA-CJ-4916 | 2 | 30 | 131 | 22 |
| TCGA-CJ-5675 | 3 | NA | 129 | 28 |
| TCGA-CJ-5679 | 22 | 2 | 2 | 35 |
| TCGA-CJ-5686 | 2 | 47 | 94 | 28 |
| TCGA-CW-6087 | NA | 36 | 198 | 38 |
| TCGA-CZ-4858 | 23 | 14 | 72 | NA |
| TCGA-CZ-4859 | 4 | 1 | 16 | 46 |
| TCGA-CZ-4863 | 0 | NA | 165 | 19 |
| TCGA-CZ-5456 | 27 | 84 | 56 | 25 |
| TCGA-CZ-5460 | 2 | 4 | 111 | 24 |
| TCGA-CZ-5462 | 5 | 16 | 36 | 12 |
| TCGA-CZ-5468 | 17 | NA | 26 | 59 |
| TCGA-GK-A6C7 | 3 | NA | 16 | 47 |
| TCGA-MM-A563 | 6 | 17 | 62 | 24 |
| TCGA-MM-A564 | 9 | NA | 4 | 29 |
| TCGA-A3-3324 | 6 | NA | 55 | NA |
| TCGA-A3-3325 | 22 | 16 | 54 | NA |
| TCGA-A3-3328 | NA | NA | 0 | NA |
| TCGA-A3-3357 | 5 | NA | 38 | 22 |
| TCGA-A3-3376 | 0 | 1 | 34 | 34 |
| TCGA-A3-3385 | 7 | NA | 2 | 27 |
| TCGA-AK-3451 | 1 | NA | 17 | 39 |
| TCGA-AK-3461 | NA | NA | 17 | NA |
| TCGA-B0-4810 | 1 | 7 | 85 | 9 |
| TCGA-B0-4819 | 0 | 32 | 135 | NA |
| TCGA-B0-4822 | 25 | 1 | 10 | NA |
| TCGA-B0-4846 | 1 | NA | 74 | NA |
| TCGA-B0-5075 | 5 | NA | 32 | 39 |
| TCGA-B0-5108 | 5 | 13 | 80 | 18 |
| TCGA-B0-5115 | 2 | NA | 57 | 12 |
| TCGA-B0-5711 | 1 | 71 | 64 | 12 |
| TCGA-B4-5836 | 1 | 3 | 29 | 23 |
| TCGA-B4-5838 | 21 | NA | 18 | 29 |
| TCGA-B8-5163 | 12 | NA | NA | 25 |
| TCGA-B8-5545 | 2 | 5 | 44 | 12 |
| TCGA-B8-5549 | 22 | NA | 39 | 18 |
| TCGA-B8-A54I | 18 | 295 | 23 | 26 |
| TCGA-BP-4161 | 1 | 6 | 99 | 23 |
| TCGA-BP-4332 | 3 | NA | 14 | NA |
| TCGA-BP-4765 | 1 | NA | 5 | NA |
| TCGA-BP-4768 | 23 | NA | 26 | NA |
| TCGA-BP-4798 | 12 | 92 | 94 | NA |
| TCGA-BP-4959 | 4 | NA | 56 | NA |
| TCGA-BP-4964 | 0 | NA | 28 | 46 |
| TCGA-BP-5178 | 19 | 5 | 47 | 16 |
| TCGA-BP-5182 | 4 | 15 | 83 | 13 |
| TCGA-BP-5192 | 1 | NA | 13 | 17 |
| TCGA-CJ-4636 | 11 | NA | 26 | 24 |
| TCGA-CJ-4641 | NA | 2 | 146 | 19 |
| TCGA-CJ-4643 | 2 | NA | 48 | 41 |
| TCGA-CJ-4874 | 1 | 1 | 62 | NA |
| TCGA-CJ-4884 | 3 | NA | 89 | NA |
| TCGA-CJ-4899 | 3 | 3 | 28 | 19 |
| TCGA-CJ-4904 | 4 | NA | 46 | 3 |
| TCGA-CJ-4905 | 2 | NA | 25 | 18 |
| TCGA-CJ-4923 | 1 | 38 | 97 | 19 |
| TCGA-CJ-6030 | 6 | 34 | 117 | 36 |
| TCGA-CW-5588 | 20 | 6 | 28 | NA |
| TCGA-CW-6090 | 11 | 11 | 59 | 41 |
| TCGA-CW-6096 | NA | NA | NA | NA |
| TCGA-CZ-5989 | 2 | NA | 37 | 10 |
| TCGA-EU-5904 | 0 | NA | 20 | 19 |
| TCGA-A3-3352 | 9 | NA | 54 | NA |
| TCGA-A3-3363 | 2 | NA | 15 | 23 |
| TCGA-A3-3374 | 28 | NA | 11 | 8 |
| TCGA-A3-A6NI | 3 | NA | 5 | 22 |
| TCGA-A3-A8CQ | 5 | NA | 8 | 13 |
| TCGA-A3-A8OW | 1 | NA | 15 | 22 |
| TCGA-AK-3428 | 0 | NA | 1 | NA |
| TCGA-AK-3465 | 12 | NA | 3 | 13 |
| TCGA-B0-4703 | 4 | NA | 70 | NA |
| TCGA-B0-4707 | 26 | 17 | 7 | NA |
| TCGA-B0-4713 | 6 | 1 | 26 | NA |
| TCGA-B0-4834 | 2 | NA | 1 | NA |
| TCGA-B0-4844 | 16 | 10 | 34 | NA |
| TCGA-B0-4845 | 13 | NA | 25 | NA |
| TCGA-B0-5694 | 5 | 1 | 10 | 14 |
| TCGA-B0-5695 | 1 | 1 | 31 | 33 |
| TCGA-B0-5697 | 5 | NA | 77 | 31 |
| TCGA-B0-5699 | 1 | NA | 32 | 19 |
| TCGA-B0-5709 | 1 | 17 | 165 | 31 |
| TCGA-B0-5710 | 2 | 2 | 35 | 9 |
| TCGA-B2-3924 | 5 | 17 | 106 | NA |
| TCGA-B2-5635 | 2 | NA | 46 | 19 |
| TCGA-B8-4154 | 1 | NA | 39 | 15 |
| TCGA-B8-5550 | 10 | NA | 27 | 49 |
| TCGA-B8-5551 | 28 | 201 | 122 | 27 |
| TCGA-B8-A54D | 1 | NA | 12 | 21 |
| TCGA-B8-A8YJ | 0 | 70 | 29 | 19 |
| TCGA-BP-4329 | 2 | NA | 45 | NA |
| TCGA-BP-4347 | 8 | NA | 39 | NA |
| TCGA-BP-4771 | NA | 8 | 296 | NA |
| TCGA-BP-4801 | 2 | NA | NA | 18 |
| TCGA-BP-4807 | 2 | NA | 18 | NA |
| TCGA-BP-4960 | 11 | 15 | 17 | 21 |
| TCGA-BP-4961 | 1 | 1 | 17 | 13 |
| TCGA-BP-4968 | 0 | NA | 69 | 18 |
| TCGA-BP-4969 | 23 | NA | 11 | NA |
| TCGA-BP-4970 | 1 | 7 | 75 | 4 |
| TCGA-BP-4974 | 2 | 3 | 37 | 21 |
| TCGA-BP-4983 | 3 | 136 | 132 | 37 |
| TCGA-BP-4999 | 4 | NA | 11 | 22 |
| TCGA-BP-5001 | 1 | NA | 33 | 17 |
| TCGA-BP-5174 | 1 | NA | 23 | 13 |
| TCGA-BP-5177 | 5 | 1 | 27 | 18 |
| TCGA-BP-5185 | 20 | NA | 3 | 27 |
| TCGA-CJ-4881 | 11 | 20 | 63 | NA |
| TCGA-CJ-4895 | 3 | 2 | 48 | NA |
| TCGA-CJ-4900 | 2 | 211 | 66 | 9 |
| TCGA-CJ-5676 | 5 | 14 | 33 | 23 |
| TCGA-CJ-5677 | 6 | 4 | 20 | 32 |
| TCGA-CZ-4856 | 3 | 41 | 71 | 33 |
| TCGA-CZ-5458 | 1 | NA | 60 | 13 |
| TCGA-DV-5566 | 3 | NA | 17 | 21 |
| TCGA-DV-5569 | 0 | NA | 35 | NA |
| TCGA-DV-5576 | 2 | NA | 1 | NA |
| TCGA-6D-AA2E | 0 | 11 | 10 | 12 |
| TCGA-A3-3308 | 3 | NA | 19 | 32 |
| TCGA-A3-3322 | 6 | NA | 10 | 21 |
| TCGA-A3-3378 | 1 | 101 | 73 | 25 |
| TCGA-A3-3382 | 24 | NA | 34 | 31 |
| TCGA-AK-3436 | 20 | NA | 13 | 12 |
| TCGA-AK-3443 | 2 | NA | 1 | 8 |
| TCGA-AK-3455 | 2 | 15 | 132 | 32 |
| TCGA-AS-3778 | 1 | NA | 5 | 18 |
| TCGA-B0-4693 | 3 | 52 | 33 | NA |
| TCGA-B0-4821 | 9 | 2 | 23 | 34 |
| TCGA-B0-4823 | 6 | 14 | 42 | 29 |
| TCGA-B0-5077 | 7 | 1 | 35 | 19 |
| TCGA-B0-5102 | 15 | 2 | 22 | 24 |
| TCGA-B0-5109 | 21 | 115 | 49 | 16 |
| TCGA-B2-4099 | 1 | NA | 22 | 33 |
| TCGA-B4-5378 | 0 | NA | 2 | NA |
| TCGA-B4-5835 | 11 | 2 | 39 | 35 |
| TCGA-B4-5843 | 6 | NA | 7 | 16 |
| TCGA-B8-A54H | 1 | NA | 22 | 41 |
| TCGA-BP-4166 | 22 | 5 | 51 | NA |
| TCGA-BP-4170 | 3 | 3 | 96 | NA |
| TCGA-BP-4770 | 12 | NA | 19 | 30 |
| TCGA-BP-4774 | 1 | NA | 37 | NA |
| TCGA-BP-4776 | 1 | 28 | 6 | NA |
| TCGA-BP-4797 | 14 | NA | 106 | NA |
| TCGA-BP-5000 | 9 | NA | 63 | 15 |
| TCGA-BP-5184 | 2 | NA | 39 | 11 |
| TCGA-BP-5186 | 0 | NA | 15 | 18 |
| TCGA-CJ-4637 | 1 | 43 | 224 | 19 |
| TCGA-CJ-4869 | 1 | 3 | 262 | 40 |
| TCGA-CJ-4875 | 8 | NA | 28 | NA |
| TCGA-CJ-4908 | 1 | NA | 40 | 15 |
| TCGA-CJ-5684 | 3 | NA | 60 | 16 |
| TCGA-CW-5587 | 6 | 2 | 67 | 22 |
| TCGA-CW-5589 | NA | 3 | 22 | NA |
| TCGA-CW-5591 | 23 | 1 | 6 | 15 |
| TCGA-CZ-5985 | 5 | 25 | 56 | 18 |
| TCGA-CZ-5988 | 6 | NA | 20 | 19 |
| TCGA-DV-A4W0 | 5 | NA | 5 | 35 |
| TCGA-G6-A5PC | 1 | NA | 4 | 27 |
| TCGA-MM-A84U | 4 | 2 | 9 | 35 |
| TCGA-MW-A4EC | 4 | NA | 5 | 14 |
| TCGA-A3-3316 | 24 | NA | 17 | 18 |
| TCGA-A3-3320 | 1 | NA | 21 | 30 |
| TCGA-A3-3362 | NA | NA | 24 | 19 |
| TCGA-AK-3426 | 5 | NA | 124 | 11 |
| TCGA-AK-3447 | 7 | NA | 0 | NA |
| TCGA-AK-3453 | 26 | 1 | 0 | 6 |
| TCGA-AK-3460 | 4 | NA | 12 | NA |
| TCGA-B0-4700 | 0 | 37 | 96 | 7 |
| TCGA-B0-4811 | 25 | NA | 8 | 25 |
| TCGA-B0-4833 | 4 | 1 | 34 | NA |
| TCGA-B0-4836 | 13 | 19 | 73 | NA |
| TCGA-B0-4838 | 10 | 16 | 64 | NA |
| TCGA-B0-4839 | 7 | 5 | 14 | NA |
| TCGA-B0-4852 | 2 | 6 | 49 | 29 |
| TCGA-B0-5092 | 2 | 3 | 170 | 26 |
| TCGA-B0-5113 | 23 | 22 | 84 | 15 |
| TCGA-B0-5117 | 10 | NA | 2 | 4 |
| TCGA-B0-5120 | 2 | 4 | 22 | NA |
| TCGA-B0-5693 | 3 | 1 | 7 | 24 |
| TCGA-B0-5702 | NA | NA | 0 | 22 |
| TCGA-B2-4098 | 4 | 13 | 13 | 16 |
| TCGA-B8-4146 | 1 | NA | 21 | 17 |
| TCGA-B8-4151 | 1 | NA | 4 | 19 |
| TCGA-B8-A7U6 | 1 | NA | 9 | 20 |
| TCGA-BP-4162 | 10 | 1 | 43 | 19 |
| TCGA-BP-4165 | 1 | NA | 27 | NA |
| TCGA-BP-4340 | 5 | NA | 24 | NA |
| TCGA-BP-4346 | 1 | 6 | 151 | NA |
| TCGA-BP-4756 | 9 | 3 | 32 | NA |
| TCGA-BP-4760 | 0 | NA | 3 | 5 |
| TCGA-BP-4761 | 11 | 82 | 35 | NA |
| TCGA-BP-4766 | 4 | NA | 12 | NA |
| TCGA-BP-4795 | 1 | 2 | 82 | NA |
| TCGA-BP-4989 | 9 | 1 | 122 | 28 |
| TCGA-BP-5190 | 1 | 1 | 17 | 18 |
| TCGA-BP-5195 | 1 | 2 | 45 | 35 |
| TCGA-BP-5196 | 12 | 7 | 53 | 20 |
| TCGA-BP-5202 | 0 | NA | 15 | 20 |
| TCGA-CJ-4644 | 3 | NA | 57 | 27 |
| TCGA-CJ-4870 | NA | NA | 8 | NA |
| TCGA-CJ-4871 | 1 | NA | 33 | NA |
| TCGA-CJ-4885 | 11 | NA | 12 | NA |
| TCGA-CJ-4890 | NA | 76 | 154 | NA |
| TCGA-CJ-4907 | 0 | NA | 20 | 29 |
| TCGA-CJ-4918 | 0 | 19 | 38 | 29 |
| TCGA-CJ-5671 | 20 | 20 | 112 | 24 |
| TCGA-CJ-5681 | 0 | NA | 12 | 11 |
| TCGA-CZ-4854 | 29 | 29 | 36 | NA |
| TCGA-CZ-4860 | 19 | 2 | 45 | NA |
| TCGA-CZ-5451 | 4 | 1 | 16 | 43 |
| TCGA-CZ-5455 | 1 | NA | 133 | 18 |
| TCGA-CZ-5457 | 24 | 32 | 40 | 41 |
| TCGA-CZ-5459 | 2 | 6 | 37 | 37 |
| TCGA-CZ-5987 | 8 | 12 | 31 | 16 |
| TCGA-DV-5575 | 3 | 2 | 45 | NA |
| TCGA-A3-3306 | 7 | 1 | 21 | NA |
| TCGA-A3-3313 | 7 | NA | 7 | 31 |
| TCGA-A3-3343 | 1 | 5 | 52 | NA |
| TCGA-A3-3358 | 9 | 75 | 82 | 28 |
| TCGA-A3-3387 | 2 | 4 | 37 | 44 |
| TCGA-B0-4696 | 10 | NA | 1 | NA |
| TCGA-B0-4701 | 0 | 7 | 66 | NA |
| TCGA-B0-4818 | 1 | 124 | 65 | 23 |
| TCGA-B0-4827 | 3 | 152 | 70 | 40 |
| TCGA-B0-5084 | 8 | 1 | 11 | 17 |
| TCGA-B0-5104 | 1 | NA | 14 | 21 |
| TCGA-B0-5106 | 6 | 2 | 35 | 32 |
| TCGA-B0-5399 | NA | NA | 72 | 10 |
| TCGA-B0-5690 | 1 | NA | 31 | 31 |
| TCGA-B0-5692 | 2 | 6 | 147 | 17 |
| TCGA-B0-5698 | 3 | NA | 46 | 31 |
| TCGA-B0-5713 | 5 | NA | 26 | 44 |
| TCGA-B2-4101 | 0 | 11 | 63 | 18 |
| TCGA-B2-5633 | 6 | 2 | 40 | 24 |
| TCGA-B8-4143 | 10 | 43 | 80 | 33 |
| TCGA-B8-4620 | 18 | 1 | 26 | 30 |
| TCGA-B8-4621 | 12 | 1 | 19 | 19 |
| TCGA-B8-4622 | 8 | 3 | 34 | NA |
| TCGA-B8-5546 | 0 | NA | 5 | 4 |
| TCGA-B8-5553 | 0 | 2 | 42 | 10 |
| TCGA-B8-A54F | 3 | 1 | 4 | 11 |
| TCGA-BP-4167 | 5 | 3 | 84 | 9 |
| TCGA-BP-4173 | NA | 2 | 208 | NA |
| TCGA-BP-4174 | 14 | NA | 43 | NA |
| TCGA-BP-4177 | 6 | NA | 18 | NA |
| TCGA-BP-4326 | 1 | NA | 11 | NA |
| TCGA-BP-4330 | 0 | 102 | 113 | NA |
| TCGA-BP-4781 | NA | NA | 31 | NA |
| TCGA-BP-4803 | 4 | 7 | 47 | NA |
| TCGA-BP-4965 | 1 | NA | 36 | 21 |
| TCGA-BP-4967 | 11 | 21 | 29 | 33 |
| TCGA-BP-4993 | 1 | NA | NA | 22 |
| TCGA-CJ-4639 | 2 | NA | 54 | 30 |
| TCGA-CJ-4868 | 2 | 9 | 60 | NA |
| TCGA-CJ-4887 | 6 | 4 | 94 | NA |
| TCGA-CJ-4889 | NA | 432 | 109 | NA |
| TCGA-CJ-4913 | 1 | NA | NA | NA |
| TCGA-CJ-5680 | 2 | 1 | 16 | 11 |
| TCGA-CJ-5689 | 4 | 6 | 36 | 23 |
| TCGA-CJ-6028 | 31 | 68 | 94 | 27 |
| TCGA-CJ-6032 | 6 | NA | 37 | 17 |
| TCGA-CW-5581 | 2 | NA | 47 | 22 |
| TCGA-CW-6088 | 3 | NA | 15 | NA |
| TCGA-CW-6097 | 1 | 15 | 140 | 12 |
| TCGA-CZ-4861 | 0 | 12 | 72 | NA |
| TCGA-CZ-5452 | 4 | 53 | 259 | 12 |
| TCGA-CZ-5470 | 4 | 23 | 104 | 20 |
| TCGA-EU-5907 | 7 | NA | 4 | 20 |
| TCGA-A3-3326 | 0 | 5 | 37 | 12 |
| TCGA-A3-3349 | NA | NA | 18 | 22 |
| TCGA-A3-3372 | 22 | NA | 37 | NA |
| TCGA-A3-3373 | 4 | 1 | 18 | 25 |
| TCGA-AK-3444 | 4 | NA | 40 | 56 |
| TCGA-AK-3450 | 2 | 10 | 24 | NA |
| TCGA-B0-4694 | 5 | 57 | 158 | NA |
| TCGA-B0-4837 | 3 | NA | 21 | NA |
| TCGA-B0-4849 | 3 | NA | 18 | NA |
| TCGA-B0-5083 | NA | NA | 47 | 10 |
| TCGA-B0-5085 | 0 | NA | 11 | 31 |
| TCGA-B0-5095 | 5 | NA | 37 | 38 |
| TCGA-B0-5096 | 6 | 31 | 20 | 21 |
| TCGA-B0-5099 | 2 | NA | 7 | 26 |
| TCGA-B0-5107 | 5 | 1 | 63 | 20 |
| TCGA-B0-5116 | 11 | NA | NA | 26 |
| TCGA-B0-5701 | 5 | 3 | 40 | 54 |
| TCGA-B0-5707 | NA | NA | 8 | 18 |
| TCGA-B2-A4SR | 2 | NA | 19 | 17 |
| TCGA-B4-5832 | 24 | 4 | 9 | 28 |
| TCGA-B4-5844 | 1 | NA | 7 | 35 |
| TCGA-B8-5158 | 9 | 2 | 61 | 24 |
| TCGA-BP-4345 | NA | NA | 74 | NA |
| TCGA-BP-4972 | 2 | NA | 50 | 11 |
| TCGA-BP-4981 | 2 | NA | 31 | 27 |
| TCGA-BP-5168 | 3 | NA | 28 | 33 |
| TCGA-BP-5170 | 2 | NA | 27 | 16 |
| TCGA-BP-5183 | 5 | 1 | 52 | 13 |
| TCGA-BP-5194 | 1 | 6 | 31 | 14 |
| TCGA-CJ-4876 | 8 | 2 | 58 | NA |
| TCGA-CJ-4888 | NA | 157 | 130 | NA |
| TCGA-CJ-4894 | 3 | 42 | 99 | NA |
| TCGA-CJ-4903 | 1 | 3 | 45 | 25 |
| TCGA-CW-5584 | 1 | 5 | 18 | NA |
| TCGA-CW-5590 | NA | 1 | 36 | NA |
| TCGA-CZ-4857 | 11 | 1 | 45 | NA |
| TCGA-CZ-4864 | 4 | 6 | 59 | 24 |
| TCGA-CZ-5469 | 26 | NA | 27 | 26 |
| TCGA-CZ-5984 | 19 | 4 | 77 | 14 |
| TCGA-DV-A4VX | 16 | NA | 7 | 27 |
| TCGA-EU-5905 | 21 | NA | 7 | 16 |
