# Supplementary material for: Pyroptosis-Related lncRNA Prognostic Model for Renal Cancer Contributes to Immunodiagnosis and Immunotherapy
Source: Front Oncol. 2022 Jul 4;12:837155. doi: 10.3389/fonc.2022.837155 (PMC9291251; doi:10.3389/fonc.2022.837155)
Supplement: Supplementary file 9 [file Table_6.docx]

**Supplementary Table S6 Data of Disease-free survival in TCGA-cohort**

| Sample ID | Disease Free (Months) | Disease Free Status |
| --- | --- | --- |
| TCGA-3Z-A93Z-01 | 12.65 | 0:DiseaseFree |
| TCGA-6D-AA2E-01 | 11.89 | 0:DiseaseFree |
| TCGA-A3-3306-01 | 36.79 | 0:DiseaseFree |
| TCGA-A3-3307-01 | 47.17 | 0:DiseaseFree |
| TCGA-A3-3308-01 | 0.53 | 0:DiseaseFree |
| TCGA-A3-3311-01 | NA | NA |
| TCGA-A3-3313-01 | NA | NA |
| TCGA-A3-3316-01 | 49.05 | 0:DiseaseFree |
| TCGA-A3-3317-01 | 31.27 | 1:Recurred/Progressed |
| TCGA-A3-3319-01 | 37.12 | 0:DiseaseFree |
| TCGA-A3-3320-01 | 49.54 | 0:DiseaseFree |
| TCGA-A3-3322-01 | 48.55 | 0:DiseaseFree |
| TCGA-A3-3323-01 | 36.33 | 0:DiseaseFree |
| TCGA-A3-3324-01 | 38.96 | 0:DiseaseFree |
| TCGA-A3-3325-01 | NA | NA |
| TCGA-A3-3326-01 | 37.35 | 0:DiseaseFree |
| TCGA-A3-3328-01 | 45.5 | 0:DiseaseFree |
| TCGA-A3-3329-01 | 53.35 | 0:DiseaseFree |
| TCGA-A3-3331-01 | 48.78 | 0:DiseaseFree |
| TCGA-A3-3335-01 | 36.07 | 1:Recurred/Progressed |
| TCGA-A3-3336-01 | 34.26 | 0:DiseaseFree |
| TCGA-A3-3343-01 | 31.04 | 0:DiseaseFree |
| TCGA-A3-3346-01 | 3.75 | 1:Recurred/Progressed |
| TCGA-A3-3347-01 | 20.04 | 1:Recurred/Progressed |
| TCGA-A3-3349-01 | 45.5 | 0:DiseaseFree |
| TCGA-A3-3351-01 | 29.89 | 0:DiseaseFree |
| TCGA-A3-3352-01 | NA | NA |
| TCGA-A3-3357-01 | 88.3 | 0:DiseaseFree |
| TCGA-A3-3358-01 | 42.94 | 0:DiseaseFree |
| TCGA-A3-3359-01 | 82.26 | 0:DiseaseFree |
| TCGA-A3-3362-01 | 51.22 | 0:DiseaseFree |
| TCGA-A3-3363-01 | 10.48 | 0:DiseaseFree |
| TCGA-A3-3365-01 | 28.68 | 0:DiseaseFree |
| TCGA-A3-3367-01 | 74.57 | 0:DiseaseFree |
| TCGA-A3-3370-01 | 74.7 | 0:DiseaseFree |
| TCGA-A3-3372-01 | 24.15 | 0:DiseaseFree |
| TCGA-A3-3373-01 | 53.25 | 0:DiseaseFree |
| TCGA-A3-3374-01 | 43.17 | 0:DiseaseFree |
| TCGA-A3-3376-01 | NA | NA |
| TCGA-A3-3378-01 | 20.7 | 0:DiseaseFree |
| TCGA-A3-3380-01 | 18.63 | 0:DiseaseFree |
| TCGA-A3-3382-01 | 16.89 | 1:Recurred/Progressed |
| TCGA-A3-3383-01 | 28.29 | 0:DiseaseFree |
| TCGA-A3-3385-01 | 65.47 | 0:DiseaseFree |
| TCGA-A3-3387-01 | 20.27 | 0:DiseaseFree |
| TCGA-A3-A6NI-01 | 33.44 | 0:DiseaseFree |
| TCGA-A3-A6NJ-01 | 15.37 | 0:DiseaseFree |
| TCGA-A3-A6NL-01 | 22.63 | 0:DiseaseFree |
| TCGA-A3-A6NN-01 | 0.1 | 0:DiseaseFree |
| TCGA-A3-A8CQ-01 | 0.1 | 0:DiseaseFree |
| TCGA-A3-A8OU-01 | 0 | 0:DiseaseFree |
| TCGA-A3-A8OV-01 | 11.17 | 0:DiseaseFree |
| TCGA-A3-A8OW-01 | 10.61 | 0:DiseaseFree |
| TCGA-A3-A8OX-01 | 0 | 0:DiseaseFree |
| TCGA-AK-3425-01 | 109.82 | 0:DiseaseFree |
| TCGA-AK-3426-01 | 8.41 | 1:Recurred/Progressed |
| TCGA-AK-3427-01 | 117.71 | 0:DiseaseFree |
| TCGA-AK-3428-01 | 122.47 | 0:DiseaseFree |
| TCGA-AK-3429-01 | 109.33 | 0:DiseaseFree |
| TCGA-AK-3430-01 | NA | NA |
| TCGA-AK-3431-01 | NA | NA |
| TCGA-AK-3433-01 | 111.99 | 0:DiseaseFree |
| TCGA-AK-3434-01 | 53.88 | 1:Recurred/Progressed |
| TCGA-AK-3436-01 | 39.06 | 1:Recurred/Progressed |
| TCGA-AK-3440-01 | 94.12 | 0:DiseaseFree |
| TCGA-AK-3443-01 | 46.75 | 0:DiseaseFree |
| TCGA-AK-3444-01 | 48.32 | 0:DiseaseFree |
| TCGA-AK-3445-01 | 78.58 | 0:DiseaseFree |
| TCGA-AK-3447-01 | 39.98 | 0:DiseaseFree |
| TCGA-AK-3450-01 | 58.44 | 0:DiseaseFree |
| TCGA-AK-3451-01 | 94.22 | 0:DiseaseFree |
| TCGA-AK-3453-01 | 83.15 | 0:DiseaseFree |
| TCGA-AK-3454-01 | 28.71 | 0:DiseaseFree |
| TCGA-AK-3455-01 | NA | NA |
| TCGA-AK-3456-01 | 37.55 | 0:DiseaseFree |
| TCGA-AK-3458-01 | 38.37 | 0:DiseaseFree |
| TCGA-AK-3460-01 | 82.39 | 0:DiseaseFree |
| TCGA-AK-3461-01 | 72.83 | 0:DiseaseFree |
| TCGA-AK-3465-01 | 12.12 | 0:DiseaseFree |
| TCGA-AS-3777-01 | 40.67 | 0:DiseaseFree |
| TCGA-AS-3778-01 | 1.41 | 0:DiseaseFree |
| TCGA-B0-4688-01 | NA | NA |
| TCGA-B0-4690-01 | NA | NA |
| TCGA-B0-4691-01 | NA | NA |
| TCGA-B0-4693-01 | NA | NA |
| TCGA-B0-4694-01 | NA | NA |
| TCGA-B0-4696-01 | 26.08 | 1:Recurred/Progressed |
| TCGA-B0-4697-01 | NA | NA |
| TCGA-B0-4698-01 | NA | NA |
| TCGA-B0-4699-01 | NA | NA |
| TCGA-B0-4700-01 | NA | NA |
| TCGA-B0-4701-01 | NA | NA |
| TCGA-B0-4703-01 | NA | NA |
| TCGA-B0-4706-01 | NA | NA |
| TCGA-B0-4707-01 | NA | NA |
| TCGA-B0-4710-01 | 57.65 | 0:DiseaseFree |
| TCGA-B0-4712-01 | NA | NA |
| TCGA-B0-4713-01 | NA | NA |
| TCGA-B0-4714-01 | NA | NA |
| TCGA-B0-4718-01 | 25.23 | 1:Recurred/Progressed |
| TCGA-B0-4810-01 | 6.73 | 1:Recurred/Progressed |
| TCGA-B0-4811-01 | 15.05 | 1:Recurred/Progressed |
| TCGA-B0-4813-01 | NA | NA |
| TCGA-B0-4814-01 | NA | NA |
| TCGA-B0-4815-01 | NA | NA |
| TCGA-B0-4816-01 | NA | NA |
| TCGA-B0-4817-01 | NA | NA |
| TCGA-B0-4818-01 | 4.17 | 1:Recurred/Progressed |
| TCGA-B0-4819-01 | NA | NA |
| TCGA-B0-4821-01 | NA | NA |
| TCGA-B0-4822-01 | NA | NA |
| TCGA-B0-4823-01 | NA | NA |
| TCGA-B0-4824-01 | NA | NA |
| TCGA-B0-4827-01 | 21.94 | 1:Recurred/Progressed |
| TCGA-B0-4828-01 | NA | NA |
| TCGA-B0-4833-01 | NA | NA |
| TCGA-B0-4834-01 | NA | NA |
| TCGA-B0-4836-01 | NA | NA |
| TCGA-B0-4837-01 | 39.68 | 1:Recurred/Progressed |
| TCGA-B0-4838-01 | NA | NA |
| TCGA-B0-4839-01 | NA | NA |
| TCGA-B0-4841-01 | 3.55 | 1:Recurred/Progressed |
| TCGA-B0-4842-01 | 8.21 | 1:Recurred/Progressed |
| TCGA-B0-4843-01 | NA | NA |
| TCGA-B0-4844-01 | 2.04 | 1:Recurred/Progressed |
| TCGA-B0-4845-01 | 24.57 | 1:Recurred/Progressed |
| TCGA-B0-4846-01 | 5.35 | 1:Recurred/Progressed |
| TCGA-B0-4847-01 | NA | NA |
| TCGA-B0-4848-01 | 16.23 | 1:Recurred/Progressed |
| TCGA-B0-4849-01 | 0.13 | 1:Recurred/Progressed |
| TCGA-B0-4852-01 | 28.25 | 1:Recurred/Progressed |
| TCGA-B0-4945-01 | NA | NA |
| TCGA-B0-5075-01 | NA | NA |
| TCGA-B0-5077-01 | NA | NA |
| TCGA-B0-5080-01 | 3.38 | 1:Recurred/Progressed |
| TCGA-B0-5081-01 | 7 | 1:Recurred/Progressed |
| TCGA-B0-5083-01 | NA | NA |
| TCGA-B0-5084-01 | 2.14 | 1:Recurred/Progressed |
| TCGA-B0-5085-01 | NA | NA |
| TCGA-B0-5088-01 | NA | NA |
| TCGA-B0-5092-01 | NA | NA |
| TCGA-B0-5094-01 | 2.46 | 1:Recurred/Progressed |
| TCGA-B0-5095-01 | NA | NA |
| TCGA-B0-5096-01 | NA | NA |
| TCGA-B0-5097-01 | 11.79 | 1:Recurred/Progressed |
| TCGA-B0-5098-01 | NA | NA |
| TCGA-B0-5099-01 | NA | NA |
| TCGA-B0-5100-01 | 46.85 | 1:Recurred/Progressed |
| TCGA-B0-5102-01 | 89.82 | 1:Recurred/Progressed |
| TCGA-B0-5104-01 | NA | NA |
| TCGA-B0-5106-01 | NA | NA |
| TCGA-B0-5107-01 | 18.04 | 1:Recurred/Progressed |
| TCGA-B0-5108-01 | 58.54 | 0:DiseaseFree |
| TCGA-B0-5109-01 | 2.3 | 1:Recurred/Progressed |
| TCGA-B0-5110-01 | 66 | 0:DiseaseFree |
| TCGA-B0-5113-01 | 38.6 | 0:DiseaseFree |
| TCGA-B0-5115-01 | 2.46 | 1:Recurred/Progressed |
| TCGA-B0-5116-01 | 37.09 | 1:Recurred/Progressed |
| TCGA-B0-5117-01 | 52.83 | 0:DiseaseFree |
| TCGA-B0-5119-01 | 50.99 | 0:DiseaseFree |
| TCGA-B0-5120-01 | 38.4 | 0:DiseaseFree |
| TCGA-B0-5121-01 | 48.78 | 0:DiseaseFree |
| TCGA-B0-5399-01 | 46.35 | 0:DiseaseFree |
| TCGA-B0-5400-01 | 56.93 | 0:DiseaseFree |
| TCGA-B0-5402-01 | 14.75 | 1:Recurred/Progressed |
| TCGA-B0-5690-01 | 111.43 | 0:DiseaseFree |
| TCGA-B0-5691-01 | 106.77 | 1:Recurred/Progressed |
| TCGA-B0-5692-01 | 129.57 | 0:DiseaseFree |
| TCGA-B0-5693-01 | 133.84 | 0:DiseaseFree |
| TCGA-B0-5694-01 | 9.46 | 1:Recurred/Progressed |
| TCGA-B0-5695-01 | 70.63 | 0:DiseaseFree |
| TCGA-B0-5696-01 | 56.73 | 1:Recurred/Progressed |
| TCGA-B0-5697-01 | 86.4 | 0:DiseaseFree |
| TCGA-B0-5698-01 | 119.28 | 0:DiseaseFree |
| TCGA-B0-5699-01 | 3.09 | 1:Recurred/Progressed |
| TCGA-B0-5700-01 | 58.8 | 0:DiseaseFree |
| TCGA-B0-5701-01 | 7.36 | 1:Recurred/Progressed |
| TCGA-B0-5702-01 | 71.35 | 0:DiseaseFree |
| TCGA-B0-5703-01 | 73.78 | 0:DiseaseFree |
| TCGA-B0-5705-01 | 27.66 | 1:Recurred/Progressed |
| TCGA-B0-5706-01 | 105.29 | 0:DiseaseFree |
| TCGA-B0-5707-01 | 123 | 0:DiseaseFree |
| TCGA-B0-5709-01 | 130.55 | 0:DiseaseFree |
| TCGA-B0-5710-01 | 47.54 | 1:Recurred/Progressed |
| TCGA-B0-5711-01 | 123.72 | 1:Recurred/Progressed |
| TCGA-B0-5712-01 | 23.82 | 1:Recurred/Progressed |
| TCGA-B0-5713-01 | 91.39 | 0:DiseaseFree |
| TCGA-B0-5812-01 | 125.95 | 0:DiseaseFree |
| TCGA-B2-3923-01 | 32.59 | 0:DiseaseFree |
| TCGA-B2-3924-01 | 35.87 | 0:DiseaseFree |
| TCGA-B2-4098-01 | NA | NA |
| TCGA-B2-4099-01 | 31.93 | 0:DiseaseFree |
| TCGA-B2-4101-01 | 21.29 | 0:DiseaseFree |
| TCGA-B2-4102-01 | 31.27 | 0:DiseaseFree |
| TCGA-B2-5633-01 | 31.64 | 0:DiseaseFree |
| TCGA-B2-5635-01 | 24.8 | 0:DiseaseFree |
| TCGA-B2-5636-01 | 30.19 | 0:DiseaseFree |
| TCGA-B2-5639-01 | 27.96 | 1:Recurred/Progressed |
| TCGA-B2-5641-01 | 21.55 | 0:DiseaseFree |
| TCGA-B2-A4SR-01 | 8.74 | 1:Recurred/Progressed |
| TCGA-B4-5377-01 | 11.99 | 0:DiseaseFree |
| TCGA-B4-5378-01 | 5.75 | 0:DiseaseFree |
| TCGA-B4-5832-01 | 5.09 | 0:DiseaseFree |
| TCGA-B4-5834-01 | 1.25 | 0:DiseaseFree |
| TCGA-B4-5835-01 | 0.53 | 0:DiseaseFree |
| TCGA-B4-5836-01 | 4.63 | 0:DiseaseFree |
| TCGA-B4-5838-01 | 5.45 | 0:DiseaseFree |
| TCGA-B4-5843-01 | 0.36 | 0:DiseaseFree |
| TCGA-B4-5844-01 | 0.23 | 0:DiseaseFree |
| TCGA-B8-4143-01 | NA | NA |
| TCGA-B8-4146-01 | 16.79 | 0:DiseaseFree |
| TCGA-B8-4148-01 | 49.93 | 0:DiseaseFree |
| TCGA-B8-4151-01 | 42.67 | 0:DiseaseFree |
| TCGA-B8-4153-01 | 7.33 | 1:Recurred/Progressed |
| TCGA-B8-4154-01 | 45.34 | 0:DiseaseFree |
| TCGA-B8-4619-01 | 17.18 | 0:DiseaseFree |
| TCGA-B8-4620-01 | 19.84 | 1:Recurred/Progressed |
| TCGA-B8-4621-01 | 25.89 | 0:DiseaseFree |
| TCGA-B8-4622-01 | 40.31 | 1:Recurred/Progressed |
| TCGA-B8-5158-01 | 40.01 | 0:DiseaseFree |
| TCGA-B8-5159-01 | 23.72 | 0:DiseaseFree |
| TCGA-B8-5162-01 | 1.18 | 0:DiseaseFree |
| TCGA-B8-5163-01 | 27 | 0:DiseaseFree |
| TCGA-B8-5164-01 | 0.85 | 0:DiseaseFree |
| TCGA-B8-5165-01 | 24.21 | 0:DiseaseFree |
| TCGA-B8-5545-01 | 50.1 | 0:DiseaseFree |
| TCGA-B8-5546-01 | 16.59 | 0:DiseaseFree |
| TCGA-B8-5549-01 | 6.37 | 0:DiseaseFree |
| TCGA-B8-5550-01 | 22.8 | 1:Recurred/Progressed |
| TCGA-B8-5551-01 | 0.53 | 0:DiseaseFree |
| TCGA-B8-5552-01 | 34.36 | 0:DiseaseFree |
| TCGA-B8-5553-01 | 14.29 | 0:DiseaseFree |
| TCGA-B8-A54D-01 | 27.27 | 0:DiseaseFree |
| TCGA-B8-A54E-01 | 29.86 | 0:DiseaseFree |
| TCGA-B8-A54F-01 | 17.05 | 0:DiseaseFree |
| TCGA-B8-A54G-01 | 1.74 | 0:DiseaseFree |
| TCGA-B8-A54H-01 | 8.41 | 0:DiseaseFree |
| TCGA-B8-A54I-01 | 4.93 | 0:DiseaseFree |
| TCGA-B8-A54J-01 | 17.35 | 0:DiseaseFree |
| TCGA-B8-A54K-01 | 15.41 | 0:DiseaseFree |
| TCGA-B8-A7U6-01 | 16.26 | 0:DiseaseFree |
| TCGA-B8-A8YJ-01 | 14.16 | 0:DiseaseFree |
| TCGA-BP-4158-01 | 110.94 | 0:DiseaseFree |
| TCGA-BP-4159-01 | 77.27 | 1:Recurred/Progressed |
| TCGA-BP-4160-01 | 94.65 | 0:DiseaseFree |
| TCGA-BP-4161-01 | 88.21 | 1:Recurred/Progressed |
| TCGA-BP-4162-01 | 100.99 | 0:DiseaseFree |
| TCGA-BP-4163-01 | 93.27 | 0:DiseaseFree |
| TCGA-BP-4164-01 | NA | NA |
| TCGA-BP-4165-01 | 60.38 | 1:Recurred/Progressed |
| TCGA-BP-4166-01 | 0.43 | 0:DiseaseFree |
| TCGA-BP-4167-01 | 89.29 | 0:DiseaseFree |
| TCGA-BP-4169-01 | 2.56 | 1:Recurred/Progressed |
| TCGA-BP-4170-01 | NA | NA |
| TCGA-BP-4173-01 | 62.19 | 0:DiseaseFree |
| TCGA-BP-4174-01 | 61.73 | 0:DiseaseFree |
| TCGA-BP-4176-01 | 64.22 | 0:DiseaseFree |
| TCGA-BP-4177-01 | 54.86 | 0:DiseaseFree |
| TCGA-BP-4325-01 | 97.37 | 0:DiseaseFree |
| TCGA-BP-4326-01 | 31.47 | 1:Recurred/Progressed |
| TCGA-BP-4327-01 | NA | NA |
| TCGA-BP-4329-01 | 23 | 1:Recurred/Progressed |
| TCGA-BP-4330-01 | 62.02 | 0:DiseaseFree |
| TCGA-BP-4331-01 | NA | NA |
| TCGA-BP-4332-01 | 37.22 | 0:DiseaseFree |
| TCGA-BP-4334-01 | 14.52 | 1:Recurred/Progressed |
| TCGA-BP-4335-01 | 14.91 | 1:Recurred/Progressed |
| TCGA-BP-4337-01 | NA | NA |
| TCGA-BP-4338-01 | 84.49 | 1:Recurred/Progressed |
| TCGA-BP-4340-01 | NA | NA |
| TCGA-BP-4341-01 | NA | NA |
| TCGA-BP-4342-01 | 7.88 | 1:Recurred/Progressed |
| TCGA-BP-4343-01 | 61.63 | 1:Recurred/Progressed |
| TCGA-BP-4344-01 | 54.73 | 0:DiseaseFree |
| TCGA-BP-4345-01 | 49.8 | 0:DiseaseFree |
| TCGA-BP-4346-01 | NA | NA |
| TCGA-BP-4347-01 | 44.91 | 0:DiseaseFree |
| TCGA-BP-4349-01 | 12.22 | 0:DiseaseFree |
| TCGA-BP-4351-01 | 28.68 | 1:Recurred/Progressed |
| TCGA-BP-4352-01 | 8.71 | 1:Recurred/Progressed |
| TCGA-BP-4353-01 | NA | NA |
| TCGA-BP-4354-01 | 22.5 | 1:Recurred/Progressed |
| TCGA-BP-4355-01 | NA | NA |
| TCGA-BP-4756-01 | 12.29 | 0:DiseaseFree |
| TCGA-BP-4758-01 | 72.54 | 0:DiseaseFree |
| TCGA-BP-4759-01 | 77.92 | 0:DiseaseFree |
| TCGA-BP-4760-01 | 47.86 | 1:Recurred/Progressed |
| TCGA-BP-4761-01 | 5.98 | 0:DiseaseFree |
| TCGA-BP-4762-01 | NA | NA |
| TCGA-BP-4763-01 | NA | NA |
| TCGA-BP-4765-01 | 71.75 | 0:DiseaseFree |
| TCGA-BP-4766-01 | 48.03 | 0:DiseaseFree |
| TCGA-BP-4768-01 | 13.14 | 0:DiseaseFree |
| TCGA-BP-4769-01 | 61.63 | 0:DiseaseFree |
| TCGA-BP-4770-01 | 6.83 | 1:Recurred/Progressed |
| TCGA-BP-4771-01 | 3.61 | 1:Recurred/Progressed |
| TCGA-BP-4774-01 | 61.93 | 0:DiseaseFree |
| TCGA-BP-4775-01 | 60.55 | 0:DiseaseFree |
| TCGA-BP-4776-01 | 13.5 | 0:DiseaseFree |
| TCGA-BP-4777-01 | 56.87 | 0:DiseaseFree |
| TCGA-BP-4781-01 | 68.33 | 0:DiseaseFree |
| TCGA-BP-4782-01 | 11.63 | 0:DiseaseFree |
| TCGA-BP-4784-01 | 60.91 | 0:DiseaseFree |
| TCGA-BP-4787-01 | 14.49 | 1:Recurred/Progressed |
| TCGA-BP-4789-01 | 48.92 | 0:DiseaseFree |
| TCGA-BP-4790-01 | NA | NA |
| TCGA-BP-4795-01 | 20.37 | 0:DiseaseFree |
| TCGA-BP-4797-01 | 36.37 | 0:DiseaseFree |
| TCGA-BP-4798-01 | NA | NA |
| TCGA-BP-4799-01 | 11.56 | 1:Recurred/Progressed |
| TCGA-BP-4801-01 | 36.93 | 0:DiseaseFree |
| TCGA-BP-4803-01 | 6.7 | 0:DiseaseFree |
| TCGA-BP-4804-01 | 41.72 | 1:Recurred/Progressed |
| TCGA-BP-4807-01 | 6.93 | 0:DiseaseFree |
| TCGA-BP-4959-01 | 87.39 | 0:DiseaseFree |
| TCGA-BP-4960-01 | 71.35 | 0:DiseaseFree |
| TCGA-BP-4961-01 | 63.57 | 0:DiseaseFree |
| TCGA-BP-4962-01 | 58.64 | 0:DiseaseFree |
| TCGA-BP-4963-01 | 60.25 | 0:DiseaseFree |
| TCGA-BP-4964-01 | 61.17 | 0:DiseaseFree |
| TCGA-BP-4965-01 | 61.47 | 0:DiseaseFree |
| TCGA-BP-4967-01 | 6.73 | 0:DiseaseFree |
| TCGA-BP-4968-01 | 57.36 | 0:DiseaseFree |
| TCGA-BP-4969-01 | 58.94 | 0:DiseaseFree |
| TCGA-BP-4970-01 | 14.22 | 0:DiseaseFree |
| TCGA-BP-4971-01 | 48.85 | 0:DiseaseFree |
| TCGA-BP-4972-01 | 49.34 | 0:DiseaseFree |
| TCGA-BP-4973-01 | 45.47 | 0:DiseaseFree |
| TCGA-BP-4974-01 | 1.15 | 1:Recurred/Progressed |
| TCGA-BP-4975-01 | 47.08 | 0:DiseaseFree |
| TCGA-BP-4976-01 | 53.61 | 0:DiseaseFree |
| TCGA-BP-4977-01 | 14.91 | 0:DiseaseFree |
| TCGA-BP-4981-01 | NA | NA |
| TCGA-BP-4982-01 | 33.31 | 0:DiseaseFree |
| TCGA-BP-4983-01 | 46.42 | 0:DiseaseFree |
| TCGA-BP-4985-01 | 21.58 | 1:Recurred/Progressed |
| TCGA-BP-4986-01 | 25.79 | 0:DiseaseFree |
| TCGA-BP-4987-01 | 36.93 | 0:DiseaseFree |
| TCGA-BP-4988-01 | NA | NA |
| TCGA-BP-4989-01 | 3.88 | 0:DiseaseFree |
| TCGA-BP-4991-01 | 46.42 | 0:DiseaseFree |
| TCGA-BP-4992-01 | 16.46 | 0:DiseaseFree |
| TCGA-BP-4993-01 | 5.81 | 0:DiseaseFree |
| TCGA-BP-4994-01 | 42.97 | 0:DiseaseFree |
| TCGA-BP-4995-01 | 45.04 | 0:DiseaseFree |
| TCGA-BP-4998-01 | 30.62 | 0:DiseaseFree |
| TCGA-BP-4999-01 | 41.59 | 0:DiseaseFree |
| TCGA-BP-5000-01 | 18.5 | 0:DiseaseFree |
| TCGA-BP-5001-01 | 38.67 | 0:DiseaseFree |
| TCGA-BP-5004-01 | 36.99 | 0:DiseaseFree |
| TCGA-BP-5006-01 | 27.6 | 0:DiseaseFree |
| TCGA-BP-5007-01 | 37.45 | 0:DiseaseFree |
| TCGA-BP-5008-01 | 35.18 | 0:DiseaseFree |
| TCGA-BP-5009-01 | 16.16 | 1:Recurred/Progressed |
| TCGA-BP-5010-01 | 25.66 | 1:Recurred/Progressed |
| TCGA-BP-5168-01 | NA | NA |
| TCGA-BP-5169-01 | 6.34 | 0:DiseaseFree |
| TCGA-BP-5170-01 | 79.24 | 0:DiseaseFree |
| TCGA-BP-5173-01 | NA | NA |
| TCGA-BP-5174-01 | 74.15 | 0:DiseaseFree |
| TCGA-BP-5175-01 | 30.62 | 0:DiseaseFree |
| TCGA-BP-5176-01 | NA | NA |
| TCGA-BP-5177-01 | 9.63 | 0:DiseaseFree |
| TCGA-BP-5178-01 | 56.14 | 1:Recurred/Progressed |
| TCGA-BP-5180-01 | 74.34 | 0:DiseaseFree |
| TCGA-BP-5181-01 | 49.11 | 0:DiseaseFree |
| TCGA-BP-5182-01 | 38.27 | 0:DiseaseFree |
| TCGA-BP-5183-01 | 15.93 | 1:Recurred/Progressed |
| TCGA-BP-5184-01 | 37.22 | 0:DiseaseFree |
| TCGA-BP-5185-01 | 24.18 | 1:Recurred/Progressed |
| TCGA-BP-5186-01 | 22.77 | 0:DiseaseFree |
| TCGA-BP-5187-01 | 13.34 | 0:DiseaseFree |
| TCGA-BP-5189-01 | 25.66 | 1:Recurred/Progressed |
| TCGA-BP-5190-01 | 33.21 | 0:DiseaseFree |
| TCGA-BP-5191-01 | 31.77 | 0:DiseaseFree |
| TCGA-BP-5192-01 | 23.46 | 0:DiseaseFree |
| TCGA-BP-5194-01 | 13.4 | 0:DiseaseFree |
| TCGA-BP-5195-01 | 24.61 | 0:DiseaseFree |
| TCGA-BP-5196-01 | 33.44 | 0:DiseaseFree |
| TCGA-BP-5198-01 | 19.81 | 0:DiseaseFree |
| TCGA-BP-5199-01 | 44.51 | 0:DiseaseFree |
| TCGA-BP-5200-01 | 34.92 | 0:DiseaseFree |
| TCGA-BP-5201-01 | -0.62 | 1:Recurred/Progressed |
| TCGA-BP-5202-01 | 0.95 | 0:DiseaseFree |
| TCGA-CJ-4634-01 | 114.91 | 0:DiseaseFree |
| TCGA-CJ-4635-01 | 46.52 | 0:DiseaseFree |
| TCGA-CJ-4636-01 | 63.21 | 0:DiseaseFree |
| TCGA-CJ-4637-01 | 59.82 | 1:Recurred/Progressed |
| TCGA-CJ-4638-01 | 2.3 | 1:Recurred/Progressed |
| TCGA-CJ-4639-01 | 106.08 | 0:DiseaseFree |
| TCGA-CJ-4640-01 | 114.32 | 0:DiseaseFree |
| TCGA-CJ-4641-01 | -11.79 | 1:Recurred/Progressed |
| TCGA-CJ-4642-01 | 105.29 | 0:DiseaseFree |
| TCGA-CJ-4643-01 | 58.9 | 0:DiseaseFree |
| TCGA-CJ-4644-01 | 3.06 | 1:Recurred/Progressed |
| TCGA-CJ-4868-01 | 1.22 | 1:Recurred/Progressed |
| TCGA-CJ-4869-01 | 57.62 | 1:Recurred/Progressed |
| TCGA-CJ-4870-01 | 49.21 | 0:DiseaseFree |
| TCGA-CJ-4871-01 | 5.55 | 1:Recurred/Progressed |
| TCGA-CJ-4872-01 | 47.14 | 0:DiseaseFree |
| TCGA-CJ-4873-01 | 74.21 | 0:DiseaseFree |
| TCGA-CJ-4874-01 | 75 | 0:DiseaseFree |
| TCGA-CJ-4875-01 | 17.44 | 1:Recurred/Progressed |
| TCGA-CJ-4876-01 | 64.22 | 0:DiseaseFree |
| TCGA-CJ-4878-01 | 71.81 | 0:DiseaseFree |
| TCGA-CJ-4881-01 | 61.79 | 1:Recurred/Progressed |
| TCGA-CJ-4882-01 | 61.86 | 0:DiseaseFree |
| TCGA-CJ-4884-01 | 57.79 | 0:DiseaseFree |
| TCGA-CJ-4885-01 | 67.05 | 1:Recurred/Progressed |
| TCGA-CJ-4886-01 | 64.13 | 0:DiseaseFree |
| TCGA-CJ-4887-01 | 10.58 | 1:Recurred/Progressed |
| TCGA-CJ-4888-01 | 19.97 | 1:Recurred/Progressed |
| TCGA-CJ-4889-01 | 63.93 | 0:DiseaseFree |
| TCGA-CJ-4890-01 | 48.52 | 1:Recurred/Progressed |
| TCGA-CJ-4891-01 | NA | NA |
| TCGA-CJ-4892-01 | 49.97 | 0:DiseaseFree |
| TCGA-CJ-4893-01 | 24.64 | 0:DiseaseFree |
| TCGA-CJ-4894-01 | 5.19 | 1:Recurred/Progressed |
| TCGA-CJ-4895-01 | 2 | 1:Recurred/Progressed |
| TCGA-CJ-4897-01 | 46.75 | 1:Recurred/Progressed |
| TCGA-CJ-4899-01 | 50.2 | 0:DiseaseFree |
| TCGA-CJ-4900-01 | NA | NA |
| TCGA-CJ-4901-01 | 47.63 | 0:DiseaseFree |
| TCGA-CJ-4902-01 | 49.93 | 0:DiseaseFree |
| TCGA-CJ-4903-01 | 51.25 | 0:DiseaseFree |
| TCGA-CJ-4904-01 | 9.13 | 1:Recurred/Progressed |
| TCGA-CJ-4905-01 | 49.15 | 0:DiseaseFree |
| TCGA-CJ-4907-01 | 49.24 | 0:DiseaseFree |
| TCGA-CJ-4908-01 | 50.3 | 0:DiseaseFree |
| TCGA-CJ-4912-01 | 54.43 | 0:DiseaseFree |
| TCGA-CJ-4913-01 | 17.67 | 1:Recurred/Progressed |
| TCGA-CJ-4916-01 | 45.11 | 0:DiseaseFree |
| TCGA-CJ-4918-01 | 1.08 | 1:Recurred/Progressed |
| TCGA-CJ-4920-01 | NA | NA |
| TCGA-CJ-4923-01 | 0.82 | 1:Recurred/Progressed |
| TCGA-CJ-5671-01 | 130.98 | 0:DiseaseFree |
| TCGA-CJ-5672-01 | NA | NA |
| TCGA-CJ-5675-01 | 29.57 | 1:Recurred/Progressed |
| TCGA-CJ-5676-01 | 72.9 | 1:Recurred/Progressed |
| TCGA-CJ-5677-01 | 9.46 | 1:Recurred/Progressed |
| TCGA-CJ-5678-01 | 13.11 | 1:Recurred/Progressed |
| TCGA-CJ-5679-01 | 19.55 | 1:Recurred/Progressed |
| TCGA-CJ-5680-01 | 5.03 | 1:Recurred/Progressed |
| TCGA-CJ-5681-01 | 0.56 | 1:Recurred/Progressed |
| TCGA-CJ-5682-01 | 1.81 | 1:Recurred/Progressed |
| TCGA-CJ-5683-01 | 62.06 | 0:DiseaseFree |
| TCGA-CJ-5684-01 | 73.29 | 0:DiseaseFree |
| TCGA-CJ-5686-01 | 66.95 | 0:DiseaseFree |
| TCGA-CJ-5689-01 | NA | NA |
| TCGA-CJ-6027-01 | NA | NA |
| TCGA-CJ-6028-01 | 4.5 | 1:Recurred/Progressed |
| TCGA-CJ-6030-01 | NA | NA |
| TCGA-CJ-6031-01 | 62.61 | 0:DiseaseFree |
| TCGA-CJ-6032-01 | 119.55 | 0:DiseaseFree |
| TCGA-CJ-6033-01 | 4.73 | 1:Recurred/Progressed |
| TCGA-CW-5580-01 | NA | NA |
| TCGA-CW-5581-01 | 91.95 | 0:DiseaseFree |
| TCGA-CW-5583-01 | 81.77 | 0:DiseaseFree |
| TCGA-CW-5584-01 | 3.35 | 1:Recurred/Progressed |
| TCGA-CW-5585-01 | 85.71 | 0:DiseaseFree |
| TCGA-CW-5587-01 | 65.8 | 1:Recurred/Progressed |
| TCGA-CW-5588-01 | 66.26 | 0:DiseaseFree |
| TCGA-CW-5589-01 | 78.12 | 0:DiseaseFree |
| TCGA-CW-5590-01 | 22.08 | 1:Recurred/Progressed |
| TCGA-CW-5591-01 | 74.61 | 0:DiseaseFree |
| TCGA-CW-6087-01 | NA | NA |
| TCGA-CW-6088-01 | 105.85 | 0:DiseaseFree |
| TCGA-CW-6090-01 | 91.33 | 1:Recurred/Progressed |
| TCGA-CW-6093-01 | 103.35 | 0:DiseaseFree |
| TCGA-CW-6096-01 | 88.73 | 0:DiseaseFree |
| TCGA-CW-6097-01 | 15.01 | 1:Recurred/Progressed |
| TCGA-CZ-4853-01 | 25.43 | 0:DiseaseFree |
| TCGA-CZ-4854-01 | NA | NA |
| TCGA-CZ-4856-01 | 0.59 | 0:DiseaseFree |
| TCGA-CZ-4857-01 | 32.52 | 1:Recurred/Progressed |
| TCGA-CZ-4858-01 | 62.81 | 1:Recurred/Progressed |
| TCGA-CZ-4859-01 | 58.71 | 0:DiseaseFree |
| TCGA-CZ-4860-01 | NA | NA |
| TCGA-CZ-4861-01 | NA | NA |
| TCGA-CZ-4862-01 | 107.46 | 0:DiseaseFree |
| TCGA-CZ-4863-01 | 63.34 | 0:DiseaseFree |
| TCGA-CZ-4864-01 | NA | NA |
| TCGA-CZ-4865-01 | NA | NA |
| TCGA-CZ-4866-01 | 107.33 | 0:DiseaseFree |
| TCGA-CZ-5451-01 | 63.37 | 0:DiseaseFree |
| TCGA-CZ-5452-01 | 58.77 | 0:DiseaseFree |
| TCGA-CZ-5453-01 | NA | NA |
| TCGA-CZ-5454-01 | 13.9 | 1:Recurred/Progressed |
| TCGA-CZ-5455-01 | NA | NA |
| TCGA-CZ-5456-01 | 31.24 | 1:Recurred/Progressed |
| TCGA-CZ-5457-01 | 5.72 | 1:Recurred/Progressed |
| TCGA-CZ-5458-01 | 91.62 | 0:DiseaseFree |
| TCGA-CZ-5459-01 | 55.29 | 0:DiseaseFree |
| TCGA-CZ-5460-01 | 94.38 | 0:DiseaseFree |
| TCGA-CZ-5461-01 | 1.58 | 1:Recurred/Progressed |
| TCGA-CZ-5462-01 | NA | NA |
| TCGA-CZ-5463-01 | 21.75 | 0:DiseaseFree |
| TCGA-CZ-5464-01 | 69.91 | 0:DiseaseFree |
| TCGA-CZ-5465-01 | NA | NA |
| TCGA-CZ-5466-01 | 22.5 | 0:DiseaseFree |
| TCGA-CZ-5467-01 | 2.04 | 1:Recurred/Progressed |
| TCGA-CZ-5468-01 | NA | NA |
| TCGA-CZ-5469-01 | 3.61 | 1:Recurred/Progressed |
| TCGA-CZ-5470-01 | 12.68 | 0:DiseaseFree |
| TCGA-CZ-5982-01 | 80.12 | 0:DiseaseFree |
| TCGA-CZ-5984-01 | 67.9 | 0:DiseaseFree |
| TCGA-CZ-5985-01 | 65.6 | 0:DiseaseFree |
| TCGA-CZ-5986-01 | 12.25 | 0:DiseaseFree |
| TCGA-CZ-5987-01 | 12.39 | 1:Recurred/Progressed |
| TCGA-CZ-5988-01 | 22.77 | 0:DiseaseFree |
| TCGA-CZ-5989-01 | 62.58 | 0:DiseaseFree |
| TCGA-DV-5565-01 | 43.66 | 0:DiseaseFree |
| TCGA-DV-5566-01 | 45.93 | 0:DiseaseFree |
| TCGA-DV-5567-01 | 65.83 | 1:Recurred/Progressed |
| TCGA-DV-5568-01 | 12.16 | 0:DiseaseFree |
| TCGA-DV-5569-01 | 11.66 | 0:DiseaseFree |
| TCGA-DV-5573-01 | 37.12 | 0:DiseaseFree |
| TCGA-DV-5574-01 | 11.73 | 1:Recurred/Progressed |
| TCGA-DV-5575-01 | 56.8 | 0:DiseaseFree |
| TCGA-DV-5576-01 | NA | NA |
| TCGA-DV-A4VX-01 | NA | NA |
| TCGA-DV-A4VZ-01 | 11.99 | 0:DiseaseFree |
| TCGA-DV-A4W0-01 | 65.97 | 1:Recurred/Progressed |
| TCGA-DV-A4W0-05 | 65.97 | 1:Recurred/Progressed |
| TCGA-EU-5904-01 | 18.1 | 0:DiseaseFree |
| TCGA-EU-5905-01 | 3.91 | 0:DiseaseFree |
| TCGA-EU-5906-01 | 6.77 | 0:DiseaseFree |
| TCGA-EU-5907-01 | 4.17 | 0:DiseaseFree |
| TCGA-G6-A5PC-01 | NA | NA |
| TCGA-G6-A8L6-01 | 9.99 | 1:Recurred/Progressed |
| TCGA-G6-A8L7-01 | 70.07 | 0:DiseaseFree |
| TCGA-G6-A8L8-01 | NA | NA |
| TCGA-GK-A6C7-01 | 2 | 0:DiseaseFree |
| TCGA-MM-A563-01 | 19.42 | 0:DiseaseFree |
| TCGA-MM-A564-01 | 19.94 | 0:DiseaseFree |
| TCGA-MM-A84U-01 | 23 | 0:DiseaseFree |
| TCGA-MW-A4EC-01 | 16.36 | 0:DiseaseFree |
| TCGA-T7-A92I-01 | 11.7 | 0:DiseaseFree |
