# Supplementary material for: Pyroptosis-Related lncRNA Prognostic Model for Renal Cancer Contributes to Immunodiagnosis and Immunotherapy
Source: Front Oncol. 2022 Jul 4;12:837155. doi: 10.3389/fonc.2022.837155 (PMC9291251; doi:10.3389/fonc.2022.837155)
Supplement: Supplementary file 10 [file Table_7.docx]

**Supplementary Table S7 c-index of prognostic model**

| Data cohort | c-index | s(c) |
| --- | --- | --- |
| All | 0.76305427 | 0.01978675 |
| train | 0.75527544 | 0.02476586 |
| test | 0.67652349 | 0.03170579 |
